# Photoactivatable Carborhodol and Carborhodamine Dyes with One Cleavable Group: Synthesis, Spectra, and Fluorescence Nanoscopy Applications

**DOI:** 10.1021/jacsau.5c01583

**Published:** 2026-03-04

**Authors:** Taukeer A. Khan, Mariano L. Bossi, Alena Fischer, Vladimir N. Belov, Stefan W. Hell

**Affiliations:** † Department of NanoBiophotonics, 28282 Max Planck Institute for Multidisciplinary Sciences (MPI-NAT), 37077 Göttingen, Germany; ‡ Department of Optical Nanoscopy, 28296 Max Planck Institute for Medical Research (MPI-MR), 69120 Heidelberg, Germany

**Keywords:** photochemistry, super-resolution imaging, photocleavable groups, bioconjugation, fluorescence

## Abstract

Photoactivatable (PA) dyes with symmetric structures and two caging groups, rhodamines and carbo- and silicon-rhodamines, have been widely applied in super-resolution microscopy of subcellular structures with optical resolution far below the diffraction limit. The presence of two “heavy” caging groups reduces the solubility of a probe and, eventually, makes it less biocompatible. The photocleavage in two steps prolongs the photolysis time required for complete PA; it may cause excessive bleaching and secondary photoreactions. Here, we introduce “monocaged” PA fluorescent dyes based on xanthene cores with two heteroatoms (N, O, or N, N). In contrast to standard approaches, we protected only one heteroatom (either N or O) with a photocleavable (4,5-dimethoxy-2-nitrobenzyl) group and demonstrate that it is sufficient to mask the fluorescence of carbo-rhodol and carbo-rhodamine dyes. The monocaged PA probes have significantly lower molecular masses than their analogs with two bulky caging groups. The probes with a reactive group (COOH) provide facile labeling and undergo irreversible single-step photoactivation toward products emitting yellow or orange light. For carborhodol with a free hydroxyl and the protected *N*-methyl group, the reversible increase in emission was found at pH > 7 (as an activation tool, orthogonal to photolysis). Carboxamides incorporating the HaloTag amine (O2) ligand were applied for targeting and imaging of the HaloTag self-labeling enzyme fused with a protein of interest (vimentin). The utility and imaging performance of the probes with two heteroatoms belonging to a fluorophore, but only one caging group, were demonstrated in live cell labeling, conventional (confocal) microscopy, Minimal Photon Fluxes (MINFLUX) nanoscopy, and single-molecule localization methods (SMLM).

## Graphical abstract

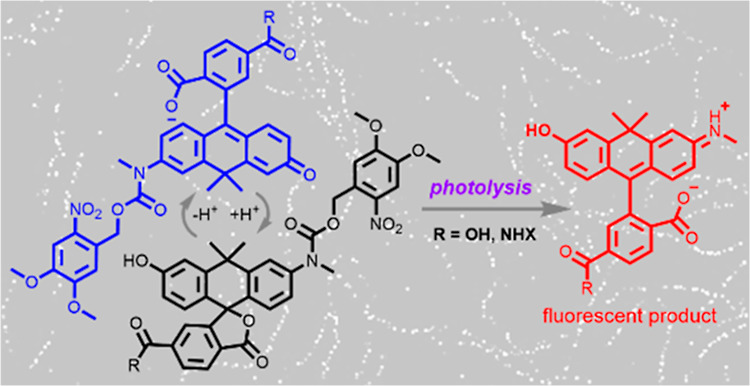

## Introduction

Super-resolution optical microscopy provides a powerful tool for imaging of subcellular structures and processes with optical resolution far below the diffraction limit.^1–4^ Nanoscopy techniques, such as MINFLUX^5–7^ and SMLM,^8,9^ allow one to measure distances between labels, assess range distribution, count labeled sites within one entity, or track molecular motions with unprecedented spatiotemporal resolution.^10–12^ In these methods, the markers are converted from a dark to a bright (fluorescent) state in such a way that only single emitters (or sparse subsets of emitters) turn out to be in the bright state. Therefore, a proper choice, design, and synthesis of an optimal probe, in respect of emission color, PA wavelength, PA quantum yield, number of emitted photons, “blinking” behavior, as well as the presence of a reactive group, play a key role.^13^

In the development of next-generation organic fluorophores for MINFLUX and MINSTED nanoscopies,^5,14,15^ irreversible photoactivation of “masked’’ dyes may offer advantages over reversible switching between “dark” and “bright” states or “blinking’’ events.^16^ Photoactivation can be controlled and induced externally by irradiation with a single wavelength, while reversible switching requires dyes with more complex structures and the use of two light sources. The use of “blinking’’ dyes (cyanines and oxazines) in SMLM is only possible in the presence of special buffers and reducing agents.^17–19^ In contrast, *o*-nitrobenzyl groups (and their ring-substituted analogs) are often chosen as photolabile protecting groups, which can be irreversibly cleaved off under UV light irradiation (365/405 nm).^20,21^

2-Nitrobenzyl (oNB) “cages’’ and their 4,5-dimethoxy derivatives are structurally simple and can be easily introduced.^22,23^ However, their presence makes photoactivatable probes more hydrophobic, less soluble in water, and prone to aggregation; all this can complicate bioconjugation procedures and result in low labeling density.^24^ To alleviate these issues and increase the polarity of PA probes, a silicon rhodamine-based fluorophore with two caging groups and polar carboxamide residues was synthesized and applied in MINFLUX nanoscopy.^25^ Other analogs having [2-nitro-5-(2-sulfethyl amino)­carbonyl]­benzyl residues showed improved solubility in water compared to unsubstituted *o*NB caging groups,^26^ but their net negative charge precludes the use in live-cell applications. Importantly, all PA rhodamines, carbo- and silicon-rhodamines applied in super-resolution microscopy so far, have two caging groups and possess a molecular symmetry plane. Two obvious reasons for pursuing this double-cage strategy are due to synthetic simplicity and the assumption (yet unproved) that complete spiro-lactonization, i.e., “locking” the probe in a colorless closed-ring form, is secured by incorporation of two (not one!) groups, urethane, carbonate, or ether, protecting nitrogen or oxygen atoms attached to the dye core. The presence of two caging groups increases the molecular mass, makes the dye structure more symmetric, reduces the solubility, and, eventually, makes the initial probe less biocompatible. The photocleavage in two steps may unnecessarily prolong the photolysis time required for complete PA, causing excessive bleaching and secondary photoreactions.

In a quest for photoactivatable probes with improved properties, we sought to overcome these drawbacks by considering monocaged photoactivatable dyes with asymmetric structures.^27,28^ The presence of one caging group not only significantly decreases the molecular mass but also facilitates faster release of the fluorescence dye in response to light irradiation and reduces the amount of potentially harmful 2-nitrosobenzaldehyde (photolysis byproduct). The one-step cleavage shortens the overall irradiation time and minimizes cell damage.^29^ As monocaged PA dyes, we considered xanthenes with two electron-donor atoms – *N*, *N*, or *N*, *O* in rhodamines^30,31^ or carbo-rhodols.^32^ These are bright, photostable, and widely used dyes having high extinction coefficients. Importantly, the nitrogen atoms in these structures are, in general, more nucleophilic than oxygens. Therefore, *N*-carbamoylation is expected to occur more readily than the formation of carbonates. While a few monocaged PA rhodamines^27^ and fluoresceins^28^ containing an *o*NB caging group have been reported in the literature, the parent fluorophore in them was modified already before “masking”. In a rhodamine, one N atom was incorporated into a urea fragment.^27^ In fluorescein, one oxygen atom was alkylated by omega-carboxyalkyl residue.^28^ Therefore, upon cleavage of the *o*NB group, the resulted dyes were not “true’’ rhodamines or fluoresceins but rather *N*-ureido rhodamine or *O*-[(omega-carboxyl)­alkyl] fluorescein derivatives, with fluorescent quantum yields lower than that of the parent free dyes.

Taking into consideration the pieces of evidence and factors mentioned above, we prepared photoactivatable dyes with two heteroatoms (*N*, *N* and *N*, *O*) and one caging group attached to a more nucleophilic *N*-atom (Figure 1). We used the 4,5-dimethoxy-2-nitrobenzyl caging group, which can be cleaved by irradiation not only with UV-light (365 nm) but also with violet light (405–440 nm). We chose carbo-rhodol^33^ and carborhodamine^31^ systems, which provide yellow-orange emission upon photoactivation (Figure 1).

**Figure 1:**
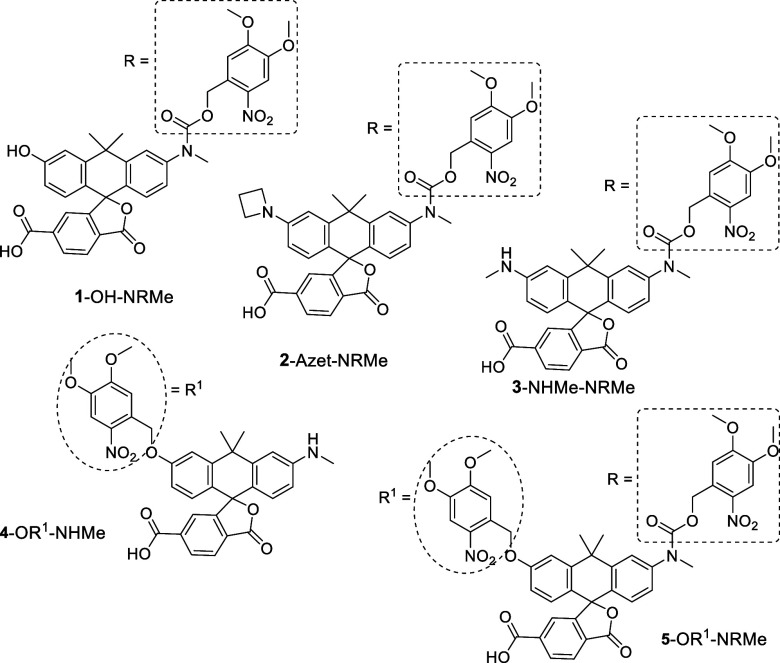
Mono- and bis-caged photoactivatable carborhodol and carborhodamine dyes having 4,5-dimethoxy-2-nitrobenzyl (shown in oval) or/and 4,5-dimethoxy-2-nitrobenzyloxycarbonyl (shown in rectangle) photocleavable groups.

The presence of only one caging group is interesting, as it may provide a compound with (weak) blue-shifted emission that can be observed prior to photolysis. This phenomenon is likely to occur due to less efficient, but non-negligible stabilization of the “open’’ fluorescent form, expected to be present in the equilibrium with the “closed’’ nonfluorescent structure (see Scheme 1). The pH dependence of the emission will be discussed below.

**Scheme 1:**
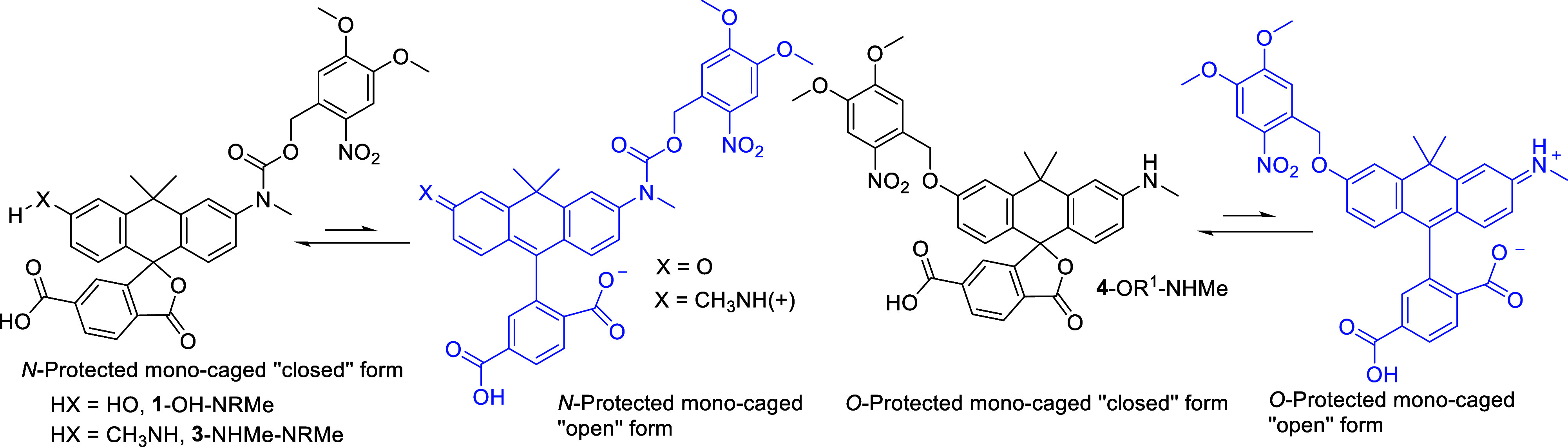
Envisaged Equilibrium between ‘‘Closed’’ and ‘‘Open’’ Forms of Monocaged Carborhodols **1**-OH-NRMe, **4**-OR1-NHMe, and Carborhodamine **3**-NHMe-NRMe^a^

a For compound **1**-OH-NRMe (X = O), the equilibrium between HO-C_ar_ (closed-ring isomer) and O=C_ar_ (open-ring form) involves deprotonation–protonation. For pH-dependent emission of compound **1**-OH-NRMe (X = O), see Figure 3.

## Results and Discussion

Based on the factors mentioned above and the synthesis feasibility, we have chosen and prepared three monocaged photoactivatable dyes: one carborhodol and two carborhodamines (Figure 1) having various emission colors. Rhodol dyes are hybrids of fluorescein and rhodamine; they contain one oxygen and one nitrogen atoms attached to C-3′ and C-6′ of fluorophore.^34–36^ The probe **1**-OH-NRMe is carborhodol, in which the oxygen atom of the xanthene fragment is replaced with the group > C­(CH_3_)_2_, as in carbopyronines. This replacement provides red-shift of ca. 50 nm in absorption and emission bands.^31–33^ Additionally, carborhodols are distinguished by larger Stokes shifts than those observed for the parent fluorescein and carbopyronine dyes.^37^ In the context of super-resolution microscopy, rhodols and carborhodols have been mentioned in three reports.^33,38,39^

Fortunately, (carbo)­rhodol dyes are promising due to their photostability, brightness, zero net charge, balanced polarity, pH tolerance, and sensitivity of spectra to structural changes.^30,32–37^ These were the reasons for choosing and preparing the masked carborhodol **1**-OH-NRMe (Figure 1). Apart from it, we have chosen and prepared two monocaged carborhodamines: **2**-Azet-NRMe having azetidine and *N*-methylamino residues, and **3**-NHMe-NRMe containing two *N*-methylamino groups attached to a carborhodamine core (Figure 1). Upon photoactivation, compound **2**-Azet-NRMe generates the dye with the most red-shifted emission (of all three probes). Compound **3**-NHMe-NRMe is a monocaged version of 580CP dye^31^ with absorption and emission maxima at 580 and 607 nm, respectively.

### Synthesis

To prepare xanthene-based monocaged photoactivable dyes with a ‘‘remote’’ carboxylic ester group, ditriflate with a spirolactone moiety (**6**) was selected as the starting material.^25^ All synthesis routes in Schemes 2, 3, and 4 involved compound **6**. The first target **1**-OH-NRMe (Scheme 2) was synthesized from ditriflate **6** and carbamate **7** in the course of a one-step Pd-catalyzed Buchwald–Hartwig (mono)­amination. With one carbamate group introduced, compound **8**-Tf-O^t^Bu contained all of the functionalities required for the syntheses of two targets (Schemes 2, 3). The sequential cleavage of triflate and *tert*-butyl ester groups in the presence of TBAF_*_3H_2_O and trifluoroacetic acid afforded the target monocaged PA carbo-rhodol with a 6′-COOH group (**1**-OH-NRMe, Scheme 2). The availability of 6′-COOH makes further derivatization possible. However, the presence of the relatively nucleophilic HO group in compound **1**-OH-NRMe has to be taken into account, as it might bind with the carboxylate group (especially as phenolate) if another reaction partner is a weaker nucleophile. Therefore, for obtaining probe **8**-H-HT, we did not start from compound **1**-OH-NRMe. Instead of that, the *tert*-butyl ester group in compound **8**-Tf-O^t^Bu was cleaved off in the reaction with trifluoroacetic acid (TFA); then acid **8**-Tf-OH was bound with HaloTag amine (O2), the aryl triflate group hydrolyzed, and thus, the probe **8**-H-HT was prepared (Scheme 2). In a rational way, intermediate **10** was synthesized from compound **8**-Tf-O^t^Bu and azetidine (Scheme 3). The cleavage of the *tert*-butyl ester group in intermediate **10** afforded the photoactivatable monocaged dye **2**-Azet-NRMe directly (Scheme 3). To prepare the third target (**3**-NHMe-NRMe), we again started from ditriflate **6** (Scheme 4). In a two-step Buchwald–Hartwig amination, we first applied *tert*-butyl-*N*-methylcarbamate and then carbamate **7**, so that intermediate **13** having two urethane groups was formed (Scheme 4). In the final steps, probe **3**-NHMe-NRMe was obtained by cleaving two acid-sensitive protecting groups. Upon photoactivation, the monocaged probe **3**-NHMe-NRMe gives the bright and useful fluorescent dye 580CP.^31^ Amidation of carboxylic acids **2**-Azet-NRMe and **3**-NHMe-NRMe with HaloTag amine (O2), as a ligand binding with the HaloTag protein, was performed as shown in Schemes 3 and 4.

**Scheme 2:**
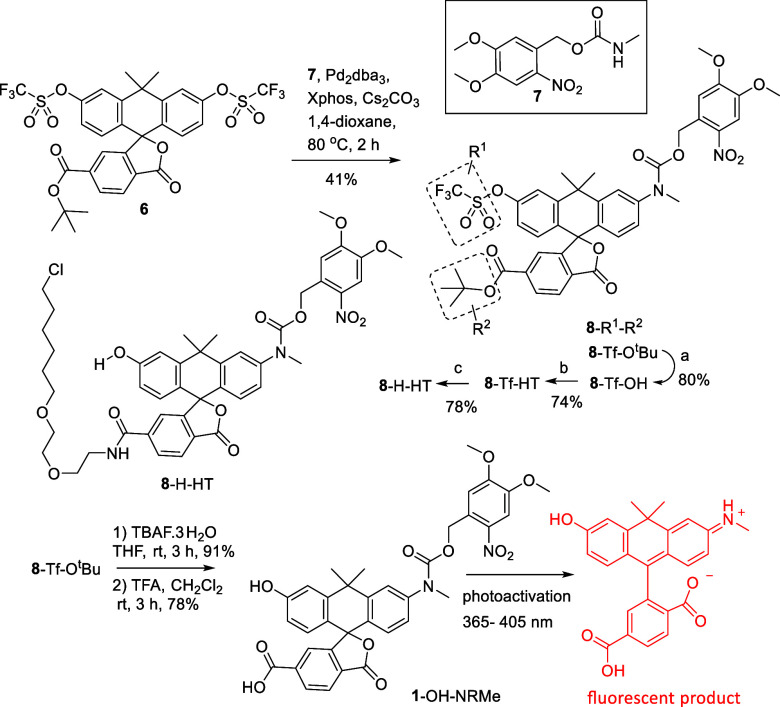
Monocaged Carboxylic Acid **1**-OH-NRMe and Its HaloTag Ligand Derivative **8**-H-HT^a^

a Reaction Conditions: (a) TFA, CH2Cl2, rt, 3 h; (b) H2N­(CH2)­2O­(CH2)­2O­(CH2)­6Cl, DIEA, PyBOP, DMF, rt, 4 h; (c) TBAF*3H2O, THF, rt, 3 h.

**Scheme 3:**
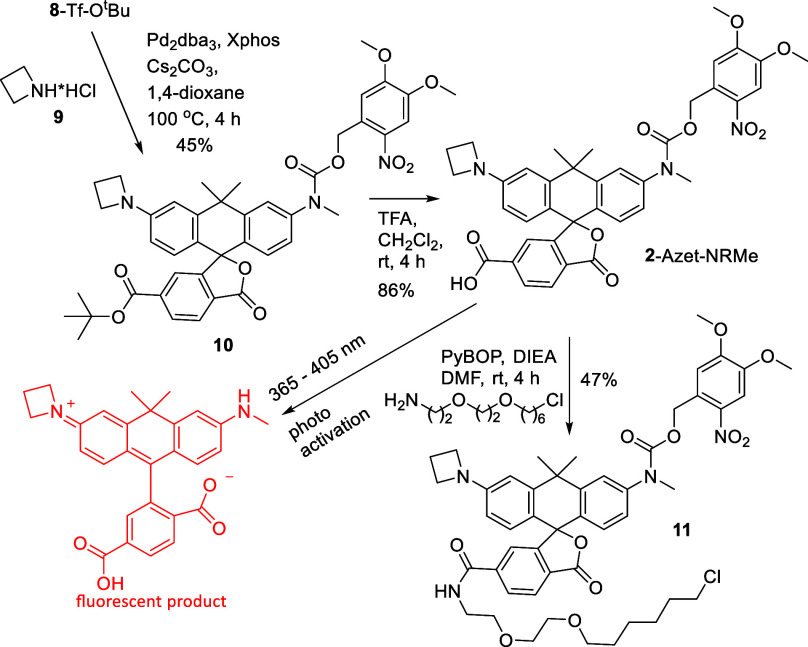
Synthesis of Monocaged Carboxylic Acid **2**-Azet-NRMe and Its HaloTag Ligand Derivative **11**

**Scheme 4:**
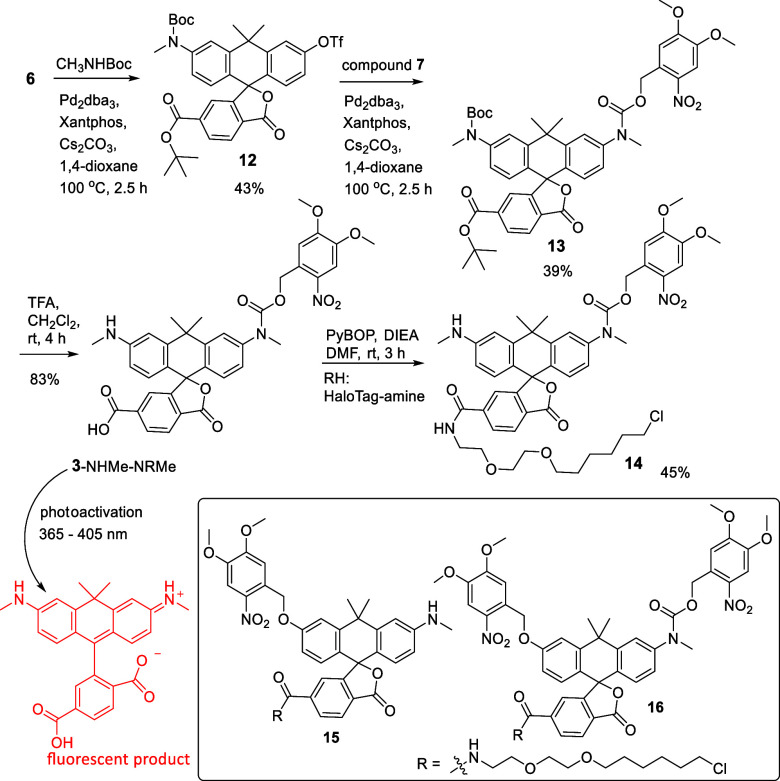
Photoactivatable Monocaged Carboxylic Acid **3**-NHMe-NRMe and Its HaloTag Ligand Derivative **14** ^a^

a Amides **15** and **16** are HaloTag probes obtained from Compounds **4**-OR^1^-NHMe and **5**-OR^1^-NRMe (Figure 1).

First, we studied the photophysical properties and photoactivation of three monocaged compounds **1**-OH-NRMe, **2**-Azet-NRMe, and **3**-NHMe-NRMe (Table 1) in cuvette experiments. Then we applied their HaloTag amides in the labeling of live cells, followed by photoactivation and imaging. By this, we learned that the carborhodol monocaged probe **8**-H-HT (Scheme 2) outperformed compounds **11** (Scheme 3) and **14** (Scheme 4). This prompted us to prepare and study the properties of two additional caged carborhodols **4**-OR^1^-NHMe and **5**-OR^1^-NRMe (Figure 1) and their HaloTag amides **15** and **16** (Scheme 4) as photoactivatable dyes structurally related to compounds **1**-OH-NRMe and **8**-H-HT (Scheme 2), in which the caging group was attached to nitrogen. In compound **4**-OR^1^-NHMe, the caging group was attached to the oxygen atom, and in compound **5**-OR^1^-NRMe, the caging groups were attached to both heteroatoms (Figure 1). The scheme and experimental procedures for the synthesis of compounds **4**-OR^1^-NHMe and **5**-OR^1^-NRMe and their HaloTag amides **15** and **16** are given in Supporting Information (Schemes S5 and S6).

**Table 1:** Photophysical Properties of the Initial Caged Dyes and Fluorescent Products

compound	caged (close form)			fluorescent product	
	λ^Abs^/ε (nm/M^–1^ cm^–1^)^a^	ε_SC_^365nm^ (M^–1^ cm^–1^)^b^	ϕ_PA_ ^c^	λ_FP_^Abs^/ε_FP_ (nm/M^–1^ cm^–1^)^d^	λ_FP_^Em^ (nm)^e^
**1**-OH-NRMe	350/4800	4200	4.0 × 10^–3^	566/55700	595
**2**-Azet-NRMe	347/4800	4100	4.0 × 10^–3^	594/63200	620
**3**-NHMe-NRMe	350/4600	4100	nonmonoexp	585/86000	608
**4**-OR^1^-NHMe	349/5600	4600	2.4 × 10^–2^	566/43000	595
**5**-OR^1^-NRMe	352/8700	7600	7.6 × 10^–2^ 3.5 × 10^–3^	566/48000	595

a Extinction coefficients (ε) of the starting compound (SC) at λ_abs_^max^.

b ε values of SC at 365 nm photolysis wavelength.

c Photoactivation quantum yields (ϕ_PA_).

d Absorption max. (λ_Fl_^Abs^) and ε values of the fluorescent product (FP).

e Emission max. (λ_Fl_^Em^) of FP.

### Photochemical and Photophysical Properties of Caged Dyes

We examined the photoactivation of all caged dyes (**1**-OH-NRMe, **2**-Azet-NRMe, **3**-NHMe-NRMe, **4**-OR^1^-NHMe, **5**-OR^1^-NRMe) in aq. buffer DMSO (80:20) solutions under irradiation with 365 nm light. We observed significant increase in the absorption and emission intensities in the visible region, indicating the progress in photolysis and conversion into the fluorescent product(s) (see Figures 2 and S1–S5).

**Figure 2:**
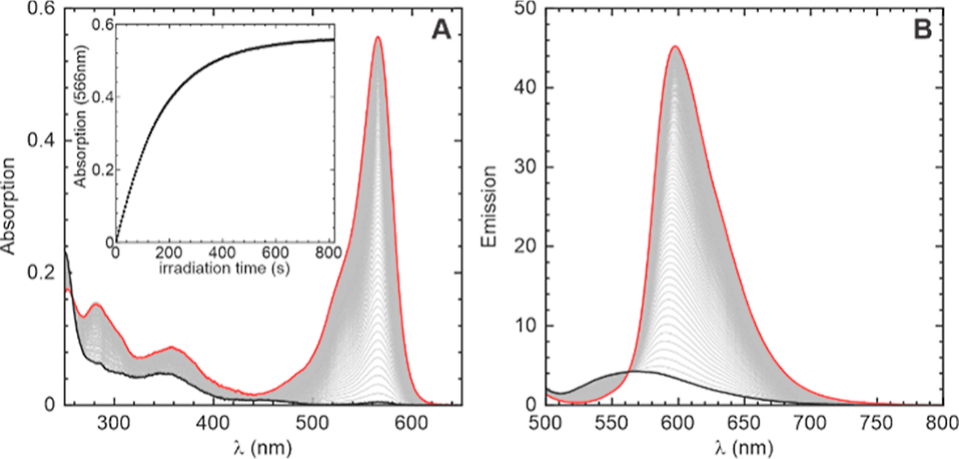
Photolysis of monocaged dye **1**-OH-NRMe in aq. buffer/DMSO (80:20) solution. Increase in absorption (A) and emission (B) intensities in time (0–800 s). The spectra before irradiation and after complete uncaging are plotted with black and red lines, respectively. The inset in (A) displays the transient at the absorption maximum of the fluorescent product (566 nm). Data for compounds **2**-Azet-NRMe, **3**-NHMe-NRMe, **4**-OR^1^-NHMe, and **5**-OR^1^-NRMe are given in Sup. Inf (Figures S2–S5).

The course of photoactivation was monitored by measuring the absorption and emission spectra. The initial solutions and the photolyzed mixtures were also analyzed by means of LCMS. Under these conditions, all photoactivation reactions except one (**2**-Azet-NRMe, which displayed azetidine ring-opening products presumably caused by C–N bond cleavage followed by addition of water; see Figure S2) were clean. In each case, we detected peaks of the starting compounds and the corresponding fully uncaged fluorescent dyes (Figures S1–S5). The photophysical properties of compounds **1**-OH-NRMe, **2**-Azet-NRMe, **3**-NHMe-NRMe, 4-OR^1^-NHMe, and **5**-OR^1^-NRMe and the fluorescent products obtained from them after photoactivation are summarized in Table 1. Remarkably, *N*-caged compounds undergo photoactivation with quantum yields of about 10^–3^, while *O*-caged compounds present higher values (around 10^–2^). Not surprisingly, bis-caged compound **5**-OR^1^-NRMe presents two distinct values in these ranges (Table 1). The irregularity was found for compound **3**-NHMe-NRMe which, despite having only one caging moiety, displays nonmonoexponential behavior. A possible explanation is that this compound may undergo disproportionation to uncaged and bis-caged compounds (parallel to direct photocleavage to the final free dye). The photoactivation quantum yields are relatively low. However, these values are in the same range as reported for the other photoactivatable fluorescent dyes (with various photocleavable residues), such as Rhodamines NN,^40–42^ silicon-rhodamines,^43^ thiocarbonyls,^44^ azido-push–pull dyes,^45^ and BODIPYs.^46^ Among them, coumarin-derived caging groups enable cleavage and release of the dye (or another molecule) under irradiation with visible (green) light or in the course of a two-photon process (with very powerful far-red or IR lasers).^47–49^

### pH Dependence of the Emission Spectra

As mentioned above, we expected that the monocaged dyes may have weak and blue-shifted emission due to the presence of open-ring isomers in the equilibrium with dominating closed-ring isomers (Scheme 1). Indeed, monocaged rhodols (**1**-OH-NRMe and **4**-OR^1^-NHMe) exhibited weak fluorescence at pH 7 (Figures 2B and S4). To study pH dependence related to equilibrium shown in Scheme 1, we measured the emission spectra of monocaged dyes in aq. PBS/DMSO (80:20) solutions at various pH values in the range of 4–10 (Figures 3 and S6). The emission of monocaged rhodol (**1**-OH-NRMe, Scheme 1), which has a free phenolic hydroxyl group, was weak at pH < 7 but sharply increased at basic pH, so that a broad emission band maximum emerged at about 575–580 nm (Figure 3, upper part). Compound **1**-OH-NRMe (in its “phenolic part”) is structurally similar to fluorescein. The increase in absorption and emission of fluorescein is observed in alkaline aqueous solutions.^50^ Thus, the same trend as that for fluorescein was observed for compound **1**-OH-NRMe, which is not surprising. It can be attributed to deprotonation of phenolic OH groups, occurring well beyond pH 7. The deprotonation of HO-C_ar_ (closed-ring isomer of **1**-OH-NRMe) and the structure assigned to the open-ring form are given in Scheme 1. This effect was found to be reversible, and the fluorescence of **1**-OH-NRMe weakened upon returning to neutral (intracellular) and slightly acidic pH. This feature may represent an interesting option to reversibly modulate the fluorescence output by changing pH. This phenomenon may be applied to pinpoint the labeled sites prior to PA, e.g., in multimodal imaging experiments. Other monocaged dyes (**2**-Azet-NRMe, **3**-NHMe-NRMe, **4**-OR^1^-NHMe) were found to be insensitive to pH changes and showed negligible emission in the studied pH range (Figures 3 and S6).

**Figure 3:**
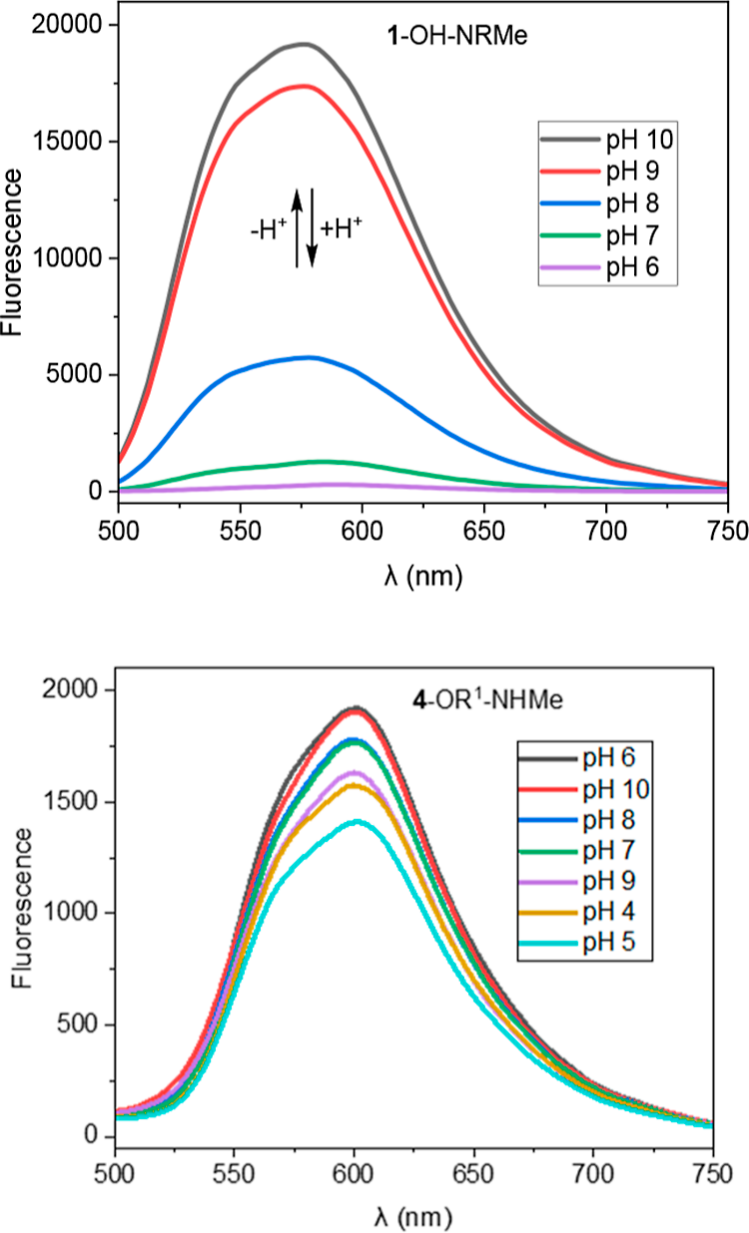
Fluorescence spectra of monocaged carboxylic acids **1**-OH-NRMe (upper plots) and **4**-OR^1^-NHMe (lower plots) at different pH values. The plots for compounds **2**-Azet-NRMe and **3**-NHMe-NRMe are given in Supporting Information (Figure S6).

### Bioimaging

Encouraged by the efficient and reliable photoactivation performance of all the studied monocaged compounds (and the reference bis-caged compound **5**-OR^1^-NRMe), we prepared amides **8**-H-HT, **11**, **14**, **15**, and **16** incorporating HaloTag amine residue bound with carboxylic acid groups in caged fluorophores and applied these probes in live-cell labeling and imaging. For that, we used U2OS cells stably expressing a vimentin–HaloTag fusion construct^51^ and applied the standard protocol.^52^ Each amide was dissolved in the cell medium at a 500 nM concentration, and cells were incubated for 1 h. The samples were washed and mounted in fresh medium (without the probe), and the samples were observed in a confocal microscope. Images were acquired before and after photoactivation with a 405 nm laser (Figures 4 and S7–S10). In viability tests (Figure S11), we did not observe any (photo) toxic effects induced by the caged dyes, the irradiation procedure (for uncaging and excitation), the presence of the newly formed dyes, and byproducts (e.g., 2-nitrosobenzaldehyde) for all probes (**8**-H-HT, **11**, **14**, **15**, **16**) under the applied labeling conditions. A highly selective binding with the target protein and a large signal increase (high contrast) were observed upon photoactivation. This confirms the cell permeability of all HaloTag probes and the fact that the uncaging with violet (405 nm) light can be readily performed after binding to the host protein. By comparing the confocal images, we concluded that compound **8**-H-HT showed the best performance among all monocaged probes and the highest contrast, comparable to the one observed with the bis-caged analog **16**.

**Figure 4:**
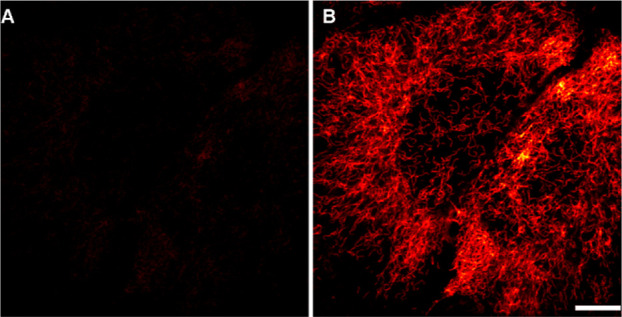
Confocal image of live U2OS cells with the vimentin–HaloTag fusion protein upon labeling with compound **8**-H-HT: (A) before and (B) after activation with 405 nm light. Scale bar: 10 μm. Confocal images with compounds **11**, **14**, **15**, and **16** are given in Supp. Inf (Figures S7–S10).

We further explored the utility of compound **8**-H-HT in super-resolution microscopy based on the activation and detection of single fluorophores. The samples were labeled under the same conditions, as in the experiments involving confocal microscopy and fixed to prevent the loss of spatial resolution due to the movements in the structure during image recording.^25^ We first acquired SMLM images in a TIRF microscope, with excitation at 561 nm, detection in the 580–620 nm range, and gradually increasing activation power at 405 nm. After a few preactivated molecules (used for finding the sample and focusing) were bleached, the single molecule regime was rapidly achieved. Images of vimentin filaments were obtained (Figure 5), with an average localization uncertainty of 12–14 nm (median-mean) and an average of 2800 photons per single molecule event. Similar results were obtained when the samples were mounted on Mowiol (Figure S12).

**Figure 5:**
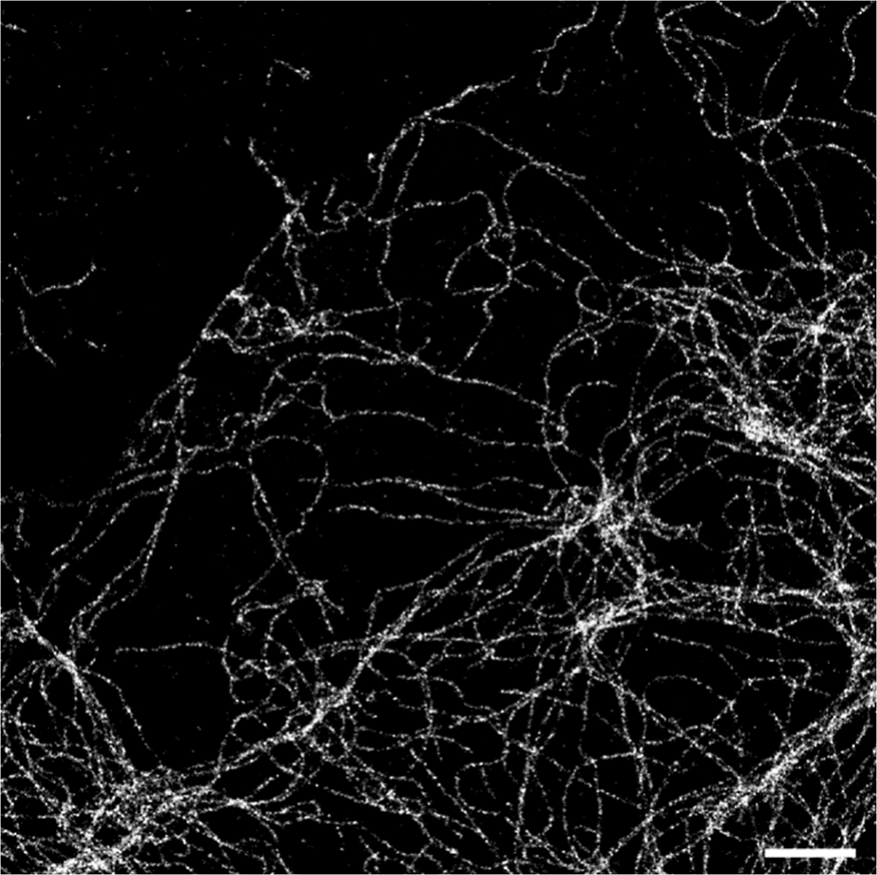
SMLM image of fixed U2OS cells expressing the vimentin–HaloTag construct and “live labeled” with compound **8**-H-HT (500 nM, 1 h), mounted in aqueous PBS buffer. Scale bar: 5 mm.

We finally assessed the applicability of the monocaged rhodol probe, **8**-H-HT, in MINFLUX nanoscopy, an advance technique that localizes individual fluorophores using an excitation beam with an intensity minimum and allows to reach single-digit nanometer resolution.^5–7^ The samples were prepared with the same protocol as the one used for SMLM imaging, with the only difference being that gold beads were added to achieve active 3D-stabilization of the sample in the microscope. Preactivated molecules of the probe allowed for the acquisition of a fast (low resolution) confocal image (Figure 6A). Then, a MINFLUX image was acquired with 560 nm excitation and detection in the 580–630 nm range (Figure 6B). A localization precision of 2.3 nm (Figure 6D) was achieved using 130 photons on average (Figure 6E), when a photon threshold of 100 photons was selected in the MINFLUX localization routine. Under these conditions, activated single molecules were localized 13–22 times (median-mean) to yield on average a total of 4100–6500 photons (median-mean; Figure 6F,G). These values and image quality are in accordance with previous results obtained with double-caged carbo- and silicon-rhodamines.^25^

**Figure 6:**
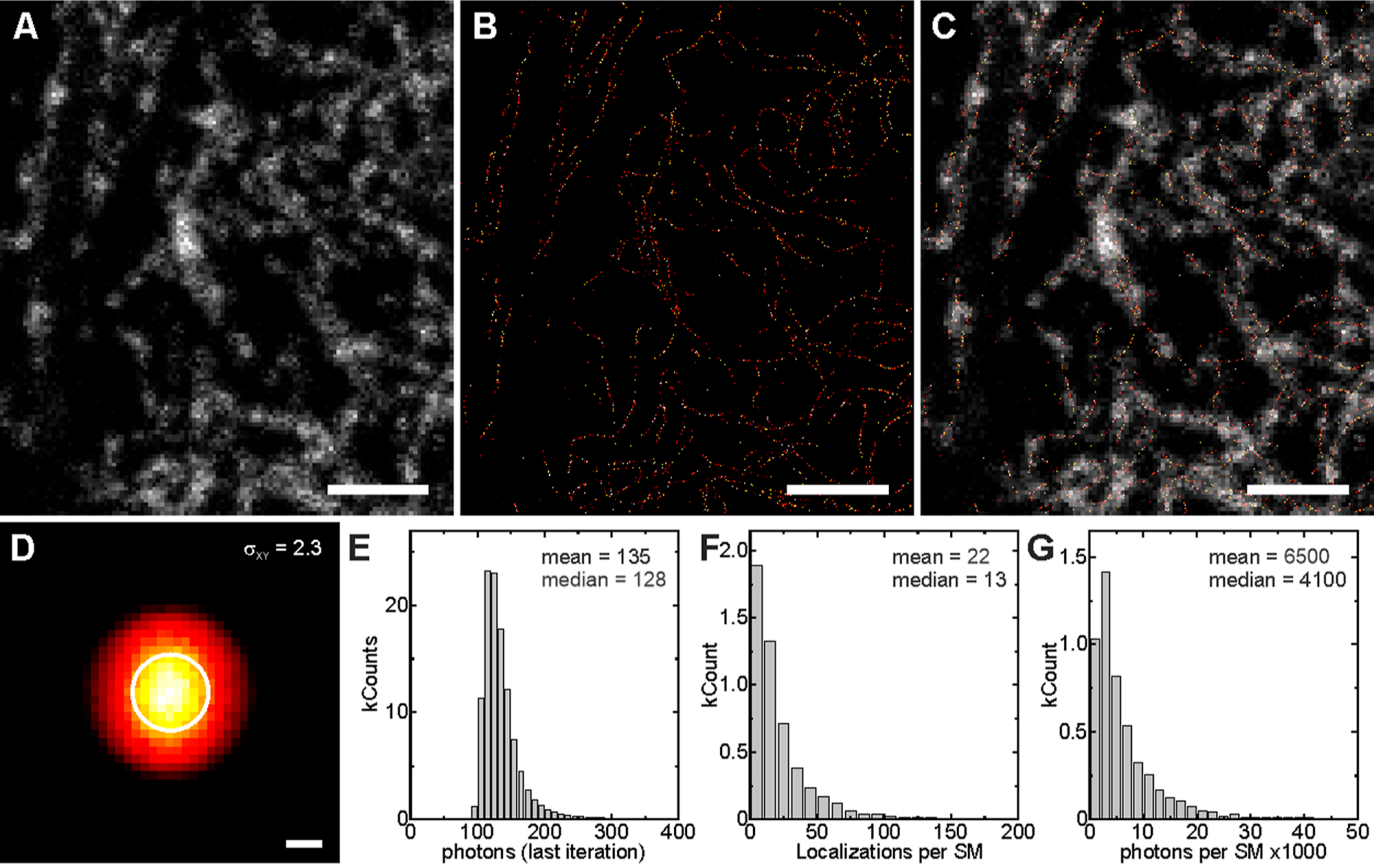
MINFLUX imaging with compound **8**-H-HT on fixed U2OS cells expressing the vimentin–HaloTag construct (labeling was performed in live cells at 500 nM for 1 h). (A) Confocal image recorded before the acquisition of the MINFLUX image, with the few markers activated during sample manipulation. (B) MINFLUX image. (C) Combined image. (D) Histogram of the localization spread around their mean value (the mean precision indicated with a circle of *r* = σ_*x*,*y*_ obtained from a 2D Gaussian fit). (E) Histogram of the number of photons used in the final iteration of the MINFLUX localization. (F) Histogram of the number of localizations obtained from a single molecule “on”-event. (G) Histogram of the total number of photons obtained per single molecule “on”-event, in all iterations of the MINFLUX localization. Mean and median values are reported in E–G. Scale bars: (A–C) 2 μm; (D) 2 nm.

## Conclusion

In the development of advanced fluorescent markers for MINFLUX, MINSTED, and SMLM nanoscopies, photoactivatable markers offer some advantages over reversibly switchable dyes. Their structures are simpler, and the fluorophore types prone to “caging” (masking) of their emission are much more abundant than the dye structures showing reversible modulation of the fluorescence signal. 3,4-Dimethoxy-2-nitrobenzyl groups, which are often chosen for photolabile protection, confer undesirable properties upon introduction. They increase hydrophobicity, structure rigidity (for xanthenes, rhodols, and carbododols, as most popular frameworks), and bulkiness and reduce bioavailability, which results in lower labeling efficiency. To overcome these drawbacks, we conceived and prepared monocaged photoactivatable dyes bearing one caging group attached to carborhodol and carborhodamine scaffolds. Compared with bulkier symmetric analogs, they have asymmetric structures with reduced molecular masses and exhibit smooth release of the fluorescent product upon irradiation with 405 nm light. For N-protected carborhodol dye **1**-OH-NRM, we observed weak green-shifted emission already in the initial state (prior to photolysis). It was attributed to the presence of the “open” form of this probe with an extended pi-conjugated electron system. Remarkably, nonirradiated (uncaged) compound **1**-OH-NRMe displayed much stronger emission at higher pH (>7). This effect was found to be reversible, and the fluorescence of **1**-OH-NRMe weakened again upon returning to neutral and slightly acidic pH. This phenomenon may be used to pinpoint the labeled sites prior to photoactivation, e.g., in multilabel and/or multimodal imaging experiments.

All probes contained a free carboxylic acid group required for bioconjugation. By this, we transformed the parent carboxylic acids into amides incorporating HaloTag ligands. The amides were used in live cell experiments to label Vimentin–HaloTag fusion proteins. Upon labeling and photoactivation with 405 nm light, the cells were imaged in a confocal microscope. We observed selective binding with the target protein, high fluorescence gain (comparing pre- and postactivation), and low background signal (overall in the initial state and in areas without labels). We applied monocaged probes in super-resolution MINFLUX and SMLM microscopy methods. The combination of organic synthesis, optical spectroscopy, bioconjugation, and molecular biology techniques (i.e., routines involving stable cell lines with endogenously tagged proteins) is likely to provide further advances in bioorganic and physical chemistry, super-resolution microscopy, and natural sciences.

## Supplementary Material



## Notes

Photolysis equipment, irradiation conditions, and reaction monitoring by means of LC–MS; cell staining conditions; sample preparation (for microscopy); confocal, SMLM, and MINFLUX imaging conditions; microscopic images; experimental section including synthesis and characterization data of all new compounds; and copies of NMR spectra (PDF)
