# Supplementary material for: Photoactivatable Carborhodol and Carborhodamine Dyes with One Cleavable Group: Synthesis, Spectra, and Fluorescence Nanoscopy Applications
Source: JACS Au. 2026 Mar 4;6(3):1693–701. doi: 10.1021/jacsau.5c01583 (PMC13014241; doi:10.1021/jacsau.5c01583)
Supplement: Supplementary file 1 [file au5c01583_si_001.pdf]

Supporting Information

Photoactivatable Carborhodol and Carborhodamine Dyes with One Cleavable Group: Synthesis, Spectra and Fluorescence Nanoscopy Applications

Taukeer A. Khan,[1] Mariano L. Bossi,[2]* Alena Fischer,[2] Vladimir N. Belov,[1]*

Stefan W. Hell[1,2]*

[1] Department of NanoBiophotonics, Max Planck Institute for Multidisciplinary Sciences (MPI-NAT), 37077 Göttingen, Germany

[2] Department of Optical Nanoscopy, Max Planck Institute for Medical Research (MPI-MR), 69120 Heidelberg, Germany

**Corresponding authors: vladimir.belov@mpinat.mpg.de,; mariano.bossi@mr.mpg.de, stefan.hell@mpinat.mpg.de*

Keywords: *Photochemistry • Super-resolution imaging • Photocleavable groups • Bioconjugation • Fluorescence*

Table of Contents

Photolysis of **1**-OH-NRMe**, 2**-Azet-NRMe**, 3**-NHMe-NRMe, **4**-OR^1-NHMe**, 5**-OR^1-NRMe S2

Absorption spectra **2**-Azet-NRMe and **3**-NHMe-NRMe at different pH values S7

HaloTag Staining, Sample Preparation S8

Confocal Imaging S8

Cell viability test S10

SMLM Imaging S11

MINFLUX Imaging S12

General experimental information and synthesis S13

Scheme S1 S14

Scheme S2 S16

Scheme S3 S19

Scheme S4 S22

Scheme S5 S25

Scheme S6 S28

References S31

Copies of NMR Spectra S32–S60

Photolysis of caged dyes with free carboxylic acid groups: 1-OH-NRMe**, 2**-Azet-NRMe**, 3**-NHMe-NRMe, **4**-OR[1]-NHMe and **5**-OR[1]-NRMe

Stock solutions (2 mM) of compounds **1**-OH-NRMe**, 2**-Azet-NRMe**, 3**-NHMe-NRMe**, 4**-OR[1]-NHMe and **5**-OR[1]-NRMe were prepared in dimethyl sulfoxide. Final dilutions for spectroscopic characterization and irradiation experiments were prepared in a 2:8 mixture of dimethyl sulfoxide and phosphate buffer (pH = 7.0, 100 mM) at a dye concentration of approximately 10 µM. All measurements were performed in quartz cuvettes with four clear faces (Hellma GmbH & Co.). Irradiation experiments (Figure 2, main text and Figures S1-S5) were performed on a home-build setup,[1] using a 365 nm LED as an irradiation source (M365-L2, Thorlabs), a Deuterium/Xenon lamp (DH-2000-BAL, Ocean Optics) as an illumination source and a diode array spectrometer (FLAME-S-UV-VIS-ES, Ocean Optics). The intensity of the irradiation light was calibrated with a chemical actinometer (azobenzene in MeOH). The samples were kept at 20 °C and continuously stirred with a Peltier-based temperature control (Luma 40, Quantum Northwest, Inc.). The absorption of the samples was measured at a right angle with respect to the irradiation source, at fixed irradiation intervals, until complete conversion to the final product was observed. Excitation was performed with a short pulse of an LED source (470 nm or 530 nm) at a right angle, with respect to the fluorescence detection. The solutions obtained upon completion of the photolysis experiments were analysed by means of LC-MS (Shimadzu LCMS-2020).

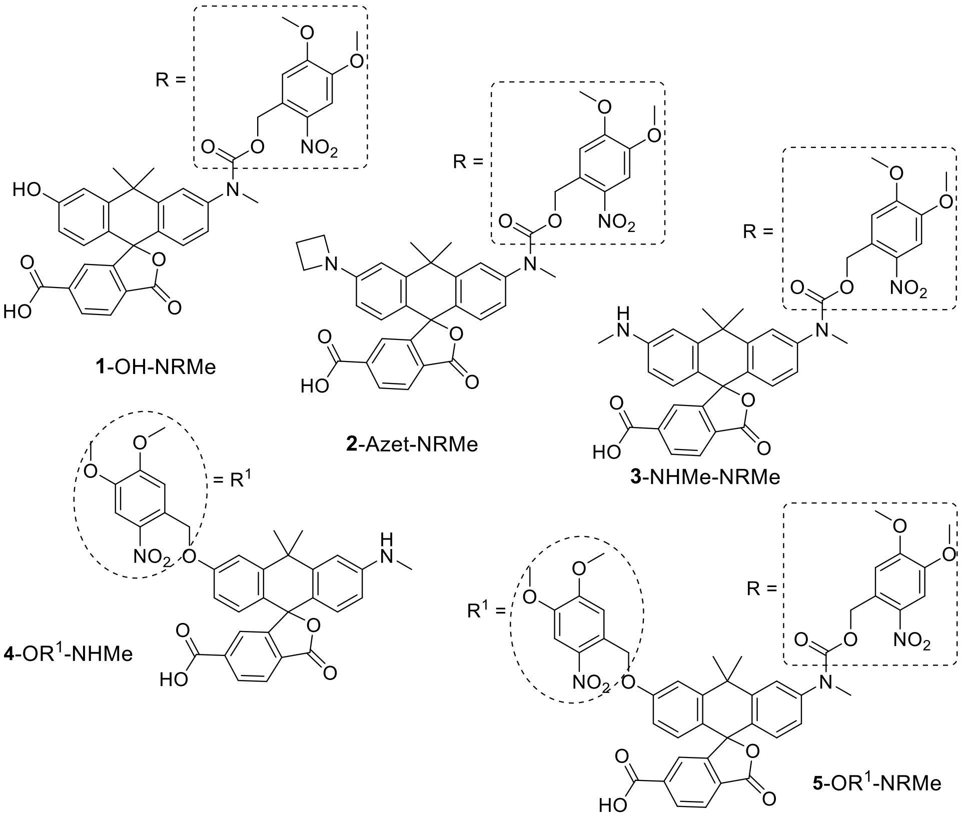

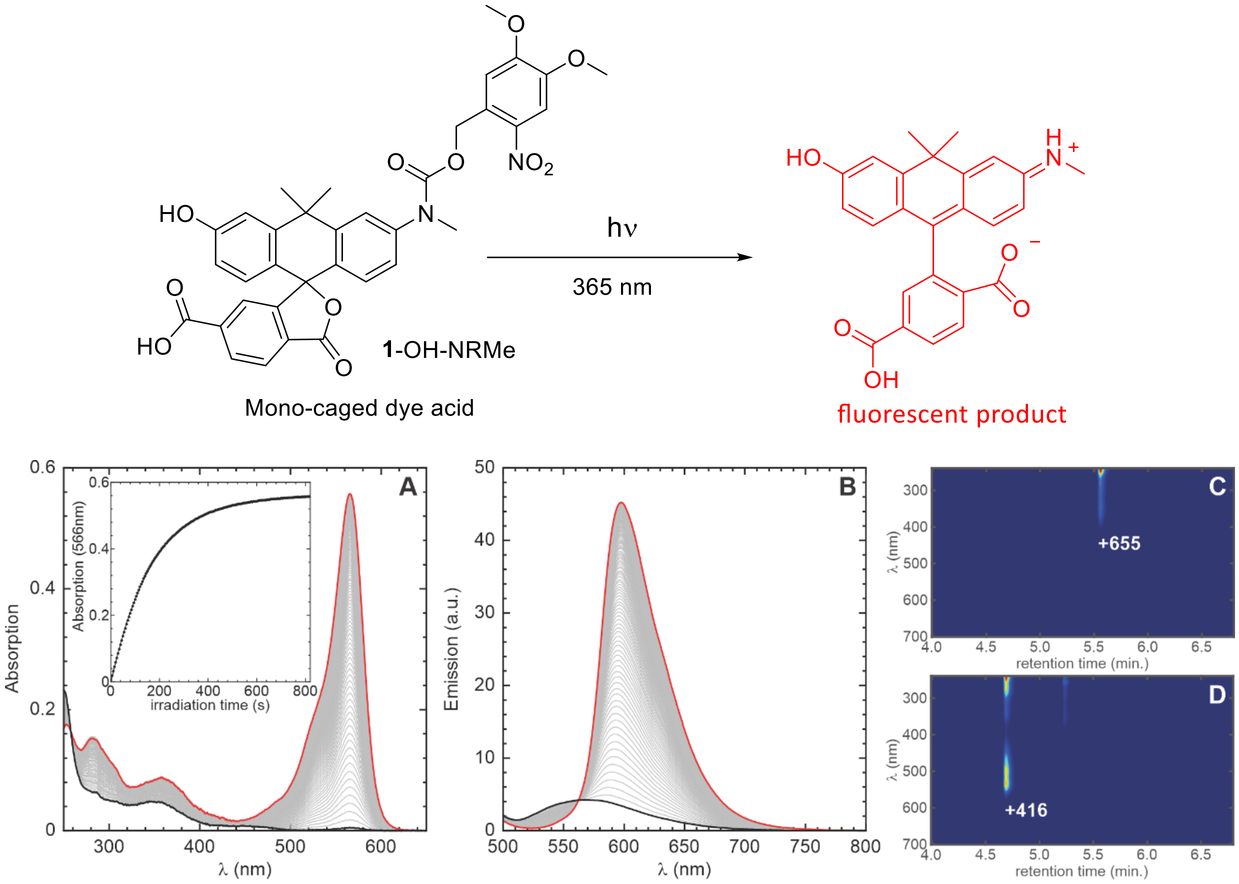

Figure S1. Photolysis of mono-caged dye **1**-OH-NRMe in aq. buffer:DMSO (80:20) solution. Absorption (A) and emission (B) spectra at different irradiation times. The spectra before irradiation and after complete uncaging are plotted with black and red lines, respectively. The inset in (A) displays the transient at the absorption maximum of the fluorescent product (566 nm). LCMS 2D maps "retention time *vs*. absorption spectra" (C) and (D).

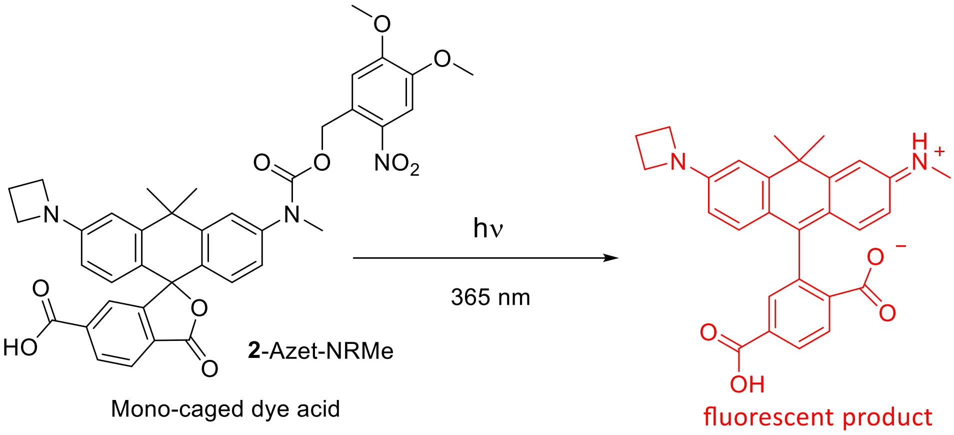

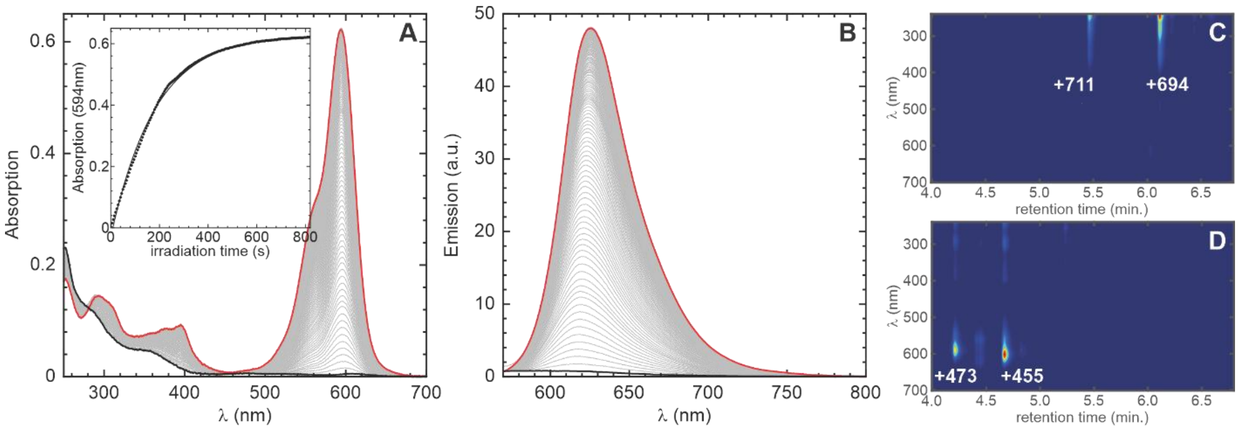

Figure S2. Photolysis of mono-caged dye **2**-Azet-NRMe in aq. buffer:DMSO (80:20) solution. Absorption (A) and emission (B) spectra at different irradiation times. The spectra before irradiation and after complete uncaging are plotted with black and red lines, respectively. The inset in (A) displays the transient at the absorption maximum of the fluorescent product (594 nm). LCMS 2D maps “retention time *vs*. absorption spectra” (C) and (D).

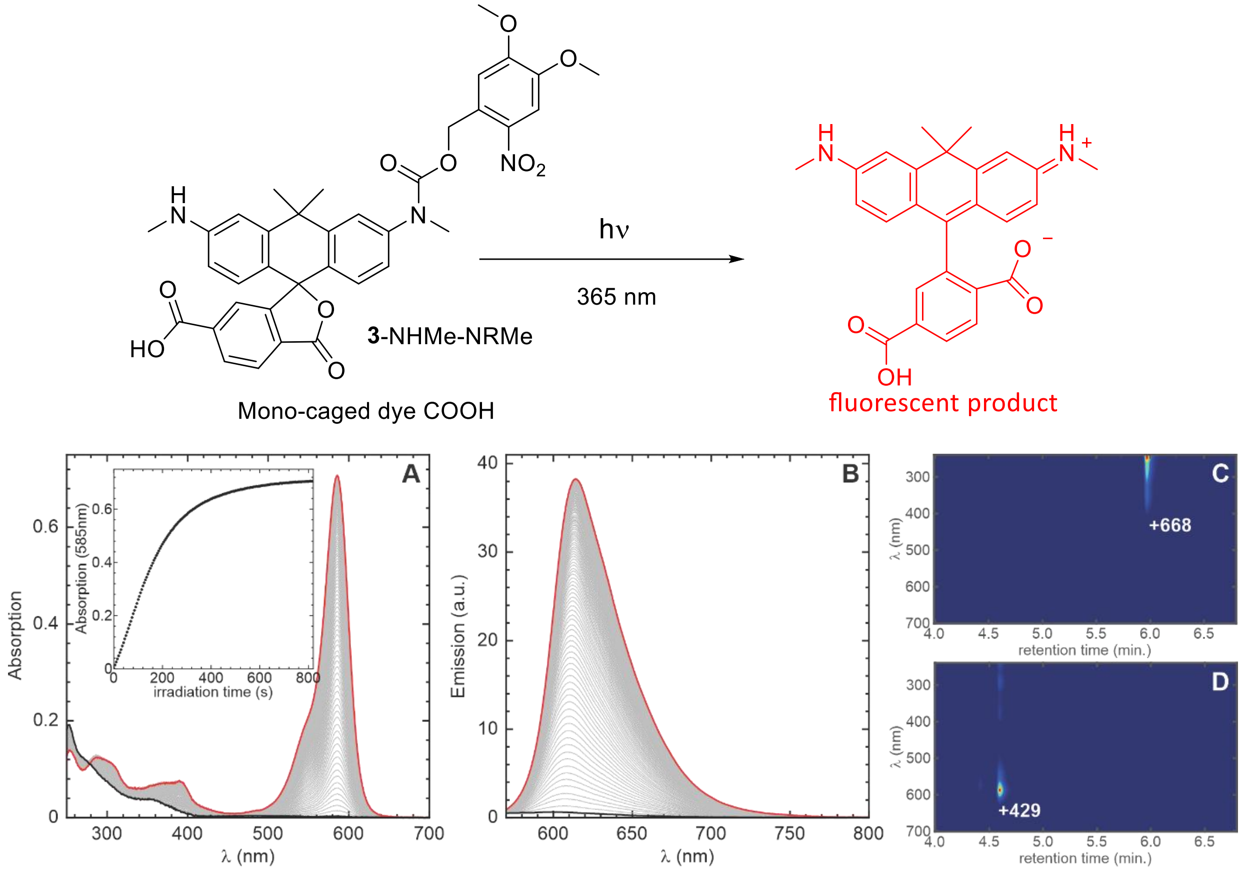

Figure S3. Activation of the mono-caged free acid of **3**-NHMe-NRMe in aq. buffer:DMSO (80:20) solution. Absorption (A) and emission (B) spectra at different irradiation times. The spectra before irradiation and after complete uncaging are plotted with black and red lines, respectively. The inset in (A) displays the transient at the absorption maximum of the fluorescent product (566 nm). LCMS 2D maps “retention time *vs*. absorption spectra” (C) and (D).

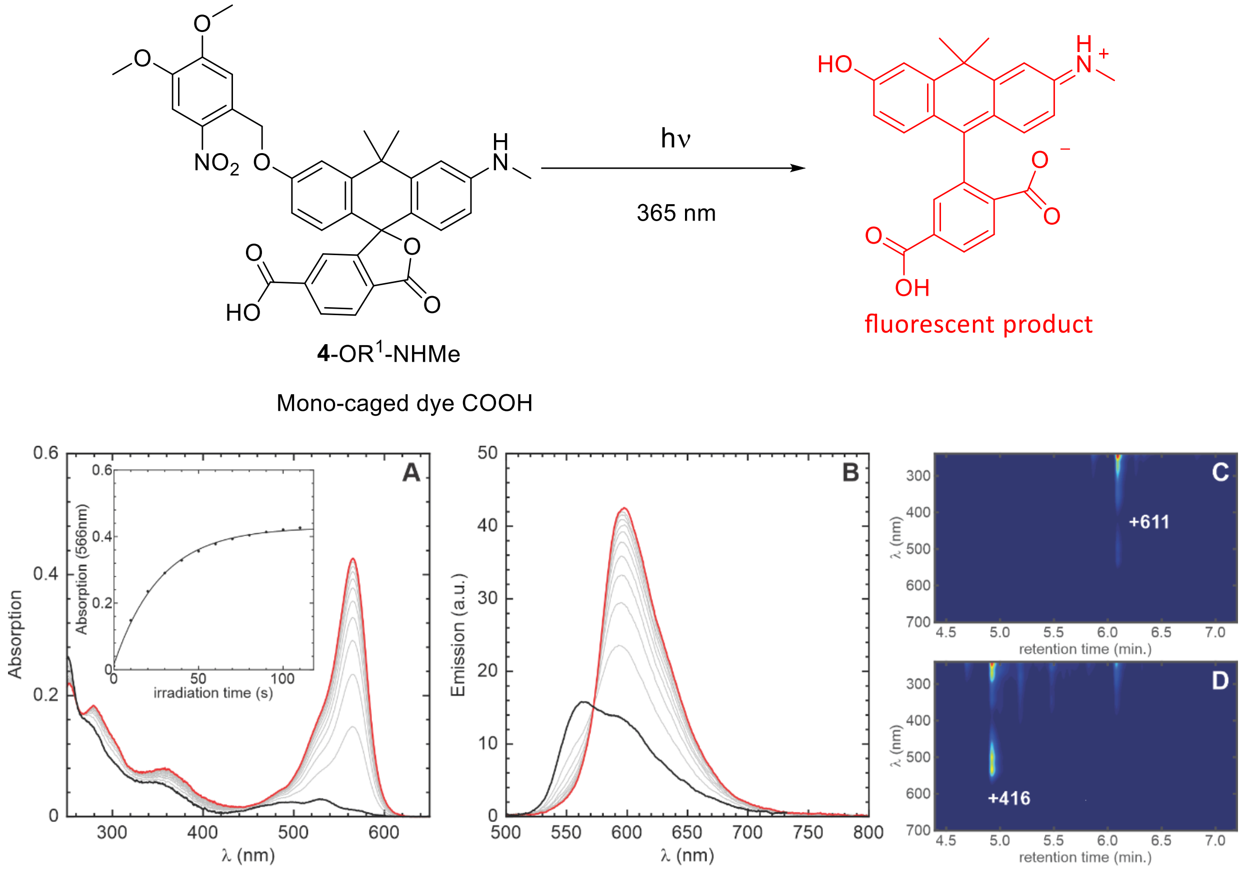

Figure S4. Activation of the mono-caged free acid of **4**-OR[1]Me-NRMe in aq. buffer:DMSO (80:20) solution. Absorption (A) and emission (B) spectra at different irradiation times. The spectra before irradiation and after complete uncaging are plotted with black and red lines, respectively. The inset in (A) displays the transient at the absorption maximum of the fluorescent product (566 nm). LCMS 2D maps "retention time *vs*. absorption spectra" (C) and (D).

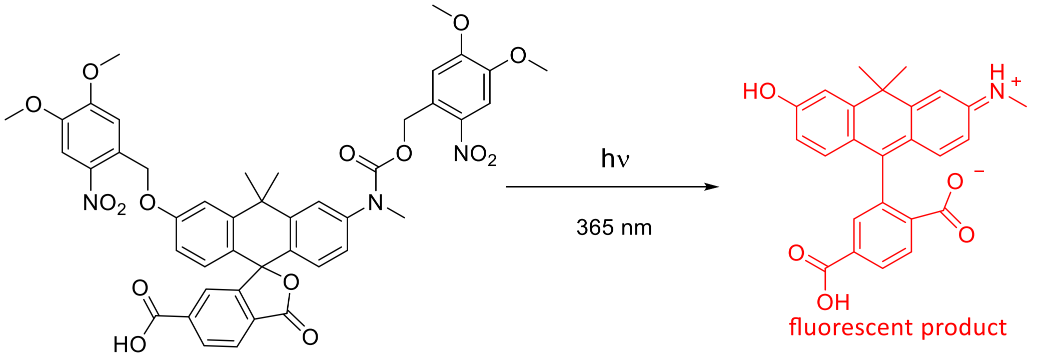

5-OR[1]-NRMe
Bis-caged dye COOH

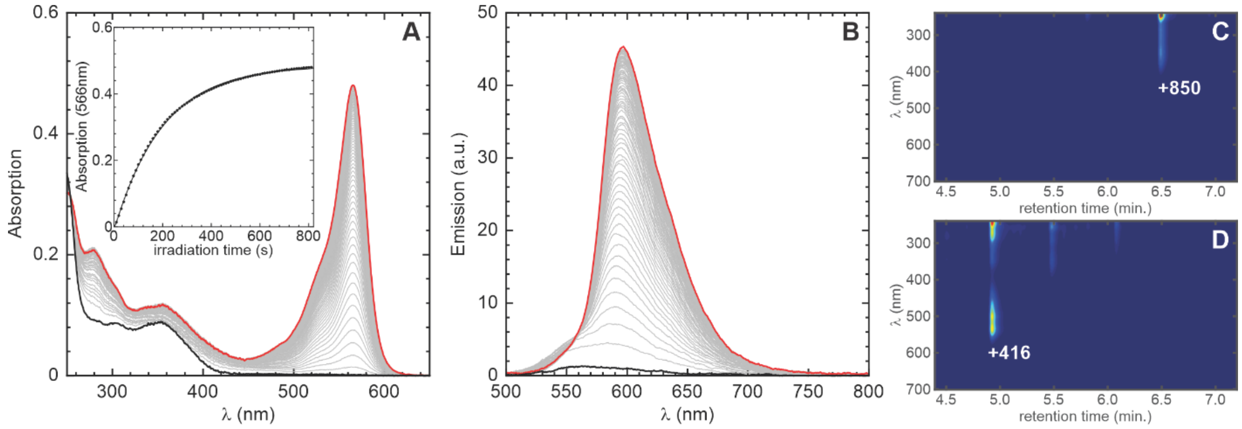

Figure S5. Activation of the bis-caged free acid of **5**-OR[1]Me-NRMe in aq. buffer:DMSO (80:20) solution. Absorption (A) and emission (B) spectra at different irradiation times. The spectra before irradiation and after complete uncaging are plotted with black and red lines, respectively. The inset in (A) displays the transient at the absorption maximum of the fluorescent product (566 nm). LCMS 2D maps "retention time *vs*. absorption spectra" (C) and (D).

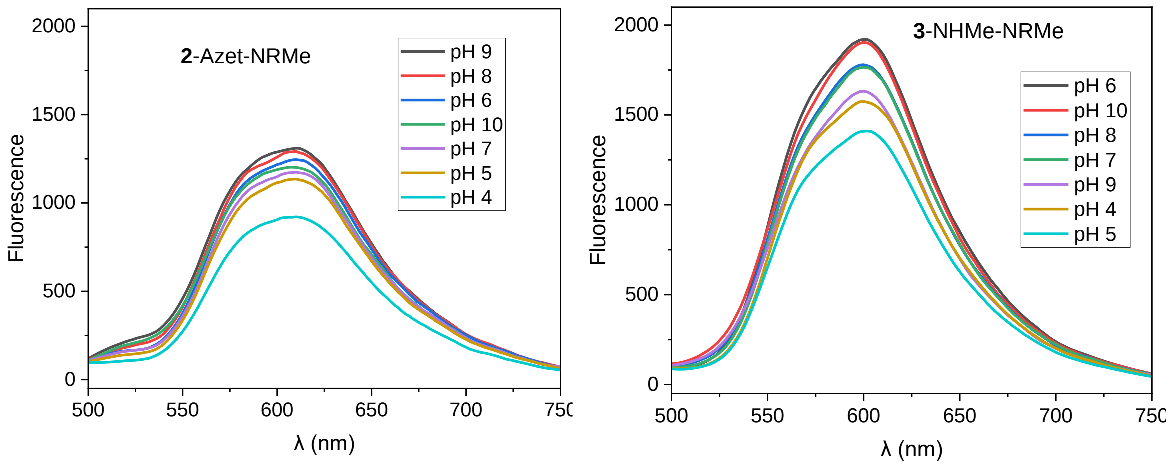

Figure S6. Fluorescence spectra of mono-caged dyes **2**-Azet-NRMe and **3**-NHMe-NRMe at different pH values.

HaloTag Staining and Sample Preparation

U2OS cells with a Vimentin-HaloTag construct were grown for 24-72 h on glass coverslips in Dulbecco's modified Eagle's medium (Gibco, supplemented with 10% fetal bovine serum, 2% GlutaMAX and 1% penicillin/streptomycin). Staining of the HaloTag constructs were performed for 1 h with 500 nM of the probe in the cell culture media. Cells were washed 2-3 times in culture media, for 15 min each time, and mounted in FluoroBrite DMEM (Gibco, supplemented with 10% fetal bovine serum, 2% GlutaMAX and 1% penicillin/streptomycin) for live cell imaging.

For SMLM and MINFLUX imaging, samples were additionally rinsed with warm PBS (pH 7.4) and the fixation was carried out with a 4% formaldehyde solution in PBS at room temperature for 20 min. Samples were then washed 3 times (5 min each time) with PBS and mounted in PBS (or Mowiol for Figure S10) without additives. For MINFLUX imaging, samples were additionally incubated with 150 nm gold beads (BBI Solutions, EM. GC150) for 5 min, washed with PBS (3x5 min), and mounted in PBS without additives.

Confocal Imaging

Imaging was performed on an Abberior Confocal microscope (Expert Line, Abberior Instruments GmbH) at the Optical Microscopy Facility of the Max Planck Institute for Medical Research. Images were acquired with 561 nm excitation (18 µW) and detection in the 571-691 nm range (**8**-H-HT, **15** and **16**) or in the 571-711 nm range (**11** and **14**). Activation was performed with the 405 nm laser line (12-18 µW). A pixel size of 70 nm and an integration time of 10-20 µs was used. Imaging and image processing was done with ImSpector software (v. 16.3.13367; Abberior Instruments GmbH, Göttingen, Germany), and all images are displayed as raw data.

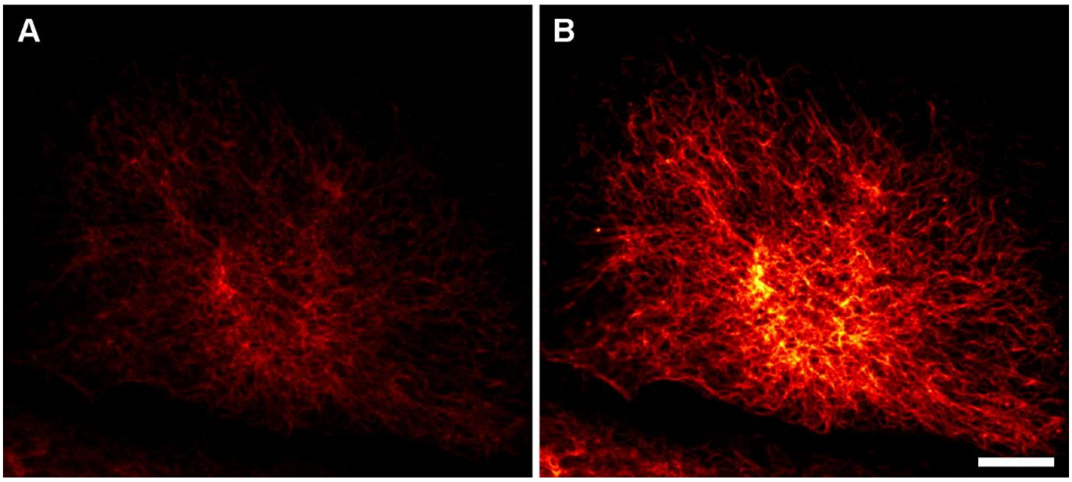

Figure S7. Confocal image of live U2OS cells expressing Vimentin-HaloTag construct labeled with compound **11**. before (A) and after (B) activation with 405 nm light. Scale bar: 10 µm.

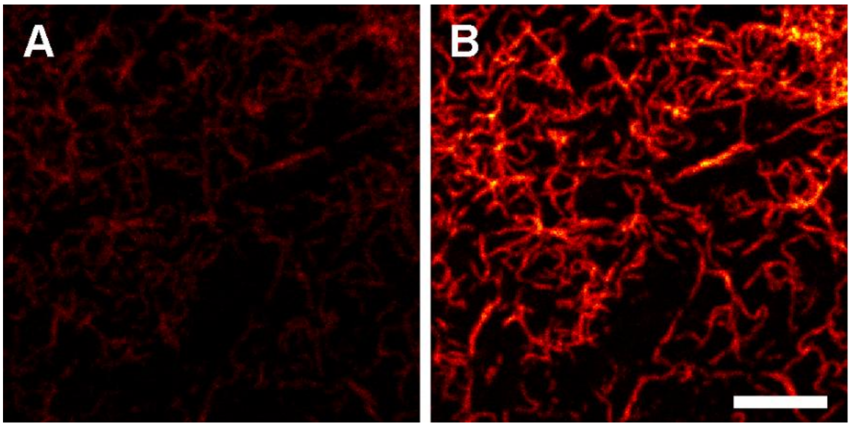

Figure S8. Confocal image of live U2OS cells expressing Vimentin-HaloTag construct labeled with compound **14**. before (A) and after (B) activation with 405 nm light. Scale bar: 5 µm.

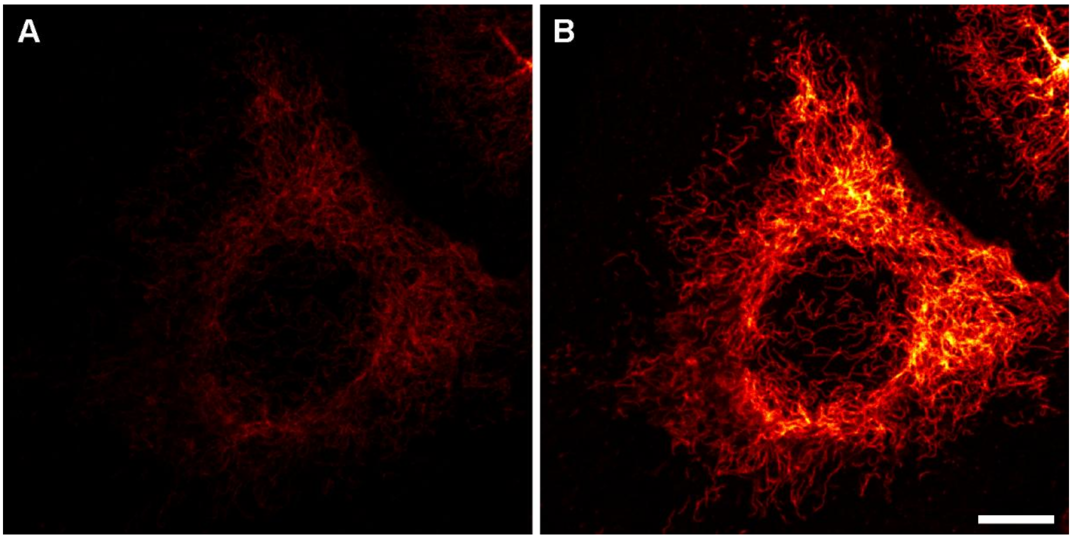

Figure S9. Confocal image of live U2OS cells expressing Vimentin-HaloTag construct labeled with compound **15**. before (A) and after (B) activation with 405 nm light. Scale bar: 10 µm.

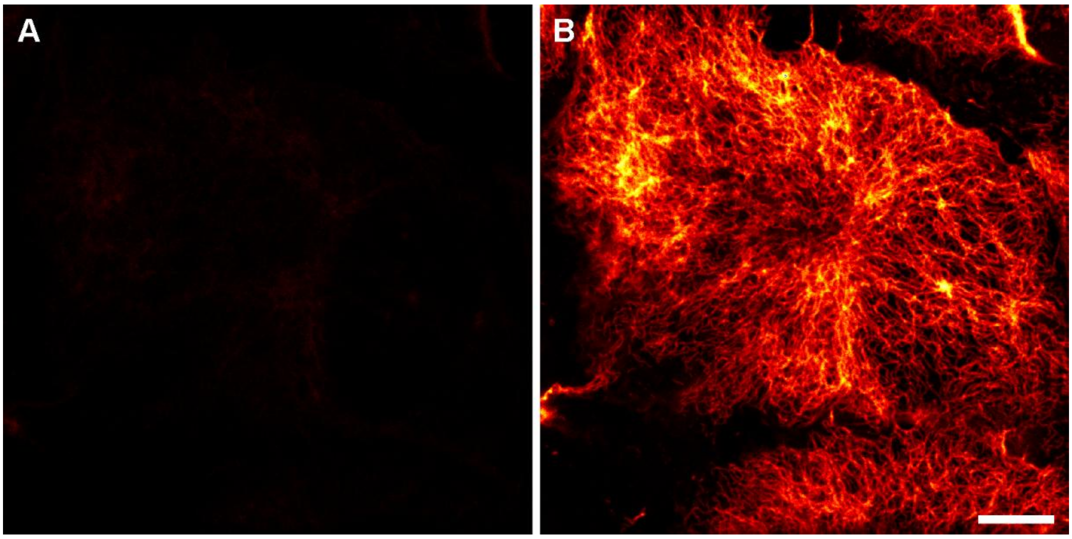

Figure S10. Confocal image of live U2OS cells expressing Vimentin-HaloTag construct labeled with compound **16**. before (A) and (B) after activation with 405 nm light. Scale bar: 10 µm.

Cell viability test

U2OS cells with a Vimentin-HaloTag construct were seeded in Costar 96 well plate (Corning 3603) at a density of 5*10^3 cells/well (100 µL/well) in cell medium (supplemented Dulbecco's modified Eagle's medium) and incubated for 24h at 37°C. The medium was removed and replaced with 100 µL/well of supplemented FluoroBrite DMEM containing either 500 nM of the corresponding dye in DMSO (0.1% final DMSO), 0.1 % DMSO (Control) or only cell medium (Blank). Then the samples were incubated for 2 h at 37°C and washed afterwards 2 times for 10 min each with fresh supplemented FluoroBrite DMEM. Half of the samples were then irradiated in a BioTek Cytation 5 Cell Imaging Multimode Reader (Agilent, CYT5PW-SN) with a DAPI filter (LED 365 nm; Ex. Filter 377/50 nm; Em. Filter 447/60 nm). The dye activation was corroborated by imaging with a Texas Red filter (LED 590 nm; Ex. Filter 586/15 nm; Em. Filter 647/57 nm). After that, all samples were incubated for another 24 h at 37°C and then analyzed with ReadyProbes Cell Viability Imaging Kit Blue/Green (Invitrogen™, R37609) according to the protocol of the manufacturer. For that, 2 drops of each reagent were added per 1 mL of supplemented FluoroBrite DMEM and incubated for 15 min at 37°C. Cells were then imaged, and DAPI and GFP (LED 465 nm; Ex. Filter 469/35 nm; Em. Filter 525/39 nm) signals counted using the Gen5 Software to obtain total cell count (DAPI channel) and dead cell count (GFP channel); the viability assessed as the percentage of live cells (Figure S11). Each condition (irradiated/not irradiated) and dye was measured by triplicates (3 different wells per condition). The irradiation of the dye was performed in the whole well (4xROIs), to evaluate the effect in the whole population, but the cell count was performed only in a ROI in the center of the well, to avoid background effects in the border of the well (see Figure S11-B).

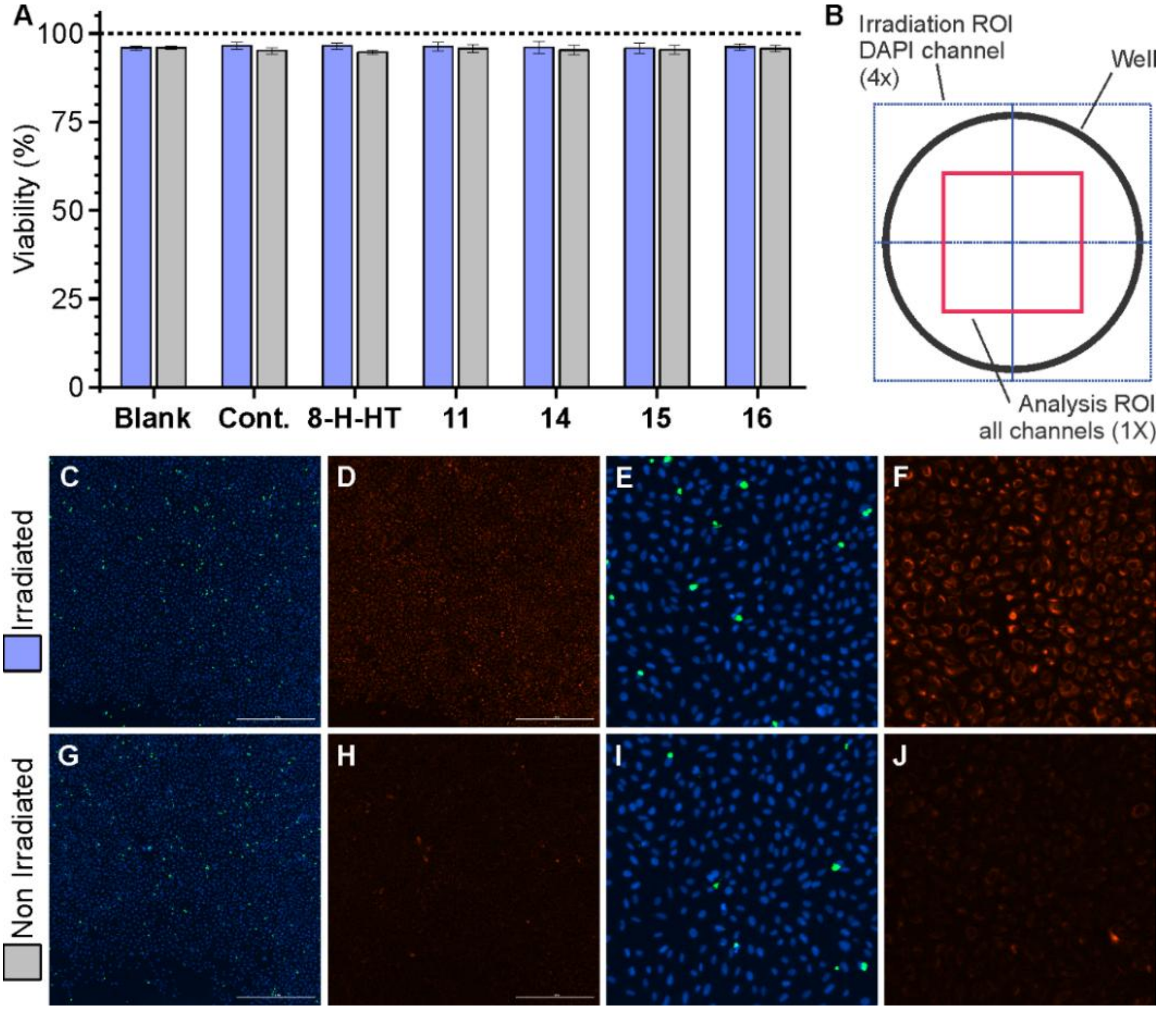

Figure S11. (A) Viability assay of U2OS cells expressing Vimentin-HaloTag construct labeled with the indicated compounds (2 h, 500 nM dye, DMSO 0.1%), the Blank (no treatment), and the Control (only DMSO 0.1%). Irradiated cells are shown as blue and non-irradiated as grey bars. (B) Schematic representation of the irradiated and analyzed area on each well. (C-J) Images recorded in two wells treated with compound **14** (where the dye was irradiated C-F or not irradiated G-J) in DAPI/GFP channel (C, E, G, I) and in the Texas Red channel (D, F, H, J). Images in E, F, I, and J are 4x zoom of the selected ROIs from C, D, G, H, respectively.

SMLM Imaging

Images were acquired using an ONI Nanoimager V3 (Oxford Nanoimaging, Oxford, UK) in TIRF configuration. Excitation was performed with a 560 nm laser (160 mW), and activation with 405 nm laser (variable power adjusted manually to sustain sparse single-molecule activation). Detection was filtered to the 580 – 620 nm range. Images were analysed and processed using the ONI Nanoimager™ Software (Version: 1.19.7.20230409223555 - 28f00b5), and post-processed using custom-built MatLab (version R2007a) routines. Data was drift-corrected and filtered (sigma = 90-150 nm). Final images were produced with an averaged shifted histogram method,[2] with a pixel-size of 10 nm.

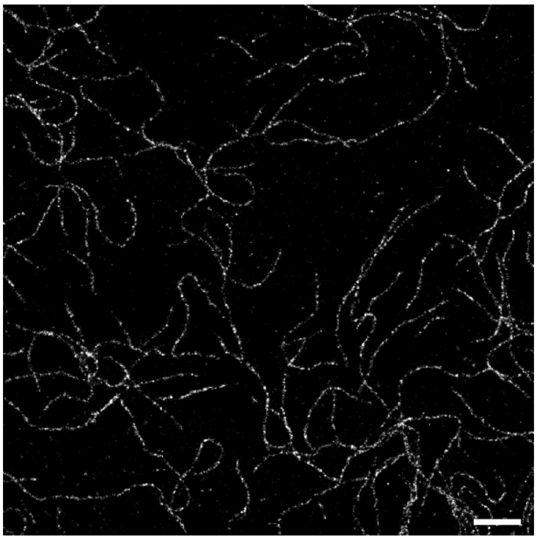

Figure S12. PALM image of fix U2OS cells expressing Vimentin-HaloTag construct labeled live with compound **8**-H-HT (500 nM/1h), mounted in Mowiol. Scale bar: 1 µm.

MINFLUX Imaging

Images were acquired on an Abberior Instruments MINFLUX microscope, equipped with a 488 nm excitation line (Only confocal), 561 and 640 nm excitation (Confocal and MINFLUX) lines and a 405 nm activation line, filter-based detection channels with avalanche photo diodes (GFP: 500-550 nm, Cy3: 580-630 nm, Cy5 "near": 650-685 nm, Cy5 "far": 685-720 nm), and a camera-based sample's stabilization system with a 975 nm laser. Imaging of **8**-H-HT was performed with the 560 nm MINFLUX line, 405 nm activation, and detection was performed with the signal combined from the Cy3 and the Cy5 "near" detection channels. Excitation power was set to 4% of the maximum power on the first iteration corresponding approximately to 30 µW. The power of the 405 nm activation line was set to 0% gradually increased manually up to 100% (20µW) to sustain the frequency of detected events. Stabilization was locked on gold beads in automatically selected areas by the microscopy software. A modified imaging sequence with 100 photons per iteration and a background threshold of 3 kHz was used for imaging.

Images were processed with a custom-built MatLab routine with a density-based clustering algorithm dbscan (epsilon = 4 nm, minPts = 3) followed by filtering for molecules with a minimum of 2 localizations in a radius of 5 nm. The resulting image was rendered as normalized Gaussians with a fixed sigma of 3 nm and a pixel size of 1 nm, and with a nonlinear colour map in square roots for better visualization (gamma correction with A=1 and γ= 0.5).

General experimental information and synthesis

NMR spectra were measured on an Agilent 400-MR spectrometer at 400 MHz (^{1}H), and 101 MHz (^{13}C). ^{13}C NMR spectra (126 MHz) were also acquired with a Bruker Avance III HD 500 MHz (BBO Prodigy probe) instrument at the Institute of Organic and Biomolecular Chemistry (Georg-August-Universität Göttingen).

Chemical shifts (δ) are reported in ppm. All ^{1}H-NMR spectra are referenced to tetramethylsilane (TMS; δ = 0 ppm) using the signals of added TMS (0.03% v/v), or the residual signals of $CHCl_3$ (7.26 ppm) in $CDCl_3$, CHD_2OD (3.31 ppm) in CD_3OD, CHD_2CN (1.94 ppm) in CD_3CN, DMSO-d_5 (2.50 ppm) in DMSO-d_6. ^{13}C NMR spectra are referenced to TMS (δ = 0 ppm) using the signals of added TMS (0.03% v/v), or the solvent: $CDCl_3$ (77.16 ppm), CD_3OD (49.00 ppm), CD_3CN (1.32 ppm), DMSO-d_6 (39.52 ppm). Multiplicities of signals are described as follows: s = singlet, d = doublet, t = triplet, q = quartet, p = pentet, m = multiplet or overlap of non-equivalent resonances; brs. = broad singlet. Coupling constants (J) are given in Hz.

ESI-MS were recorded on a Varian 500-MS spectrometer (Agilent). ESI-HRMS were recorded on a MICROTOF spectrometer (Bruker) equipped with ESI ion source (Apollo) and direct injector with LC autosampler Agilent RR 1200 at the Institute of Organic and Biomolecular Chemistry (Georg-August-Universität Göttingen).

High-performance liquid chromatography (HPLC): analytical HPLC was performed on a Knauer Azura system with a binary P6.1L pump, UV diode array detector DAD 6.1L, an injection valve with a 20 µL loop and two electrical switching valves V2.1S with 6-port valve head. Analytical columns: Knauer Eurospher II 100-5 C18, 5 µm, 150×4 mm or Interchim Uptisphere Strategy C18-HQ, 10 µm, 250×4.6 mm, flow rate 1.2 mL/min, unless stated otherwise.

Preparative HPLC on reversed phase was performed on a puriFlash 4250 2X HPLC/Flash hybrid system (Interchim) with an injection loop (2 mL or 5 mL volume), a 200-800 nm UV-Vis detector and an integrated ELSD detector. Preparative columns: Interchim Eurosphere II C18H 5 µm, 250×16 mm, flow rate 10 mL/min and Eurosphere II C18, 5 µm, 250×20 mm, flow rate 20 mL/min, unless specified otherwise.

Automated flash column chromatography was performed on regular SiO_2 with an Isolera One system (Biotage AG, Sweden) using commercially available cartridges (RediSep Rf series from Teledyne ISCO, Puriflash Silica HP 30 µm series from Interchim, and Biotage Sfaer silica HC) and the solvent gradient indicated. Analytical TLC was performed on MERCK ready-to-use plates with silica gel 60 (F254).

Heating of the reaction mixtures was performed in oil baths, with temperatures indicated as reaction temperatures. All reagents and solvents were purchased from commercial sources and used without further purification. The starting materials - aryl-ditriflate (**6**) and carbamate **7** - were prepared according to the published method.[3] The Halo-Tag amine (O_2) ligand was prepared according to literature[4].

Scheme S1

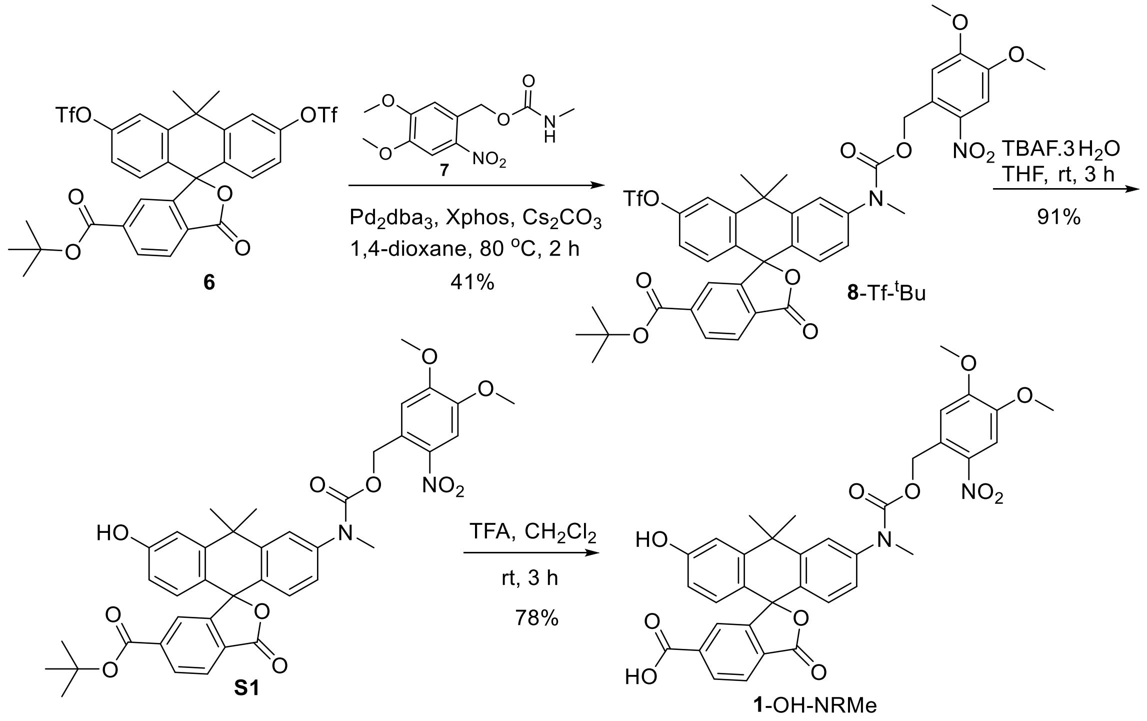

Scheme S1. Synthesis of mono-caged rhodol acid, **1**-OH-NRMe.

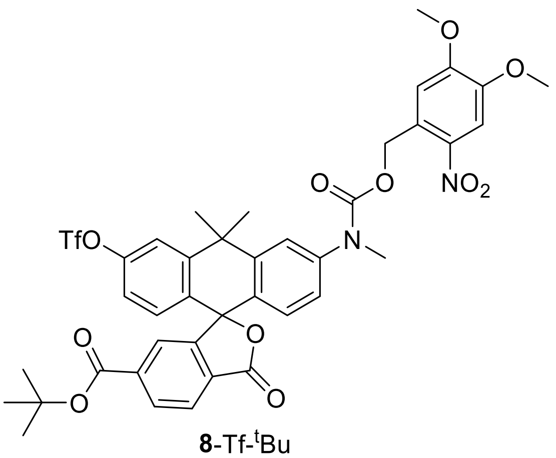

8-Tf-^{t}Bu

Compound 8-Tf-^{t}Bu: Compounds **6** (100 mg, 0.14 mmol), **7** (38 mg, 0.14 mmol, 1 eq.), $Pd_2(dba)_3$ (6.3 mg, 7 µmol, 5 mol%), XPhos (10 mg, 21 µmol, 15 mol%) and Cs_2CO_3 (63 mg, 200 µmol, 1.4 eq) were combined in a microwave vial and flushed with argon. Degassed dry 1,4-dioxane (3 mL) was added, the

vial was sealed and heated at 80 °C for 2 h in an oil bath. Upon cooling, the reaction mixture was filtered through Celite, filter-cake washed with ethyl acetate, and the filtrate evaporated. The residue was purified by means of flash column chromatography on silica gel (0 – 80% ethyl acetate in *n*-hexane) to get compound **8**-Tf-^{t}Bu (48 mg, yield 41%) as off-white solid (contains ~10% of the side product with the same R_f value).

^{1}H NMR (400 MHz, $CDCl_3$) δ 8.23 (dd, *J* = 8.0, 1.3 Hz, 1H), 8.09 (dd, *J* = 8.0, 0.7 Hz, 1H), 7.69 (s, 1H), 7.65 – 7.59 (m, 2H), 7.55 (d, *J* = 2.6 Hz, 1H), 7.10 (ddd, *J* = 11.4, 8.7, 2.4 Hz, 2H), 6.87 (d, *J* = 8.8 Hz, 1H), 6.79 (s, 1H), 6.76 (d, *J* = 8.5 Hz, 1H), 5.57 (s, 2H), 3.94 (s, 3H), 3.76 (s, 3H), 3.39 (s, 3H), 1.87 (s, 3H), 1.75 (s, 3H), 1.56 (s, 9H). ^{13}C NMR (126 MHz, $CDCl_3$) δ 168.9, 163.9, 154.6, 154.2, 153.4, 151.4, 150.1, 148.2, 147.7, 145.0, 144.2, 138.5, 131.2, 130.9, 130.5, 130.1, 128.8, 128.4, 127.2, 126.8, 125.5, 124.6, 120.1, 120.0, 119.7, 117.4, 110.3, 108.2, 84.7, 82.8, 64.9, 56.4, 56.3, 38.6, 37.3, 34.6, 33.2, 28.0.

HRMS (ESI) calcd for $C_{40}H_{37}F_3N_2O_{13}S$ $[M+Na]^+$ 865.1861, found 865.1862.

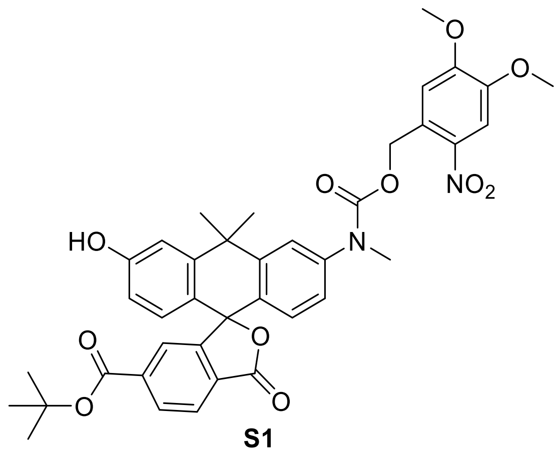

S1

Compound S1: TBAF*3H_2O (16 mg) was added to a solution of **8**-Tf-^{t}Bu (14 mg, 17 µmol) in THF (2 mL) at room temperature[5]. The resulting solution was stirred at room temperature for 3 h. THF was evaporated and reaction mixture was acidified with 1M HCl, extracted with EtOAc (4 x), dried (Na_2SO_4) and concentrated vacuo. The residue was purified by flash column chromatography on silica gel (0 – 60% ethyl acetate in *n*-hexane) to get compound **S1** (10.8 mg, yield 91%) as a pink solid.

^{1}H NMR (400 MHz, CD_3CN) δ 8.22 (dd, *J* = 8.0, 1.3 Hz, 1H), 8.07 (dd, *J* = 8.0, 0.8 Hz, 1H), 7.74 (d, *J* = 2.2 Hz, 1H), 7.69 (s, 1H), 7.52 (dd, *J* = 1.3, 0.8 Hz, 1H), 7.22 – 7.14 (m, 2H), 6.88 (brs, 1H), 6.80 (d, *J* = 8.0,, 1H), 6.72 – 6.59 (m, 2H), 5.47 (s, 2H), 3.88 (s, 3H), 3.67 (brs, 3H), 3.36 (s, 3H), 1.80 (s, 3H), 1.69 (s, 3H), 1.51 (s, 9H). ^{13}C NMR (151 MHz, CD_3CN) δ 170.1, 164.9, 158.9, 156.1, 155.4, 154.7, 149.1, 147.8, 147.1, 145.2, 140.6, 139.3, 131.3, 130.3, 130.1, 129.8, 129.1, 128.3, 126.2, 125.6, 125.4, 124.8, 122.8, 116.01, 113.8, 111.1, 109.2, 86.8, 83.3, 65.2, 56.9, 56.9, 39.1, 38.2, 34.8, 33.4, 28.1. HRMS (ESI) calcd for $C_{39}H_{38}N_2O_{11}$ $[M+Na]^+$ 733.2368, found 733.2350.

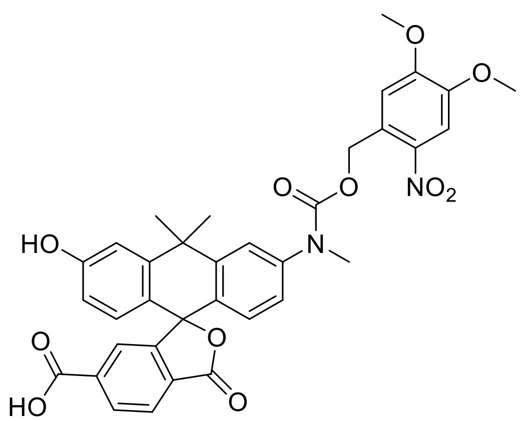

1-OH-NRMe

Compound 1-OH-NRMe: Trifluoroacetic acid (0.1 mL) was added to a solution of **S1** (2.5 mg, 3.5 μmol) in CH_2Cl_2 (0.8 mL) at room temperature. The resulting solution was stirred at room temperature for 3 h. The reaction mixture was co-distilled in vacuum with toluene (2 x) and then with acetonitrile. The residue was separated by means of prep. HPLC on reverse phase (C18) using water and acetonitrile (0.1% TFA); 70/30 to 0/100, in 25 min to get compound **1**-OH-NRMe (1.8 mg, yield 78%) as a brownish pink solid.

^{1}H NMR (400 MHz, CD_3OD) δ 8.29 (dd, *J* = 8.0, 1.3 Hz, 1H), 8.11 (dd, *J* = 8.0, 0.7 Hz, 1H), 7.76 (d, *J* = 2.2 Hz, 1H), 7.71 (s, 1H), 7.59 (dd, *J* = 1.3, 0.8 Hz, 1H), 7.20 (dd, *J* = 8.5, 2.2 Hz, 1H), 7.13 (d, *J* = 2.4 Hz, 1H), 6.83 (brs, 1H), 6.80 (d, *J* = 8.5 Hz, 1H), 6.65 (dd, *J* = 8.7, 2.4 Hz, 1H), 6.59 (d, *J* = 8.5 Hz, 1H), 5.50 (s, 2H), 3.88 (s, 3H), 3.63 (brs, 3H), 3.37 (s, 3H), 1.82 (s, 3H), 1.71 (s, 3H). HRMS (ESI) calcd for $C_{35}H_{30}N_2O_{11}$ $[M+Na]^+$ 677.1742, found 677.1742.

Scheme S2

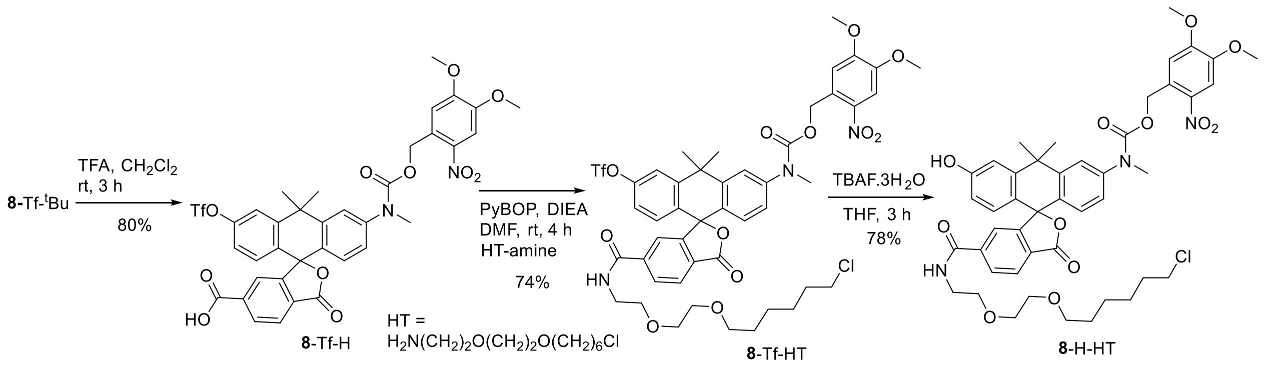

Scheme S2. Synthesis of mono-caged rhodol HaloTag derivative, **8**-H-HT

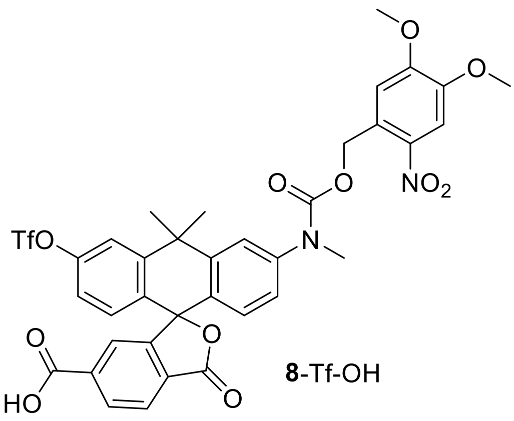

Compound 8-Tf-OH: Trifluoroacetic acid (0.3 mL) was added to a solution of **8**-Tf-^{t}Bu (5.8 mg, 7 µmol) in CH_2Cl_2 (2 mL) at room temperature. The resulting solution was stirred at room temperature for 1 h. The reaction mixture was concentrated and co-distilled with acetonitrile in vacuum to get crude compound **8**-Tf-OH. It was purified by means of reverse phase HPLC (Interchim) using water and acetonitrile (0.1% TFA); 70/30 to 0/100, in 25 min to obtain compound **8**-Tf-OH (4.3 mg, yield 80%) as an off-white solid.

^{1}H NMR (400 MHz, CD_3CN) δ 8.29 (dd, *J* = 8.0, 1.3 Hz, 1H), 8.14 (dd, *J* = 8.0, 0.8 Hz, 1H), 7.80 (d, *J* = 2.6 Hz, 1H), 7.75 (d, *J* = 2.2 Hz, 1H), 7.68 (s, 1H), 7.60 (dd, *J* = 1.3, 0.8 Hz, 1H), 7.23–7.20 (m 2H), 7.00 (d, *J* = 8.8 Hz, 1H), 6.90 (brs, 1H), 6.83 (d, *J* = 8.8 Hz, 1H), 5.47 (s, 2H), 3.88 (s, 3H), 3.69 (s, 3H), 3.36 (s, 3H), 1.84 (s, 3H), 1.72 (s, 3H). ^{13}C NMR (126 MHz, CD_3CN) δ 169.6, 166.2, 155.4, 155.4, 154.6, 151.2, 149.2, 149.1, 146.0, 145.5, 140.7, 137.9, 132.3, 132.1, 131.3, 130.1, 129.2, 128.6, 128.1, 126.8, 126.0, 125.4, 123.54, 121.4, 121.2, 121.0, 111.2, 109.2, 85.5, 65.3, 56.9, 56.9, 39.5, 38.2, 34.6, 33.4.

HRMS (ESI) calcd for $C_{36}H_{29}F_3N_2O_{13}S_1$ $[M+Na]^+$ 809.1235, found 809.1247.

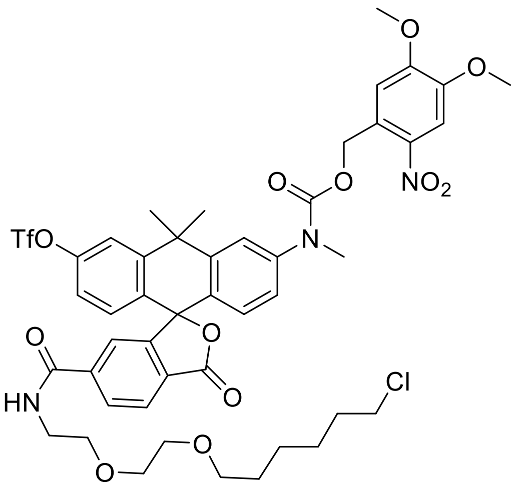

Compound 8-Tf-HT: Compound **8**-Tf-OH (4 mg, 5 µmol, 1 eq), HaloTag-Amine (1.7 mg, 6.1 µmol, 1.5 eq) and *N,N*-diisopropylethylamine (6 µL, 7.6 µmol, 6 eq) were dissoleved in DMF (1 mL), and then

PyBOP (3.2 mg, 6.1 μmol, 1.2 eq) was added at rt. The reaction mixture was stirred at rt for 4 h, concentrated under vacuum, and the residue purified by means of reversed phase HPLC (Interchim) [A:B 30:70 → 0:100 over 25 min, A – acetonitrile (1% TFA), B – water (1% TFA)] to get compound **8**-Tf-HT (3.7 mg, yield: 74%) as an off-white solid.

^{1}H NMR (400 MHz, CD_3CN) δ 8.12 (dd, *J* = 8.0, 0.8 Hz, 1H), 8.07 (dd, *J* = 8.0, 1.4 Hz, 1H), 7.80 (d, *J* = 2.6 Hz, 1H), 7.75 (d, *J* = 2.2 Hz, 1H), 7.69 (s, 1H), 7.38 (dd, *J* = 1.4, 0.8 Hz, 1H), 7.24 – 7.20 (m, 2H), 7.00 (dd, *J* = 8.9, 0.3 Hz, 1H), 6.91 (s, 1H), 6.84 (dd, *J* = 8.9, 0.3 Hz, 1H), 5.47 (s, 2H), 3.88 (s, 3H), 3.69 (s, 3H), 3.59 – 3.49 (m, 6H), 3.48 – 3.41 (m, 4H), 3.36 (s, 3H), 3.34 – 3.29 (m, 2H), 1.85 (s, 3H), 1.73 (s, 3H), 1.74 – 1.67 (m, 2H), 1.48 – 1.39 (m, 2H), 1.37 – 1.26 (m, 4H).

HRMS (ESI) calcd for $C_{46}H_{49}ClF_3N_3O_{14}S$ $[M+Na]^+$ 1014.2468, found 1014.2472.

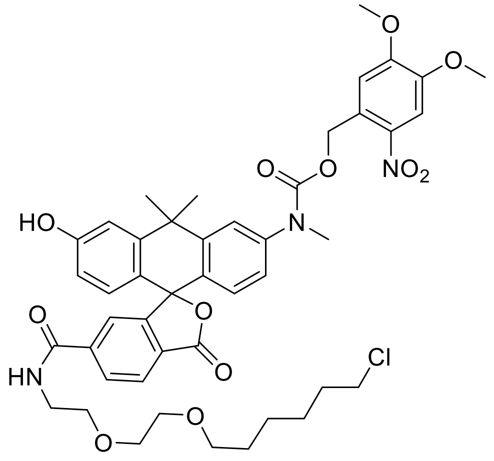

8-H-HT

Compound 8-H-HT: TBAF*3H_2O (3.8 mg, 12 μmol, 3 eq) was added to a solution of compound **8**-Tf-HT (4 mg, 4 μmol) in THF (1 mL) at room temperature. The resulting solution was stirred at room temperature for 3 h. The reaction mixture was acidified with 1.0 M aq HCl and concentrated in vacuum. The residue was separated by means of reversed phase HPLC (Interchim) [A:B 30:70 → 0:100 over 25 min, A – acetonitrile (0.1% TFA), B – water (0.1% TFA)] to get compound **8**-H-HT (2.5 mg, yield 78%) as a magenta-colored solid.

^{1}H NMR (400 MHz, CD_3CN) δ 8.10 – 7.99 (m, 2H), 7.73 (d, *J* = 2.2 Hz, 1H), 7.69 (s, 1H), 7.35 (dd, *J* = 1.4, 0.8 Hz, 1H), 7.21 (brs, 1H), 7.19 (dd, *J* = 8.5, 2.3 Hz, 1H), 7.16 (dd, *J* = 2.3, 0.6 Hz, 1H), 6.89 (brs, 1H), 6.79 (dd, *J* = 8.5, 0.3 Hz, 1H), 6.74 – 6.60 (m, 2H), 5.47 (s, 2H), 3.88 (s, 3H), 3.67 (brs, 3H), 3.59 – 3.49 (m, 6H), 3.48 – 3.41 (m, 4H), 3.36 (s, 3H), 3.31 (t, *J* = 6.5 Hz, 2H), 1.81 (s, 3H), 1.75 – 1.63 (m, 2H), 1.69 (s, 3H), 1.48 – 1.25 (m, 6H).

HRMS (ESI) calcd for $C_{45}H_{50}ClN_3O_{12}$ $[M+Na]^+$ 882.2975, found 882.2970.

Scheme S3

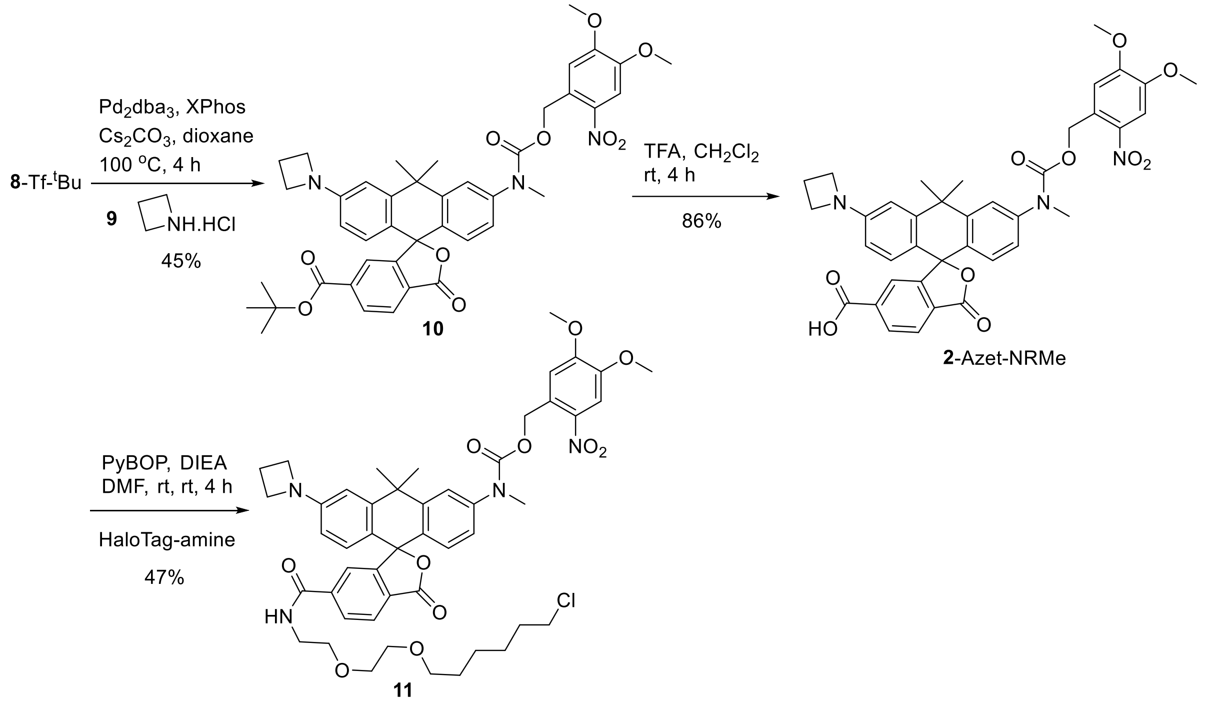

Scheme S3. Synthesis of mono-caged rhodol acid, **2**-Azet-NRMe and its HaloTag derivative, **11**

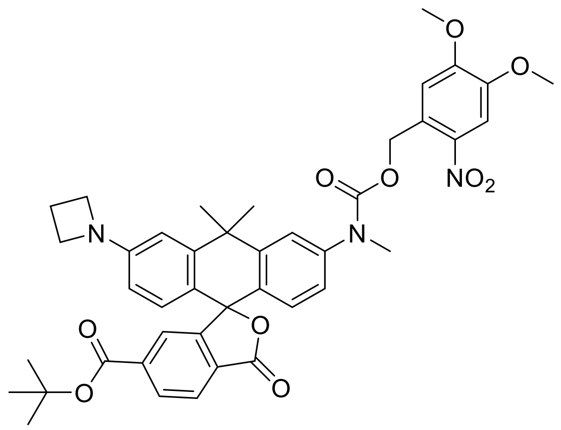

10

Compound 10: A mixture of compound **8**-Tf-^{t}Bu (20 mg, 24 µmol), azetidine hydrochloride, **9** (3.4 mg, 36 µmol, 1.5 eq), $Pd_2(dba)_3$ (1.1 mg, 1.2 µmol, 5 mol%), XPhos (1.7 mg, 4 µmol, 15 mol%) and Cs_2CO_3 (19 mg, 57 µmol, 2.4 eq) were combined in a microwave vial and flushed with argon. Degassed dry 1,4-dioxane (1 mL) was added, the vial was sealed and heated at 100 °C for 4 h in an oil bath. Upon cooling, the mixture was filtered through Celite, filter-cake washed with EtOAc, and the filtrate evaporated. The residue was purified by means of flash column chromatography on silica gel (0 – 80% ethyl acetate in hexane) to get compound **10** (8 mg, yield 45%) as an off-white solid.

^{1}H NMR (400 MHz, CD_3CN) δ 8.21 (dd, *J* = 8.0, 1.3 Hz, 1H), 8.06 (dd, *J* = 8.0, 0.8 Hz, 1H), 7.73 (d, *J* = 2.1 Hz, 1H), 7.69 (s, 1H), 7.50 (dd, *J* = 1.3, 0.8 Hz, 1H), 7.17 (dd, *J* = 8.5, 2.3 Hz, 1H), 6.88 (brs, 1H), 6.78 (dd,

J = 8.5, 0.4 Hz, 1H), 6.67 (d, J = 2.3 Hz, 1H), 6.58 (d, J = 8.5 Hz, 1H), 6.29 (dd, J = 8.6, 2.4 Hz, 1H), 5.47 (s, 2H), 3.92 (t, J = 7.3 Hz, 4H), 3.87 (s, 3H), 3.66 (brs, 3H), 3.35 (s, 3H), 2.43 – 2.32 (m, 2H), 1.81 (s, 3H), 1.69 (s, 3H), 1.51 (s, 9H). ^{13}C NMR (126 MHz, CD_3CN) δ 170.1, 165.0, 156.2, 155.4, 154.7, 153.9, 149.1, 147.6, 146.9, 145.1, 140.6, 139.2, 131.2, 130.3, 130.0, 129.5, 129.1, 128.3, 126.2, 125.5, 125.4, 124.8, 119.1, 111.8, 111.0, 109.1, 108.9, 87.5, 83.2, 65.2, 56.9, 56.9, 53.0, 39.1, 38.3, 34.9, 33.4, 28.1, 17.4. HRMS (ESI) calcd for $C_{42}H_{43}N_3O_{10}$ [M+Na]$^+$ 772.2841, found 772.2845.

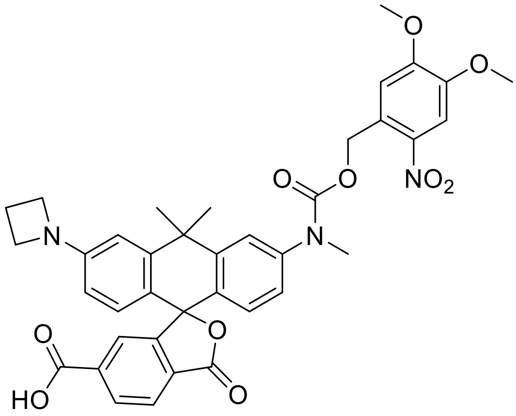

2-Azet-NRMe

Compound 2-Azet-NRMe: Trifluoroacetic acid (0.1 mL) was added to a solution of compound **10** (2.5 mg, 3 µmol) in CH_2Cl_2 (1 mL) at rt. The resulting solution was stirred at room temperature for 4 h. The course of the reaction was monitored by TLC. The reaction mixture was concentrated and co-distilled with acetonitrile in vacuum to get crude compound **2**-Azet-NRMe. It was purified by means of reverse phase HPLC (C18) [A:B 30:70 → 0:100 over 25 min, A – acetonitrile (0.1% TFA), B – water (0.1% TFA)] to get compound **2**-Azet-NRMe (2 mg, yield 86%) as a brown-solid.

^{1}H NMR (400 MHz, CD_3OD) δ 8.30 (dd, J = 8.0, 1.4 Hz, 1H), 8.15 (d, J = 8.3 Hz, 1H), 7.78 (d, J = 2.1 Hz, 1H), 7.71 (s, 1H), 7.62 (s, 1H), 7.21 (dd, J = 8.5, 2.2 Hz, 1H), 6.82 (d, J = 8.4 Hz, 2H), 6.81 (brs, 1H), 6.68 (s, 1H), 6.46 (d, J = 8.8 Hz, 1H), 5.50 (s, 2H), 4.13 – 4.02 (m, 4H), 3.88 (s, 3H), 3.65 (s, 3H), 3.38 (s, 3H), 2.44 (p, J = 7.5 Hz, 2H), 1.83 (s, 3H), 1.71 (s, 3H).

HRMS (ESI) calcd for $C_{38}H_{35}N_3O_{10}$ [M+H]$^+$ 716.2073, found 716.2215.

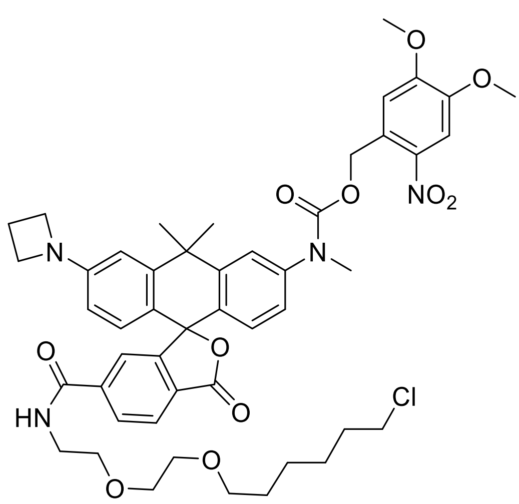

11

Compound 11: Compound **2**-Azet-NRMe (2.5 mg, 4 µmol, 1 eq), HaloTag-Amine (1.2 mg, 5 µmol, 1.5 eq) and *N,N*-diisopropylethylamine (4 µL, 22 µmol, 6 eq) were dissolved in DMF (1 mL), and then PyBOP (2.3 mg, 4 µmol, 1.2 eq) was added at room temperature. The reaction mixture was stirred at room temperature for 4 h, concentrated under vacuum, and the residue purified by means of reversed phase HPLC (Interchim) [A:B 30:70 → 0:100 over 25 min, A – acetonitrile (0.1% TFA), B – water (0.1% TFA)] to get compound **11** (1.5 mg, yield 47%) as a reddish brown solid.

^{1}H NMR (400 MHz, CD_3CN) δ 8.09 – 7.97 (m, 2H), 7.73 (d, *J* = 2.3 Hz, 1H), 7.69 (s, 1H), 7.35 (dd, *J* = 1.4, 0.8 Hz, 1H), 7.21 (t, *J* = 6.4 Hz, 1H), 7.17 (dd, *J* = 8.5, 2.2 Hz, 1H), 6.89 (brs, 1H), 6.78 (d, *J* = 8.4 Hz, 1H), 6.70 (d, *J* = 2.4 Hz, 1H), 6.60 (d, *J* = 8.6 Hz, 1H), 6.33 (dd, *J* = 8.6, 2.4 Hz, 1H), 5.47 (s, 2H), 3.94 (t, *J* = 7.3 Hz, 4H), 3.88 (s, 3H), 3.67 (brs, 3H), 3.58 – 3.49 (m, 6H), 3.48 – 3.41 (m, 4H), 3.36 (s, 3H), 3.32 (t, *J* = 6.5 Hz, 2H), 2.45 – 2.31 (m, 2H), 1.82 (s, 3H), 1.75 – 1.66 (m, 2H), 1.69 (s, 3H), 1.48 – 1.40 (m, 2H), 1.39 – 1.32 (m, 2H), 1.31 – 1.22 (m, 2H). ^{13}C NMR (126 MHz, CD_3CN) δ 170.2, 166.4, 155.4, 154.7, 153.7, 149.1, 147.6, 147.1, 145.1, 145.0, 142.2, 140.6, 140.6, 129.8, 129.4, 129.3, 129.3, 128.3, 126.2, 125.4, 125.3, 123.2, 119.6, 112.0, 111.1, 109.2, 109.2, 71.5, 70.8, 70.7, 69.8, 65.2, 57.0, 56.9, 53.1, 46.2, 40.5, 39.2, 38.3, 34.9, 33.4, 33.3, 30.2, 27.3, 26.1, 17.4.

HRMS (ESI) calcd for $C_{48}H_{55}ClN_4O_{11}$ $[M+Na]^+$ 921.3448, found 921.3431.

Scheme S4

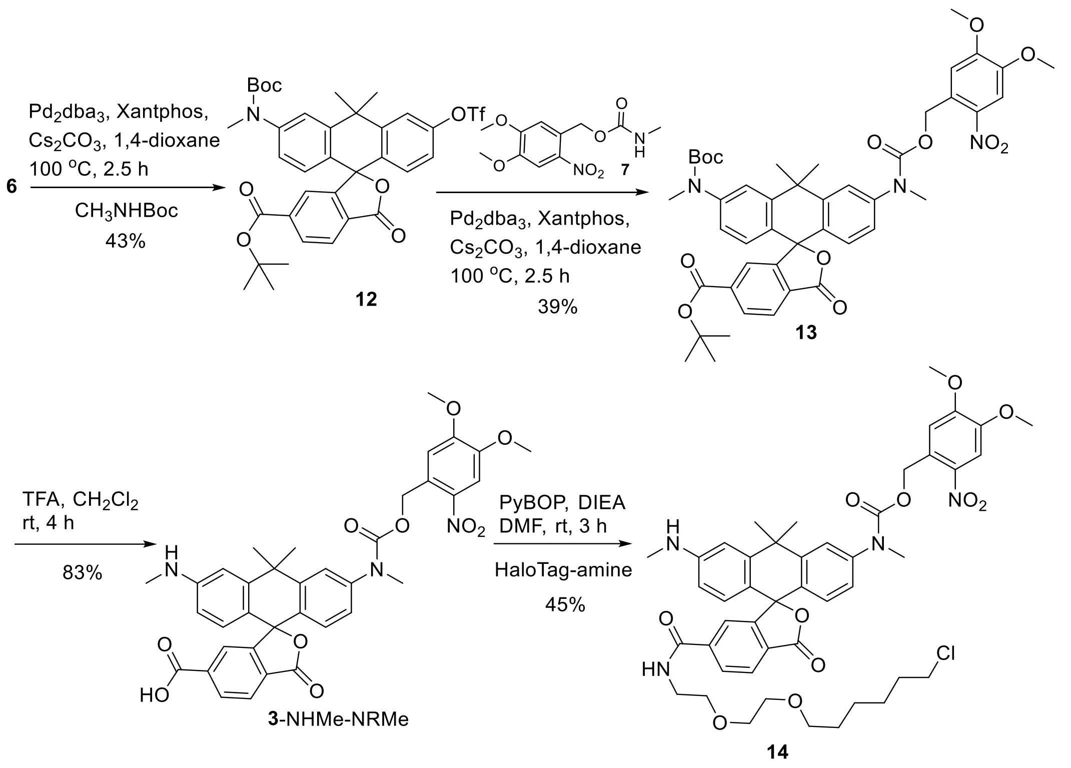

Scheme S4. Synthesis of mono-caged rhodol carboxylic acid **3**-NHMe-NRMe and HaloTag derivative **14**

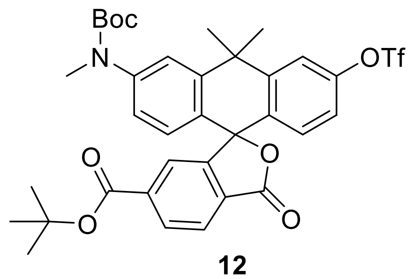

12

Compound 12: A mixture of **6** (40 mg, 53 µmol), *tert*-butyl-*N*-methylcarbamate (9 mg, 64 µmol, 1.2 eq), $Pd_2(dba)_3$ (2.5 mg, 2.6 µmol, 5 mol%), Xantphos (4.6 mg, 8 µmol, 15 mol%) and Cs_2CO_3 (25 mg, 74 µmol, 1.4 eq) were taken in a microwave vial and flushed with argon. Degassed dry 1,4-dioxane (1.5 mL) was added, the vial sealed and heated at 100 °C for 2.5 h in an oil bath. Upon cooling, the reaction mixture was filtered through Celite, filter-cake washed with EtOAc, and evaporated. The residue was purified by flash column chromatography on silica gel (0 – 40% ethyl acetate in hexane) to get compound **12** (16 mg, 43%) as off-white solid.

^{1}H NMR (400 MHz, $CDCl_3$) δ 8.22 (dd, *J* = 8.0, 1.3 Hz, 1H), 8.09 (dd, *J* = 8.0, 0.8 Hz, 1H), 7.61 (dd, *J* = 1.3, 0.8 Hz, 1H), 7.56 (d, *J* = 2.4 Hz, 2H), 7.08 (dd, *J* = 8.8, 2.6 Hz, 1H), 7.06 (dd, *J* = 8.6, 2.3 Hz, 1H), 6.88 (d, *J* = 8.8 Hz, 1H), 6.71 (d, *J* = 8.6 Hz, 1H), 3.29 (s, 3H), 1.90 (s, 3H), 1.80 (s, 3H), 1.56 (s, 9H), 1.48 (s, 9H).

^{13}C NMR (126 MHz, $CDCl_3$) δ 169.1, 163.9, 154.4, 150.0, 148.0, 145.0, 144.2, 138.4, 131.5, 130.8, 130.1, 128.9, 128.0, 126.6, 125.4, 124.6, 123.9, 123.3, 120.0, 119.9, 119.7, 117.4, 85.0, 82.7, 80.9, 38.6, 37.14, 34.7, 33.2, 28.4, 28.0.

HRMS (ESI) calcd for $C_{35}H_{36}F_3NO_9S$ $[M+Na]^+$ 726.1955, found 726.1948.

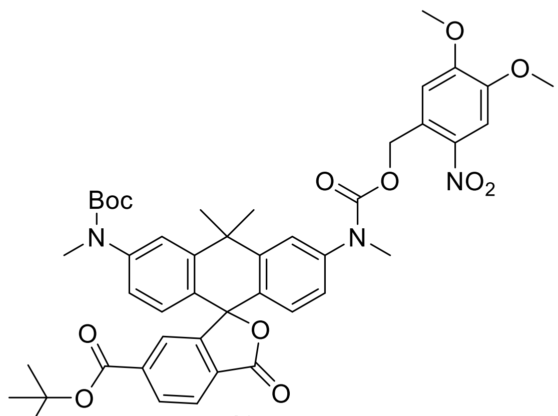

13

Compound 13: A mixture of **12** (22 mg, 31 µmol), compound **7** (10 mg, 38 µmol, 2.4 eq), $Pd_2(dba)_3$ (1.5 mg, 2 µmol, 5 mol%), Xantphos (2.7 mg, 5 µmol, 15 mol%) and Cs_2CO_3 (14 mg, 44 µmol, 1.4 eq) were combined in a microwave vial and flushed with argon. Degassed dry 1,4-dioxane (1 mL) was added, the vial was sealed and heated at 100 °C for 2.5 h in an oil bath. Upon cooling, the resulting mixture was filtered through Celite, filter-cake washed with EtOAc, and evaporated. The residue was separated by means of flash column chromatography on silica gel (0 – 80% ethyl acetate in hexane) to get compound **13** (10 mg, yield 39%) as off-white solid.

^{1}H NMR (400 MHz, $CDCl_3$) δ 8.20 (dd, *J* = 8.0, 1.3 Hz, 1H), 8.07 (dd, *J* = 8.0, 0.7 Hz, 1H), 7.69 (s, 1H), 7.61 (d, *J* = 7.8 Hz, 2H), 7.54 (d, *J* = 2.3 Hz, 1H), 7.09 (dd, *J* = 8.5, 2.2 Hz, 1H), 7.05 (dd, *J* = 8.6, 2.2 Hz, 1H), 6.77 (d, *J* = 8.5 Hz, 1H), 6.73 (s, 1H), 6.71 (d, *J* = 8.6 Hz, 1H), 5.58 (s, 2H), 3.93 (s, 3H), 3.71 (s, 3H), 3.39 (s, 3H), 3.29 (s, 3H), 1.86 (s, 3H), 1.74 (s, 3H), 1.55 (s, 9H), 1.48 (s, 9H). ^{13}C NMR (126 MHz, $CDCl_3$) δ 169.4, 164.1, 154.8, 154.6, 154.4, 153.5, 148.1, 148.0, 146.0, 144.8, 144.8, 143.8, 138.2, 130.5, 129.0, 128.4, 128.0, 127.5, 126.9, 125.2, 124.7, 123.7, 123.4, 123.4, 110.4, 108.2, 108.1, 108.1, 85.5, 82.6, 80.8, 64.8, 56.4, 56.4, 38.2, 37.1, 34.6, 33.4, 28.4, 28.0.

HRMS (ESI) calcd for $C_{45}H_{49}N_3O_{12}$ $[M+Na]^+$ 846.3208, found 846.3209.

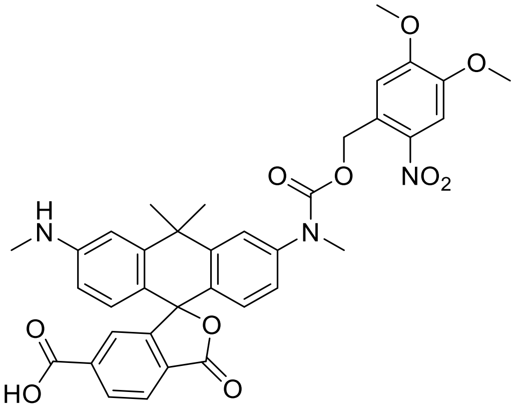

3-NHMe-NRMe

Compound 3-NHMe-NRMe: Trifluoroacetic acid (0.2 mL) was added to a solution of **13** (6 mg, 10 µmol) in CH_2Cl_2 (1.5 mL) at room temperature. The reaction mixture was stirred at room temperature for 4 h. TLC and HPLC showed the formation of a product. The reaction mixture was concentrated and co-distilled with acetonitrile in vacuum to get crude compound **3**-NHMe-NRMe. It was purified by means of reverse phase HPLC (C18) [A:B 30:70 → 0:100 over 25 min, A – acetonitrile (0.1% TFA), B – water (0.1% TFA)] to get compound **3**-NHMe-NRMe (4 mg, yield 83%) as a brown solid.

^{1}H NMR (400 MHz, CD_3CN) δ 8.23 (dd, *J* = 8.0, 1.3 Hz, 1H), 8.08 (dd, *J* = 8.0, 0.8 Hz, 1H), 7.73 (d, *J* = 2.2 Hz, 1H), 7.69 (s, 1H), 7.55 (dd, *J* = 1.3, 0.8 Hz, 1H), 7.17 (dd, *J* = 8.5, 2.3 Hz, 1H), 6.88 (d, *J* = 2.4 Hz, 2H), 6.78 (dd, *J* = 8.5, 0.4 Hz, 1H), 6.55 (d, *J* = 8.6 Hz, 1H), 6.47 (dd, *J* = 8.7, 2.4 Hz, 1H), 5.47 (s, 2H), 3.88 (s, 3H), 3.67 (s, 3H), 3.35 (s, 3H), 2.82 (s, 3H), 1.81 (s, 3H), 1.69 (s, 3H).

HRMS (ESI) calcd for $C_{36}H_{33}N_3O_{10}$ $[M+H]^+$ 668.2239, found 668.2235.

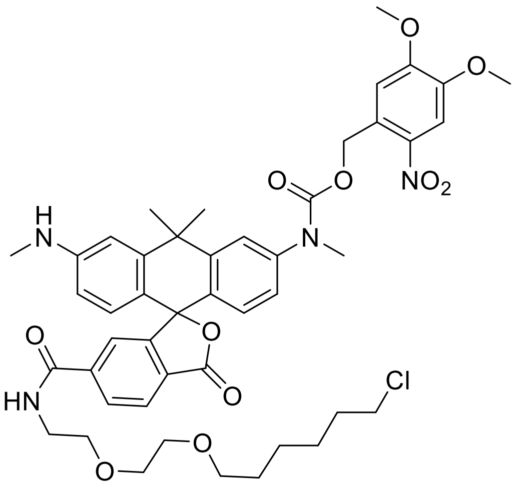

14

Compound 14: Compound **3**-NHMe-NRMe (2.5 mg, 4 µmol, 1 eq), HaloTag-amine (2 mg, 6 µmol, 1.5 eq) and *N,N*-diisopropylethylamine (8 µL, 40 µmol, 10 eq) were combined in DMF (1 mL). Then PyBOP

(3 mg, 6 µmol, 1.5 eq) was added at room temperature. The reaction mixture was stirred at room temperature for 3 h, concentrated under vacuum, and the residue purified by means of reversed phase flash chromatography (C18), [A:B 30:70 → 0:100 over 25 min, A – acetonitrile (0.1% TFA), B – water + (0.1% TFA)] to get compound **14** (1.5 mg, yield 45%) as a brown solid.

^{1}H NMR (400 MHz, CD_3CN) δ 8.09 – 7.98 (m, 2H), 7.73 (d, *J* = 2.2 Hz, 1H), 7.69 (s, 1H), 7.36 (dd, *J* = 1.3, 0.9 Hz, 1H), 7.22 (t, *J* = 6.4 Hz, 1H), 7.17 (dd, *J* = 8.5, 2.3 Hz, 1H), 6.89 (brs, 1H), 6.86 (d, *J* = 2.4 Hz, 1H), 6.77 (d, *J* = 8.6 Hz, 1H), 6.54 (d, *J* = 8.7 Hz, 1H), 6.46 (dd, *J* = 8.7, 2.4 Hz, 1H), 5.47 (s, 2H), 3.88 (s, 3H), 3.67 (brs, 3H), 3.56 – 3.50 (m, 6H), 3.47 – 3.41 (m, 4H), 3.36 (s, 3H), 3.32 (t, *J* = 6.5 Hz, 2H), 2.81 (s, 3H), 1.81 (s, 3H), 1.72 – 1.67 (m, 2H), 1.69 (s, 3H), 1.48 – 1.40 (m, 2H), 1.38 – 1.32 (m, 2H), 1.31 – 1.26 (m, 2H).

HRMS (ESI) calcd for $C_{46}H_{53}ClN_4O_{11}$ [M+H]$^+$ 895.3292, found 895.3268.

Scheme S5

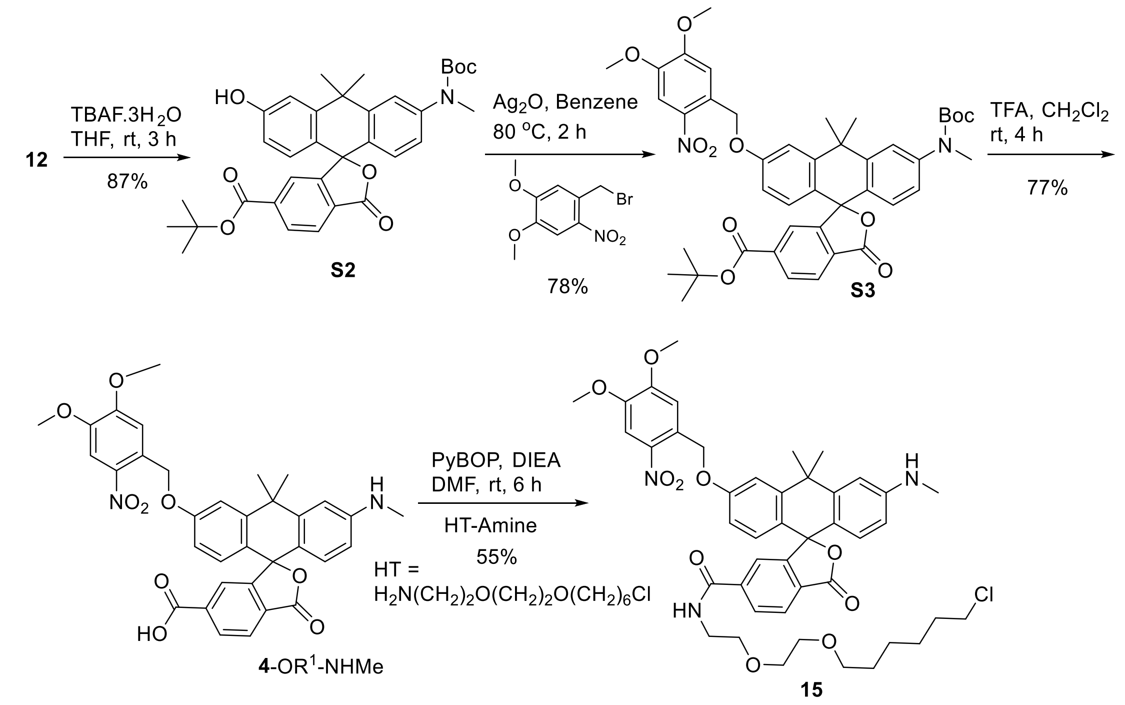

Scheme S5. Synthesis of *O*-protected mono-caged rhodol COOH, **4**-OR^1-NHMe and HaloTag derivative **15**

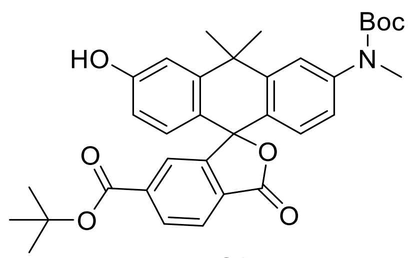

S2

Compound S2: TBAF*3H_2O (20 mg) was added to a solution of compound **12** (16 mg, 23 µmol) in THF (2 mL) at room temperature. The reaction mixture was stirred at room temperature for 3 h. THF was evaporated, and the reaction mixture acidified with 1 M aq HCl, extracted with ethyl acetate (4×), dried (Na_2SO_4) and concentrated in vacuum. Crude product was purified by means of flash chromatography on silica gel (0 – 80% ethyl acetate in *n*-hexane) to get compound **S2** (11.3 mg, yield 87%) as an off-white solid.

^{1}H NMR (400 MHz, $CDCl_3$) δ 8.18 (dd, *J* = 8.0, 1.3 Hz, 1H), 8.05 (d, *J* = 7.9 Hz, 1H), 7.61 (d, *J* = 1.2 Hz, 1H), 7.53 (d, *J* = 1.9 Hz, 1H), 7.11 (s, 1H), 7.02 (d, *J* = 8.3 Hz, 1H), 6.69 (d, *J* = 8.5 Hz, 1H), 6.63 (s, 2H), 5.26 (brs, 1H), 3.29 (s, 3H), 1.85 (s, 3H), 1.75 (s, 3H), 1.55 (s, 9H), 1.48 (s, 9H). ^{13}C NMR (126 MHz, $CDCl_3$) δ 170.7, 165.1, 157.4, 156.1, 155.5, 148.1, 146.4, 145.5, 139.0, 131.3, 130.5, 130.3, 129.0, 128.6, 126.0, 125.8, 124.5, 124.5, 124.1, 115.8, 113.8, 87.4, 83.5, 81.8, 39.2, 38.2, 35.8, 34.0, 29.4, 29.0.

HRMS (ESI) calcd for $C_{34}H_{37}NO_7$ $[M+H]^+$ 672.2645, found 672.2643.

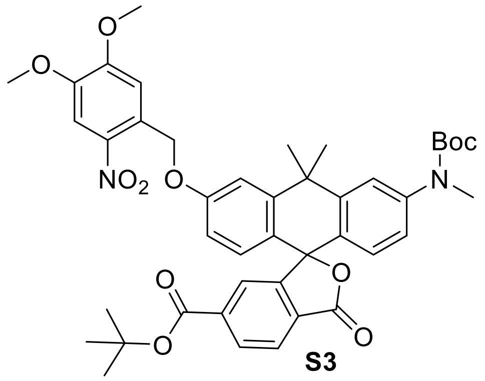

S3

Compound S3: A mixture of compound **S2** (9 mg, 16 µmol, 1 eq), 4,5-dimethoxy-2-nitro-benzyl bromide (7 mg, 23 µmol, 1.5 eq) and Ag_2O (4.5 mg, 19 µmol, 1.2 eq) in benzene (2 mL) was stirred at 80 °C for 6 h in a flask equipped with reflux condenser and a tube (on top) filled with drying agent (mol sieves). The reaction mixture was cooled, diluted with water and extracted with ethyl acetate (3 x). The combined organic solutions were washed with brine, dried (Na_2SO_4), and concentrated. The residue was purified by means of flash chromatography on silica gel (0 – 40% ethyl acetate in hexane) to obtain compound **S3** (10 mg, yield 78%) as an off-white-solid.

^{1}H NMR (400 MHz, $CDCl_3$) δ 8.19 (dd, *J* = 8.0, 1.3 Hz, 1H), 8.06 (dd, *J* = 8.0, 0.8 Hz, 1H), 7.78 (s, 1H), 7.62 (t, *J* = 1.0 Hz, 1H), 7.55 (d, *J* = 2.2 Hz, 1H), 7.33 (s, 1H), 7.28 (s, 1H), 7.03 (dd, *J* = 8.6, 2.2 Hz, 1H), 6.81 (dd, *J* = 8.8, 2.6 Hz, 1H), 6.72 (d, *J* = 8.8, 1H), 6.70 (d, *J* = 8.6, 1H), 5.53 (s, 2H), 3.99 (s, 3H), 3.98 (s, 3H), 3.29 (s, 3H), 1.88 (s, 3H), 1.77 (s, 3H), 1.55 (s, 9H), 1.48 (s, 9H). ^{13}C NMR (126 MHz, $CDCl_3$) δ 170.6, 165.1, 159.8, 155.9, 155.5, 155.0, 149.0, 148.1, 146.3, 145.6, 140.1, 139.0, 131.4, 130.5, 130.3, 129.7, 129.0, 128.5, 126.1, 125.7, 125.1, 124.5, 124.5, 115.0, 113.8, 110.5, 109.1, 87.2, 83.5, 81.7, 68.2, 57.5, 57.4, 39.4, 38.2, 35.9, 34.1, 29.4, 29.0.

HRMS (ESI) calcd for $C_{43}H_{46}N_2O_{11}$ [M+Na]$^+$ 789.3001, found 789.2994.

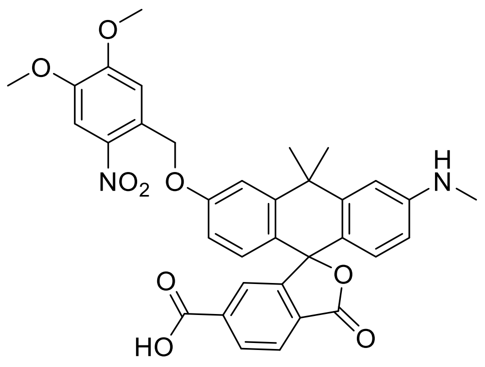

4-OR[1]-NHMe

Compound 4-OR[1]-NHMe: Trifluoroacetic acid (0.2 mL) was added to a solution of **S3** (8 mg, 10 µmol) in CH_2Cl_2 (2 mL) at room temperature. The reaction mixture was stirred at room temperature for 4 h. TLC and HPLC showed the formation of a product. The reaction mixture was concentrated and co-distilled in vacuum with acetonitrile to get crude compound **4**-OR[1]-NHMe. It was isolated by means of reversed phase prep. HPLC (Interchim) [A:B 30:70 → 0:100 over 25 min, A – acetonitrile (0.1% TFA), B – water + (0.1% TFA)] to obtain pure compound **4**-OR[1]-NHMe (5 mg, yield 77%) as a light brown solid.

^{1}H NMR (400 MHz, CD_3CN) δ 8.22 (dd, *J* = 8.0, 1.4 Hz, 1H), 8.07 (dd, *J* = 8.0, 0.8 Hz, 1H), 7.74 (s, 1H), 7.56 (dd, *J* = 1.3, 0.7 Hz, 1H), 7.38 (d, *J* = 2.6 Hz, 1H), 7.33 (s, 1H), 6.93 (d, *J* = 2.4 Hz, 1H), 6.86 (dd, *J* = 8.8, 2.6 Hz, 1H), 6.74 (d, *J* = 8.8 Hz, 1H), 6.57 (d, *J* = 8.6 Hz, 1H), 6.49 (dd, *J* = 8.7, 2.4 Hz, 1H), 5.51 (s, 2H), 3.92 (s, 3H), 3.91 (s, 3H), 2.84 (s, 3H), 1.85 (s, 3H), 1.74 (s, 3H).

HRMS (ESI) calcd for $C_{34}H_{30}N_2O_9$ [M+H]$^+$ 611.2024, found 611.2029.

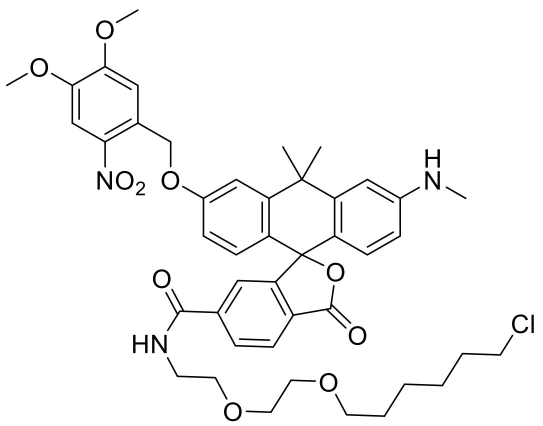

15

Compound 15: Compound **4**-OR[1]-NHMe (3 mg, 5 µmol, 1 eq), HaloTag-Amine (1.6 mg, 7 µmol, 1.5 eq) and *N,N*-diisopropylethylamine (7 µL, 30 µmol, 10 eq) were dissolved in DMF (1 mL). Then PyBOP (3.6 mg, 7 µmol, 1.5 eq) was added at room temperature, and the reaction mixture stirred for 6 h. The reaction mixture was concentrated under vacuum, and the residue subjected to prep. HPLC (Interchim)

[A:B 30:70 → 0:100 over 25 min, A – acetonitrile (0.1% TFA), B – water + (0.1% TFA)] to get compound **15** (2 mg, yield: 55%) as light pink solid.

^{1}H NMR (400 MHz, CD_3CN) δ 8.02 (dd, *J* = 0.7 Hz, 2H), 7.74 (s, 1H), 7.38 (d, *J* = 2.6 Hz, 1H), 7.36 – 7.28 (m, 2H), 7.22 (t, *J* = 5.5 Hz, 1H), 6.92 – 6.81 (m, 2H), 6.72 (d, *J* = 8.8 Hz, 1H), 6.53 (d, *J* = 8.6 Hz, 1H), 6.45 (dd, *J* = 8.7, 2.4 Hz, 1H), 5.50 (s, 2H), 3.92 (s, 6H), 3.58 – 3.49 (m, 6H), 3.48 – 3.40 (m, 4H), 3.32 (t, *J* = 6.5 Hz, 3H), 2.81 (s, 3H), 1.86 (s, 3H), 1.74 (s, 3H), 1.72 – 1.64 (m, 2H), 1.46 – 1.40 (m, 2H), 1.39 – 1.34 (m, 2H), 1.28 – 1.22 (m, 2H).

HRMS (ESI) calcd for $C_{44}H_{50}ClN_3O_{10}$ $[M+H]^+$ 816.3257, found 816.3248.

Scheme S6

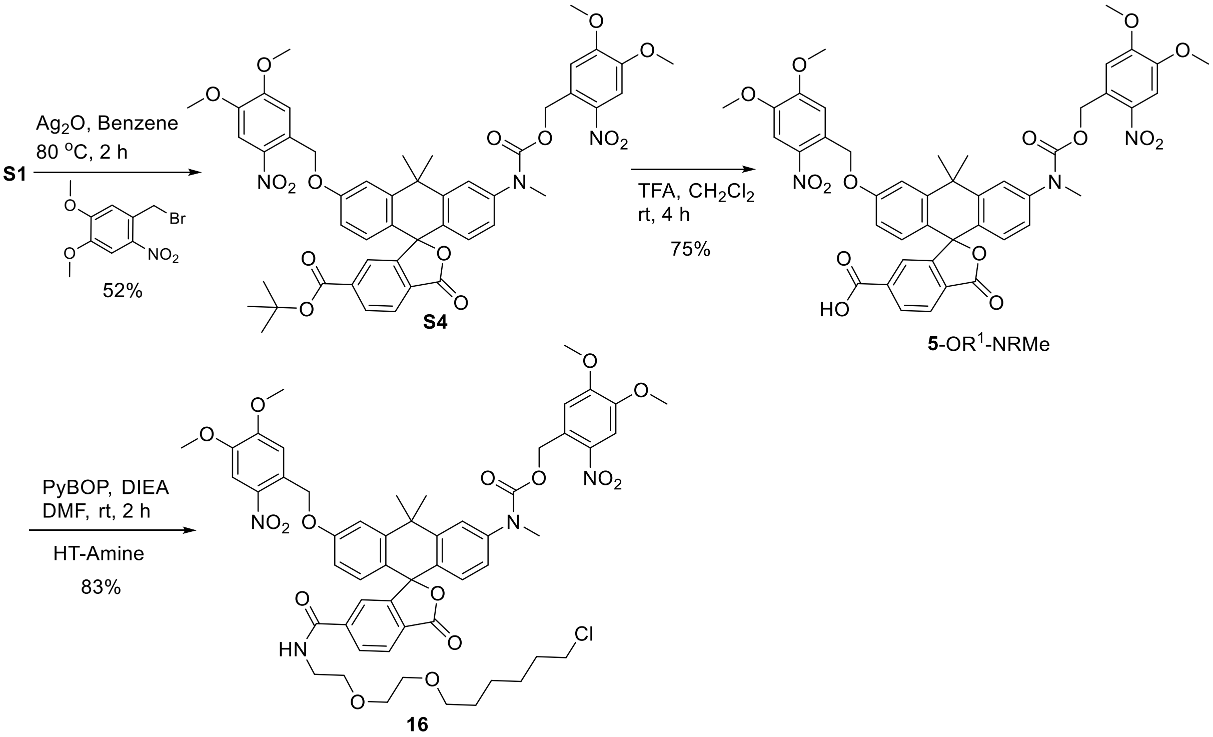

Scheme S6. Synthesis of mono-caged rhodol COOH, **5**-OR[1]-NRMe and HaloTag derivative **16**

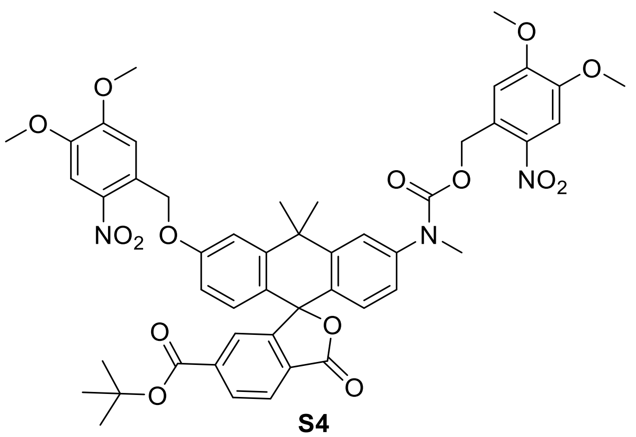

S4

Compound S4: A mixture of compound **S1** (30 mg, 42 μmol, 1 eq), 4,5-dimethoxy-2-nitro-benzyl bromide (17.5 mg, 63 μmol, 1.5 eq) and Ag_2O (11 mg, 46 μmol, 1.1 eq) were combined in benzene (3 mL) in a flask equipped with a reflux condenser and a tube with drying agent. The reaction mixture was heated at 80 °C for 2 h, cooled to room temperature, diluted with water, and extracted with ethyl acetate (3x). The combined organic solutions were washed with brine, dried (Na_2SO_4), and concentrated. The residue was subjected to flash column chromatography on silica gel (0 – 50% ethyl acetate in hexane) to obtain compound **S4** (20 mg, yield 52%) as an off-white solid.

^1H NMR (400 MHz, $CDCl_3$) δ 8.20 (dd, *J* = 8.0, 1.3 Hz, 1H), 8.07 (dd, *J* = 8.1, 0.8 Hz, 1H), 7.78 (s, 1H), 7.69 (s, 1H), 7.63 (s, 1H), 7.60 (s, 1H), 7.33 (s, 1H), 7.09 (dd, *J* = 8.5, 2.2 Hz, 1H), 6.82 (dd, *J* = 8.8, 2.6 Hz, 1H), 6.76 (d, *J* = 8.5 Hz, 2H), 7.75 (brs, 1H), 6.72 (d, *J* = 8.8 Hz, 1H), 5.58 (s, 2H), 5.52 (s, 2H), 3.99 (s, 3H), 3.98 (s, 3H), 3.93 (s, 3H), 3.71 (s, 3H), 3.39 (s, 3H), 1.86 (s, 3H), 1.73 (s, 3H), 1.55 (s, 9H). ^{13}C NMR (126 MHz, $CDCl_3$) δ 170.3, 165.1, 159.9, 155.8, 155.6, 154.9, 154.5, 149.0, 147.7, 147.1, 144.8, 140.5, 140.2, 139.3, 139.2, 131.5, 130.6, 130.2, 129.6, 129.4, 128.5, 126.2, 125.7, 125.3, 125.2, 125.1, 125.1, 124.8, 115.1, 113.8, 110.5, 109.1, 109.1, 86.8, 83.6, 68.2, 65.8, 57.5, 57.4, 57.3 (2 x OCH3), 39.4, 38.8, 35.8, 34.2, 29.0.

HRMS (ESI) calcd for $C_{48}H_{47}N_3O_{15}$ $[M+Na]^+$ 928.2899, found 928.2902.

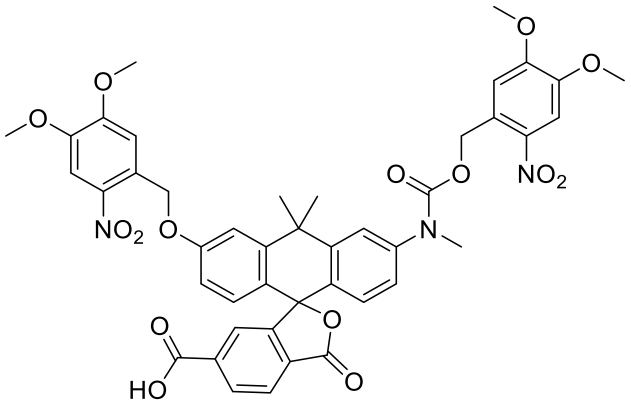

5-OR[1]-NRMe

Compound 5-OR[1]-NRMe: Trifluoroacetic acid (0.3 mL) was added to a solution of **S4** (15 mg, 17 μmol) in CH_2Cl_2 (2 mL) at room temperature. The rection mixture was stirred at room temperature for 4 h. TLC and HPLC showed the formation of a product. The reaction mixture was concentrated and co-distilled in vacuum with acetonitrile to get crude compound **5**-OR[1]-NRMe. It was isolated and purified by means of flash column chromatography on silica gel (0 – 5% methanol in CH_2Cl_2) to afford compound **5**-OR[1]-NRMe (11 mg, yield 75%) as a light pink solid.

^1H NMR (400 MHz, CD_3CN) δ 8.28 – 8.21 (m, 1H), 8.10 (d, *J* = 8.0 Hz, 1H), 7.74 (d, *J* = 1.8 Hz, 2H), 7.68 (s, 1H), 7.56 (d, *J* = 1.1 Hz, 1H), 7.37 (d, *J* = 2.6 Hz, 1H), 7.31 (s, 1H), 7.19 (dd, *J* = 8.5, 2.2 Hz, 1H), 6.89

(dd, *J* = 8.8, 2.6 Hz, 1H), 6.88 (brs, 1H), 6.81 (d, *J* = 8 Hz, 1H), 6.77 (d, *J* = 8 Hz, 1H), 5.50 (s, 2H), 5.46 (s, 2H), 3.91 (s, 3H), 3.90 (s, 3H), 3.88 (s, 3H), 3.67 (s, 3H), 3.35 (s, 3H), 1.82 (s, 3H), 1.70 (s, 3H). ^{13}C NMR (126 MHz, CD_3CN) δ 170.0, 166.4, 160.1, 156.1, 155.4, 154.7, 154.6, 149.3, 149.1, 147.9, 147.0, 145.2, 140.9, 140.6, 137.8, 131.7, 130.4, 130.3, 129.5, 129.2, 128.5, 128.2, 126.5, 125.7, 125.4, 125.3, 124.4, 115.2, 113.9, 111.7, 111.1, 109.3, 109.2, 86.6, 67.9, 65.2, 57.0, 56.9 (2 x OCH_3), 56.9, 39.2, 38.2, 34.7, 33.5.

HRMS (ESI) calcd for $C_{44}H_{39}N_3O_{15}$ $[M+Na]^+$ 872.2273, found 872.2270.

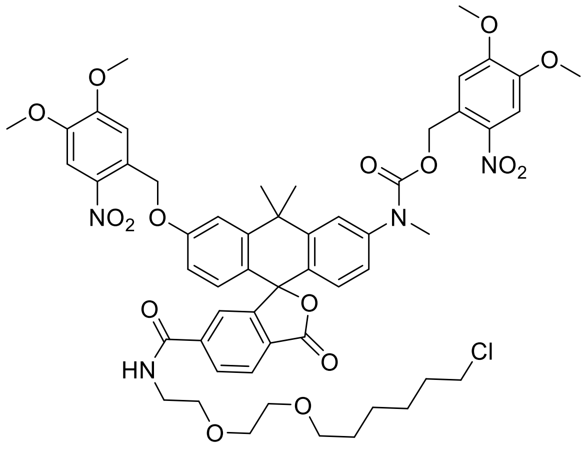

16

Compound 16: Compound **5**-OR[1]-NRMe (4 mg, 5 µmol, 1 eq), HaloTag-Amine (1.6 mg, 7 µmol, 1.5 eq) and *N,N*-diisopropylethylamine (8 µL, 40 µmol, 10 eq) were combined in DMF (1 mL), and then PyBOP (3.7 mg, 7 µmol, 1.5 eq) was added at room temperature. The reaction mixture was stirred for 2 h, concentrated under vacuum, and the residue subjected to reversed phase prep. HPLC (Interchim) [A:B 30:70 → 0:100 over 25 min, A – acetonitrile (0.1% TFA), B – water (0.1% TFA)] to get compound **16** (4.3 mg, yield 83%) as pink solid.

^{1}H NMR (400 MHz, $CDCl_3$) δ 8.08 (dd, *J* = 8.0, 0.7 Hz, 1H), 7.95 (dd, *J* = 8.0, 1.4 Hz, 1H), 7.78 (s, 1H), 7.69 (s, 1H), 7.59 (s, 1H), 7.49 (s, 1H), 7.33 (s, 1H), 7.26 (d, *J* = 2.7 Hz, 1H), 7.08 (dd, *J* = 8.5, 2.2 Hz, 1H), 6.88 (d, *J* = 5.5 Hz, 1H), 6.82 (dd, *J* = 8.8, 2.5 Hz, 1H), 6.73 (dd, *J* = 16.4, 8.6 Hz, 2H), 5.57 (s, 2H), 5.49 (s, 2H), 3.99, (s, 3H), 3.98 (s, 3H), 3.93 (s, 3H), 3.72 (s, 3H), 3.66 – 3.54 (m, 6H), 3.59 – 3.48 (m, 2H), 3.52 (t, *J* = 6.6 Hz, 2H), 3.42 (t, *J* = 6.7 Hz, 2H), 3.36 (s, 3H), 1.81 – 1.70 (m, 2H), 1.72 (s, 3H), 1.55 (p, *J* = 6.9 Hz, 2H), 1.47 – 1.39 (m, 2H), 1.37 – 1.31 (m, 2H), 1.26 (s, 3H).

HRMS (ESI) calcd for $C_{54}H_{59}ClN_4O_{16}$ $[M+Na]^+$ 1077.3507, found 1077.3515.

Supplementary References

[1] Uno, K., Bossi, M. L., Konen, T., Belov, V. N., Irie, M., Hell, S. W., *Adv. Opt. Mater.* **2019**, *7*, 1801746.

[2] Scott D. W., *Ann. Stat.* **1985**, *13*, 1024–1040.

[3] Aktalay, A., Khan, T. A., Bossi, M. L., Belov, V. N., Hell, S. W., *Angew. Chem., Int. Ed.* **2023**, *62*, 202302781.

[4] So, M.-K., Yao, H., Rao, J., *Biochem. Biophys. Res. Comm.* **2008**, *374*, 419–423.

[5] Landvatter, S. W., Katzenellenbogen, J. A., *J. Med. Chem.* **1982**, *25*, 1300–1307.

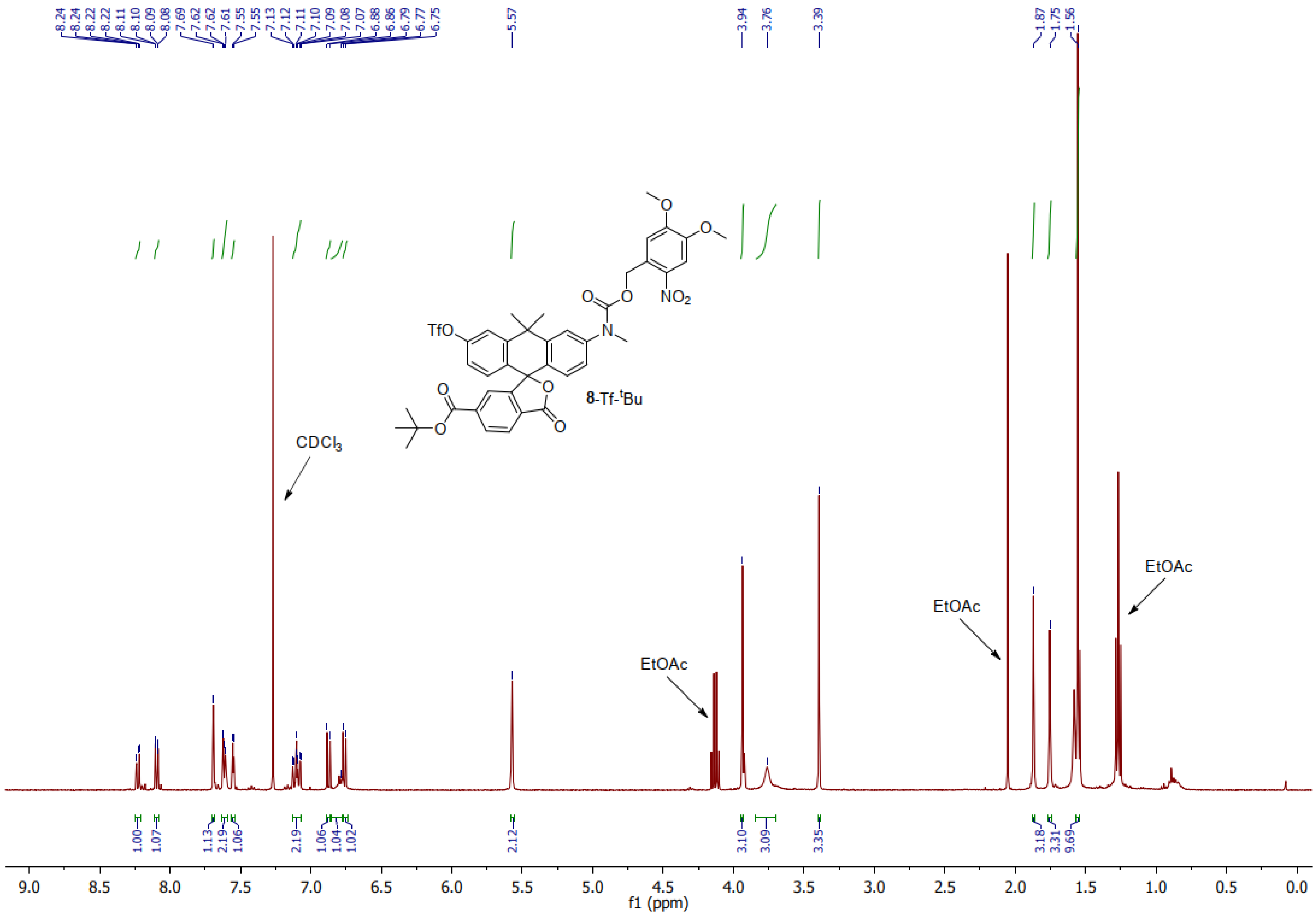

8.24
8.24
8.22
8.22
8.11
8.10
8.09
8.08
7.69
7.62
7.62
7.61
7.55
7.55
7.13
7.12
7.11
7.10
7.09
7.08
7.07
6.88
6.86
6.79
6.77
6.75
5.57
3.94
3.76
3.39
1.87
1.75
1.56
TfO
N
O
O
NO2
O
O
O
O
O
CDCl3
8-Tf-tBu
EtOAc
EtOAc
EtOAc
1.00
1.07
1.13
2.19
1.06
2.19
1.06
1.04
1.02
2.12
3.10
3.09
3.35
3.18
3.31
9.69
9.0
8.5
8.0
7.5
7.0
6.5
6.0
5.5
5.0
4.5
4.0
3.5
3.0
2.5
2.0
1.5
1.0
0.5
0.0
f1 (ppm)

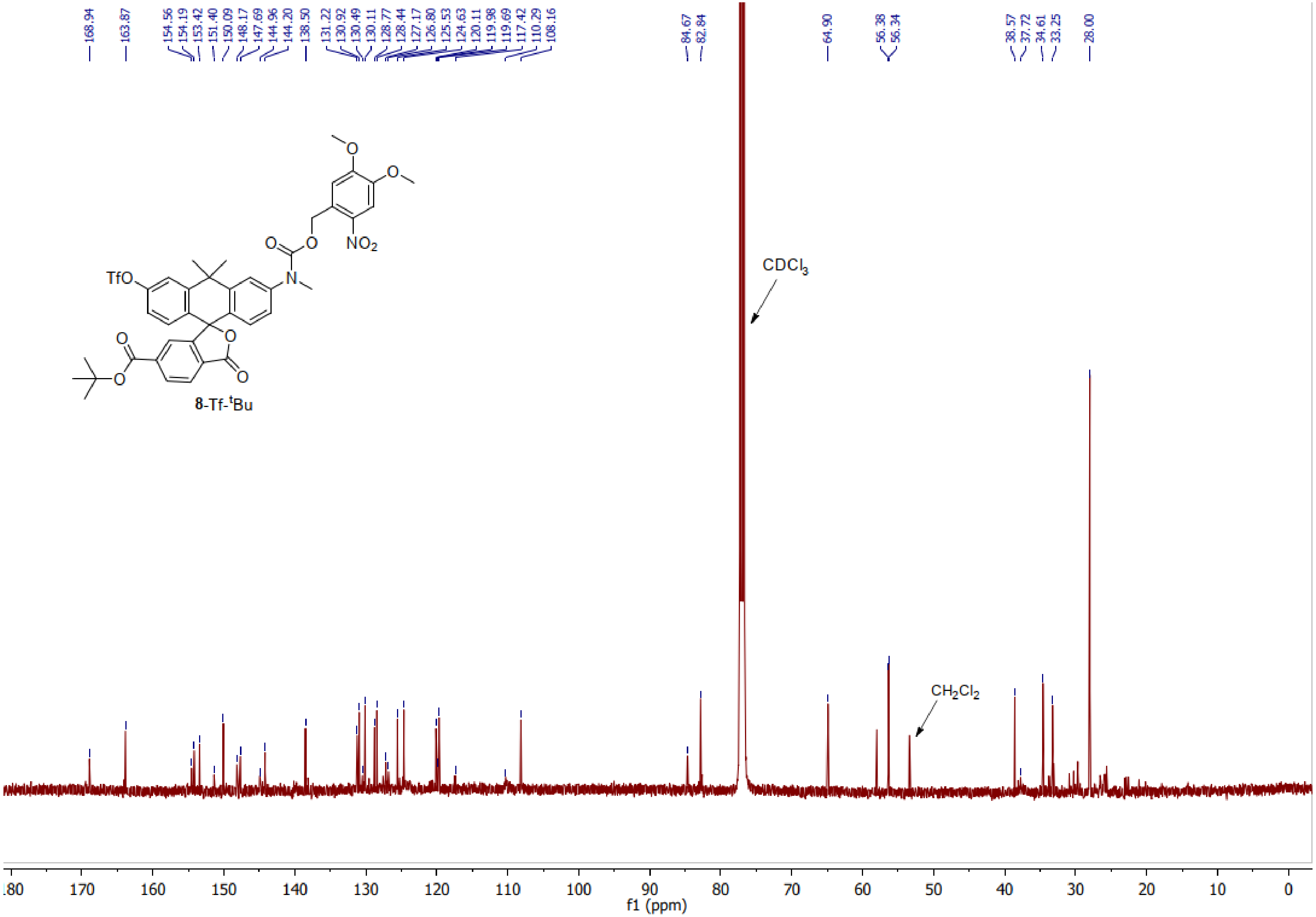

168.94
163.87
154.56
154.19
153.42
151.40
150.09
148.17
147.69
144.96
144.20
138.50
131.22
130.92
130.49
130.11
128.77
128.44
127.17
126.80
125.53
124.63
120.11
119.98
119.69
117.42
110.29
108.16
84.67
82.84
64.90
56.38
56.34
38.57
37.72
34.61
33.25
28.00
TfO
NO2
8-Tf-tBu
CDCl3
CH2Cl2
f1 (ppm)

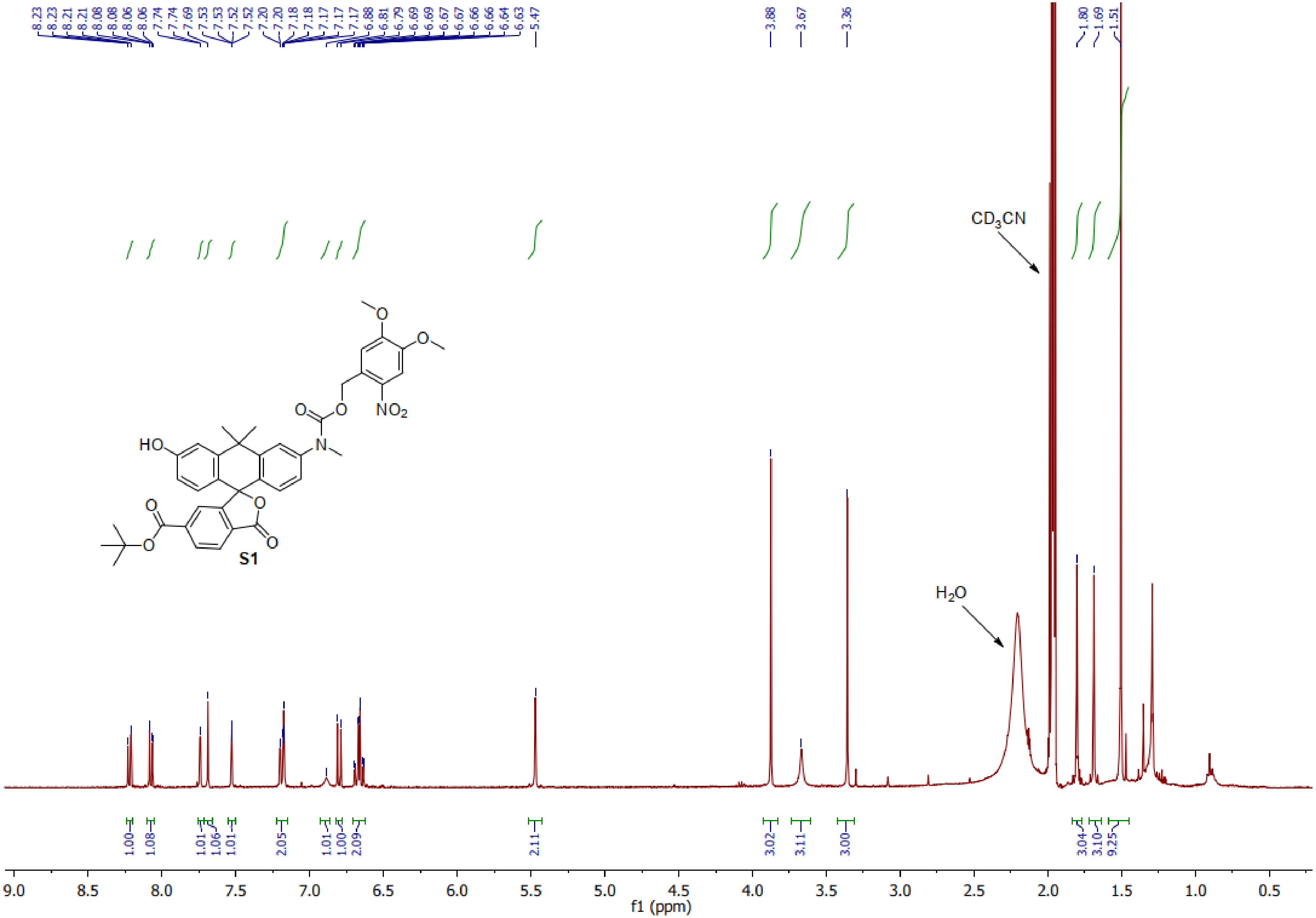
CD3CN
H2O
HO
O
O
O
N
O
O
NO2
O
O
O
O
S1
f1 (ppm)

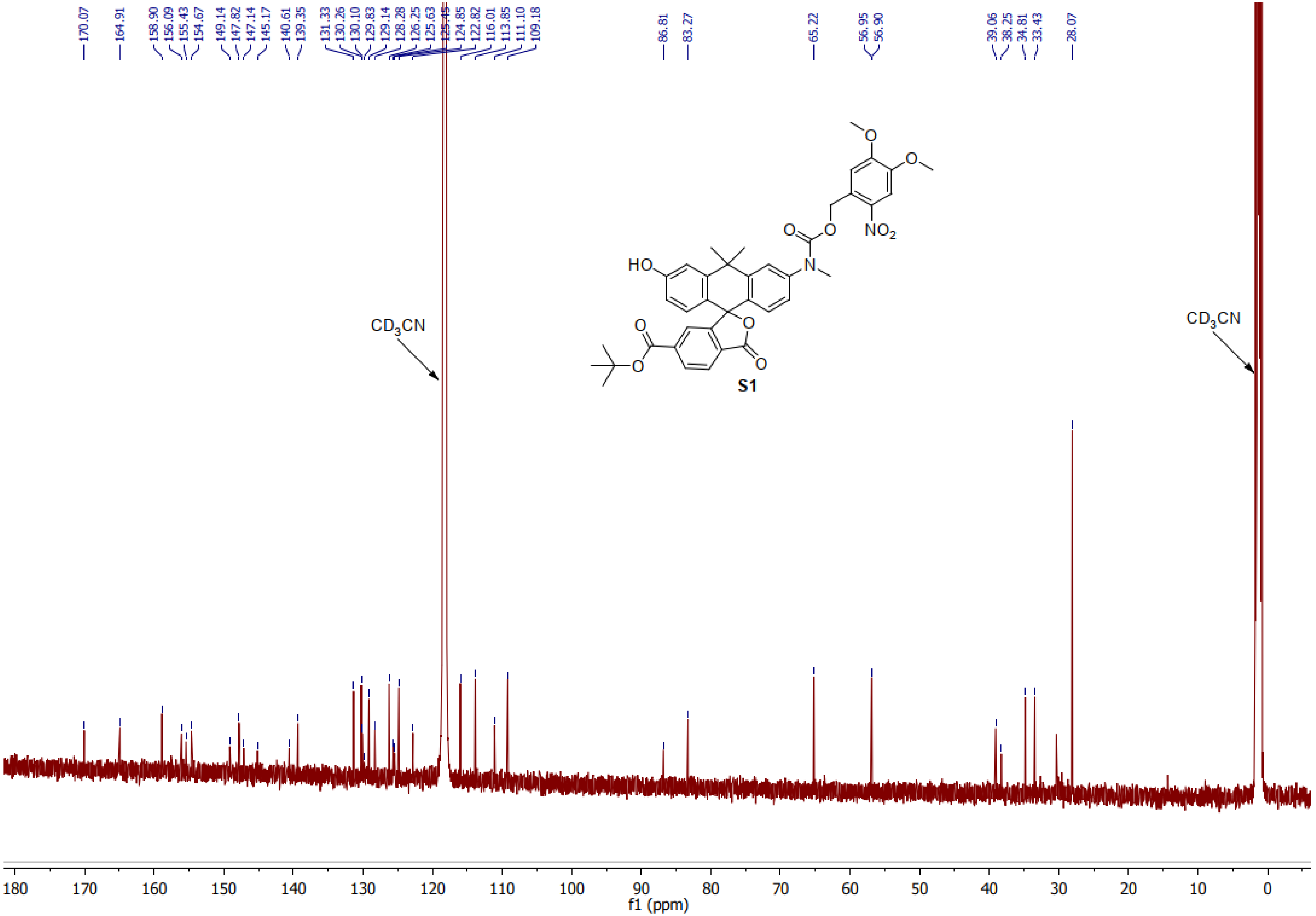
170.07
164.91
158.90
156.09
155.43
154.67
149.14
147.82
147.14
145.17
140.61
139.35
131.33
130.26
130.10
129.83
129.14
128.28
126.25
125.63
125.45
124.85
122.82
116.01
113.85
111.10
109.18
86.81
83.27
65.22
56.95
56.90
39.06
38.25
34.81
33.43
28.07
CD3CN
CD3CN
HO
O
O
O
O
N
O
O
O
O
NO2
S1
180
170
160
150
140
130
120
110
100
90
80
70
60
50
40
30
20
10
0
f1 (ppm)

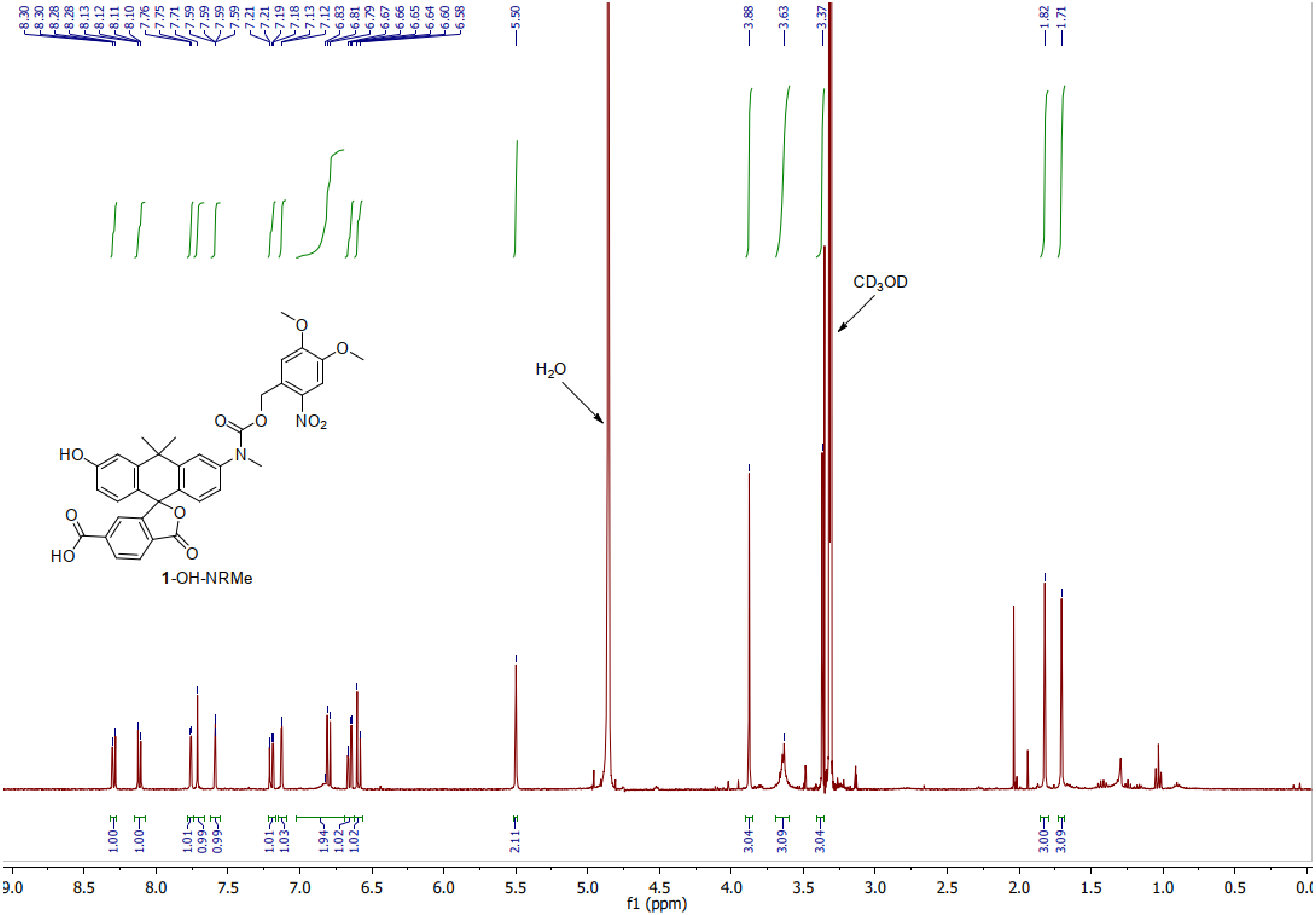

8.30 8.30 8.28 8.28 8.13 8.12 8.11 8.10 7.76 7.75 7.71 7.59 7.59 7.59 7.59 7.21 7.21 7.19 7.18 7.13 7.12 6.83 6.81 6.79 6.67 6.66 6.65 6.64 6.60 6.58
5.50
3.88
3.63
3.37
1.82
1.71
H2O
CD3OD
HO
O
N
O
O
NO2
O
O
HO
O
O
1-OH-NRMe
1.00
1.00
1.01
0.99
0.99
1.01
1.03
1.94
1.02
1.02
2.11
3.04
3.09
3.04
3.00
3.09
9.0 8.5 8.0 7.5 7.0 6.5 6.0 5.5 5.0 4.5 4.0 3.5 3.0 2.5 2.0 1.5 1.0 0.5 0.0
f1 (ppm)

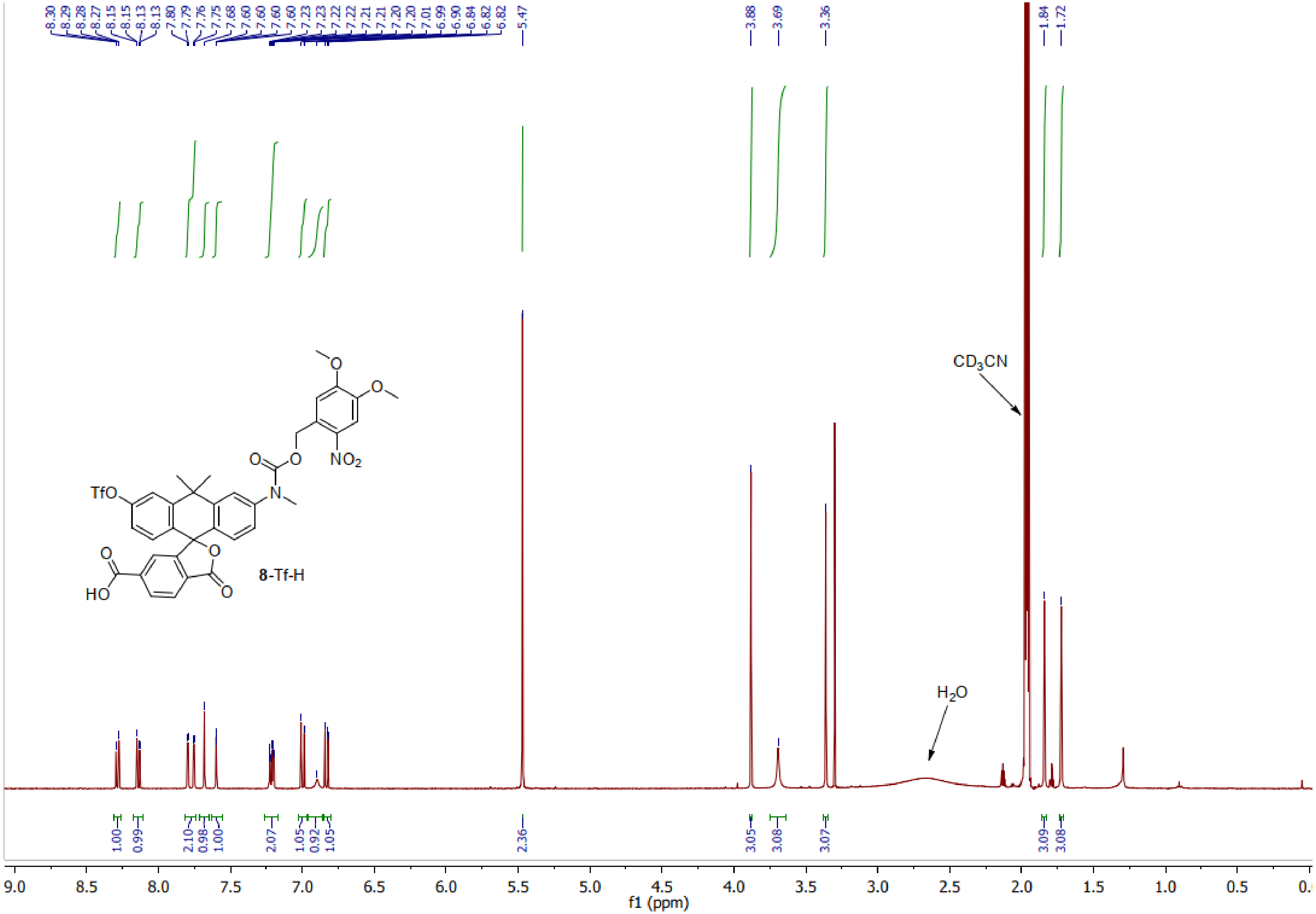
CD3CN
H2O
8-Tf-H
TfO
HO
NO2
f1 (ppm)

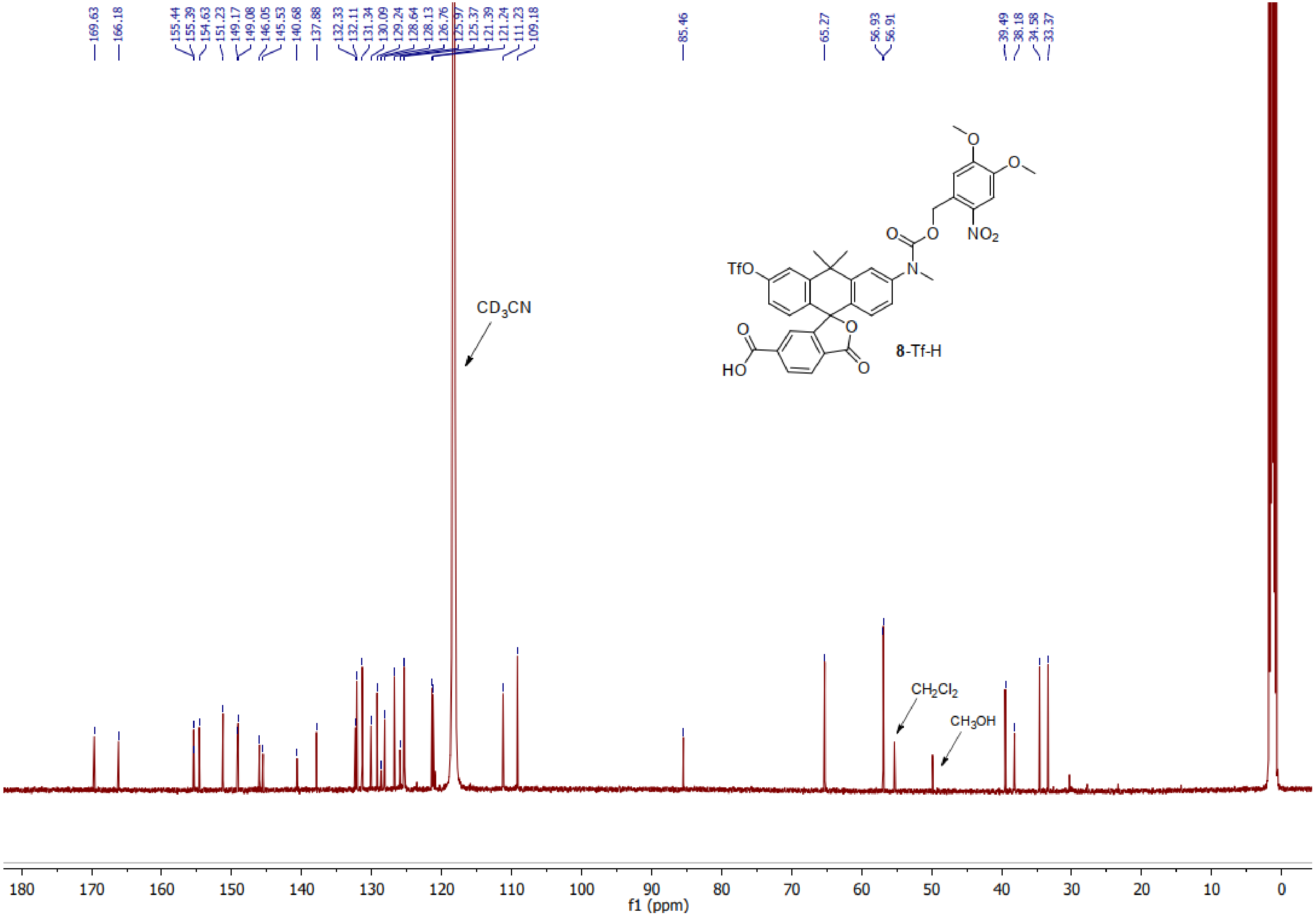
169.63
166.18
155.44
155.39
154.63
151.23
149.17
149.08
146.05
145.53
140.68
137.88
132.33
132.11
131.34
130.09
129.24
128.64
128.13
126.76
125.97
125.37
121.39
121.24
111.23
109.18
85.46
65.27
56.93
56.91
39.49
38.18
34.58
33.37
CD3CN
TfO
O
N
NO2
HO
8-Tf-H
CH2Cl2
CH3OH
f1 (ppm)

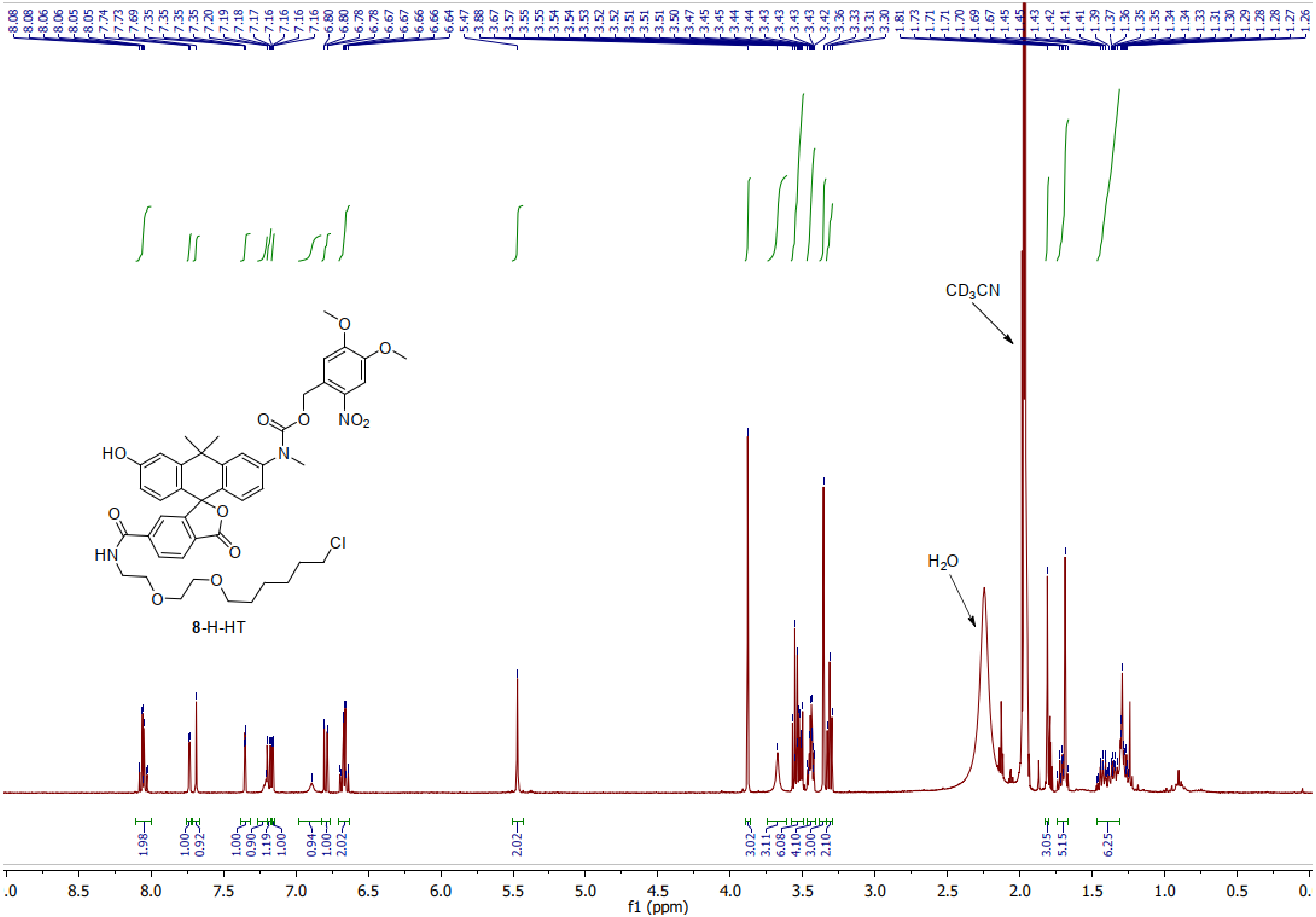
CD3CN
H2O
HO
O
NO2
N
Cl
HN
8-H-HT
f1 (ppm)

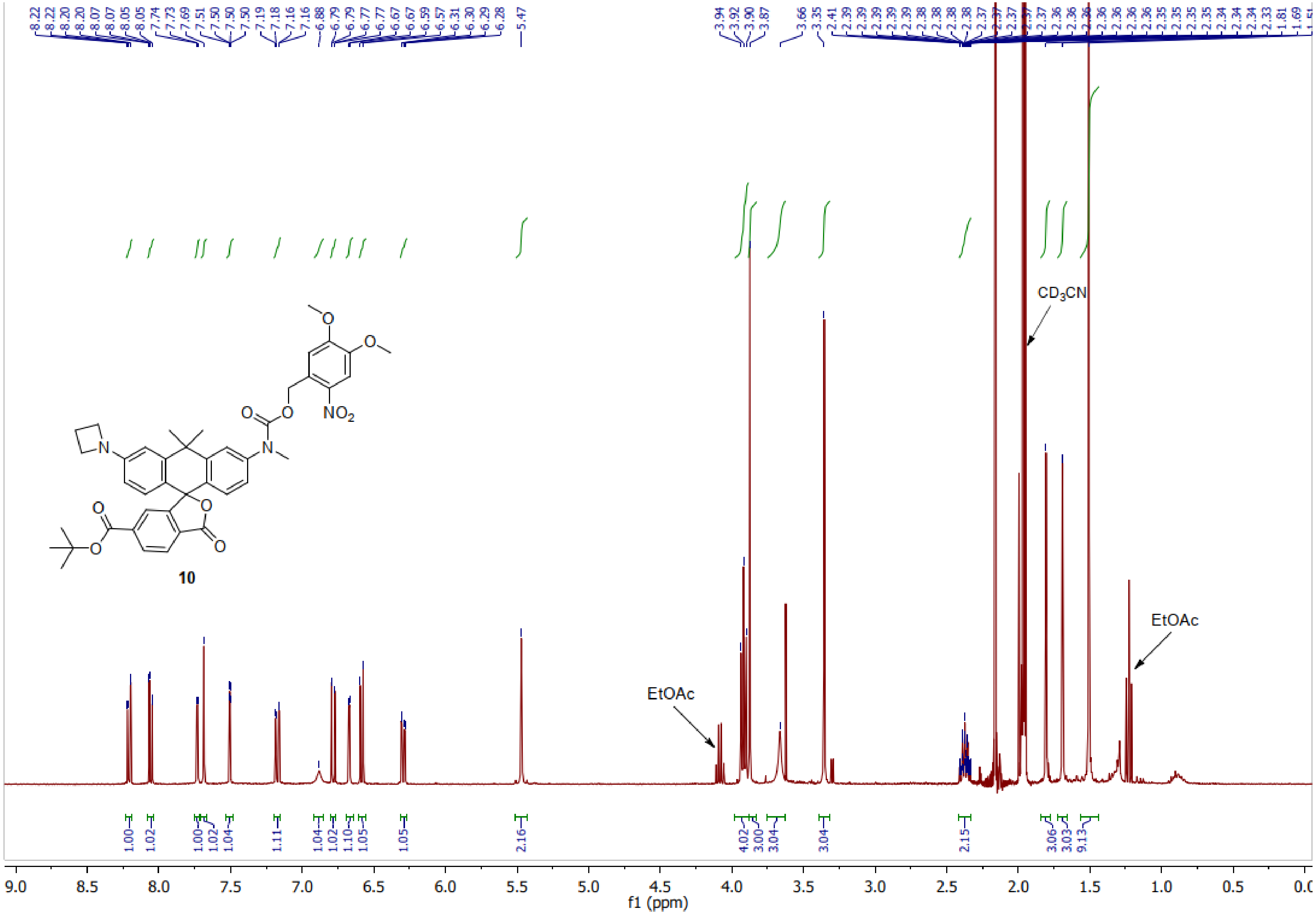
EtOAc
CD3CN
EtOAc
10
f1 (ppm)

^{13}C-NMR (126 MHz, CD_3CN) comp. **10** (x x x x – signals of residual solvents: ethyl acetate – 14, 21 and 60 ppm and dioxane – 67 ppm).

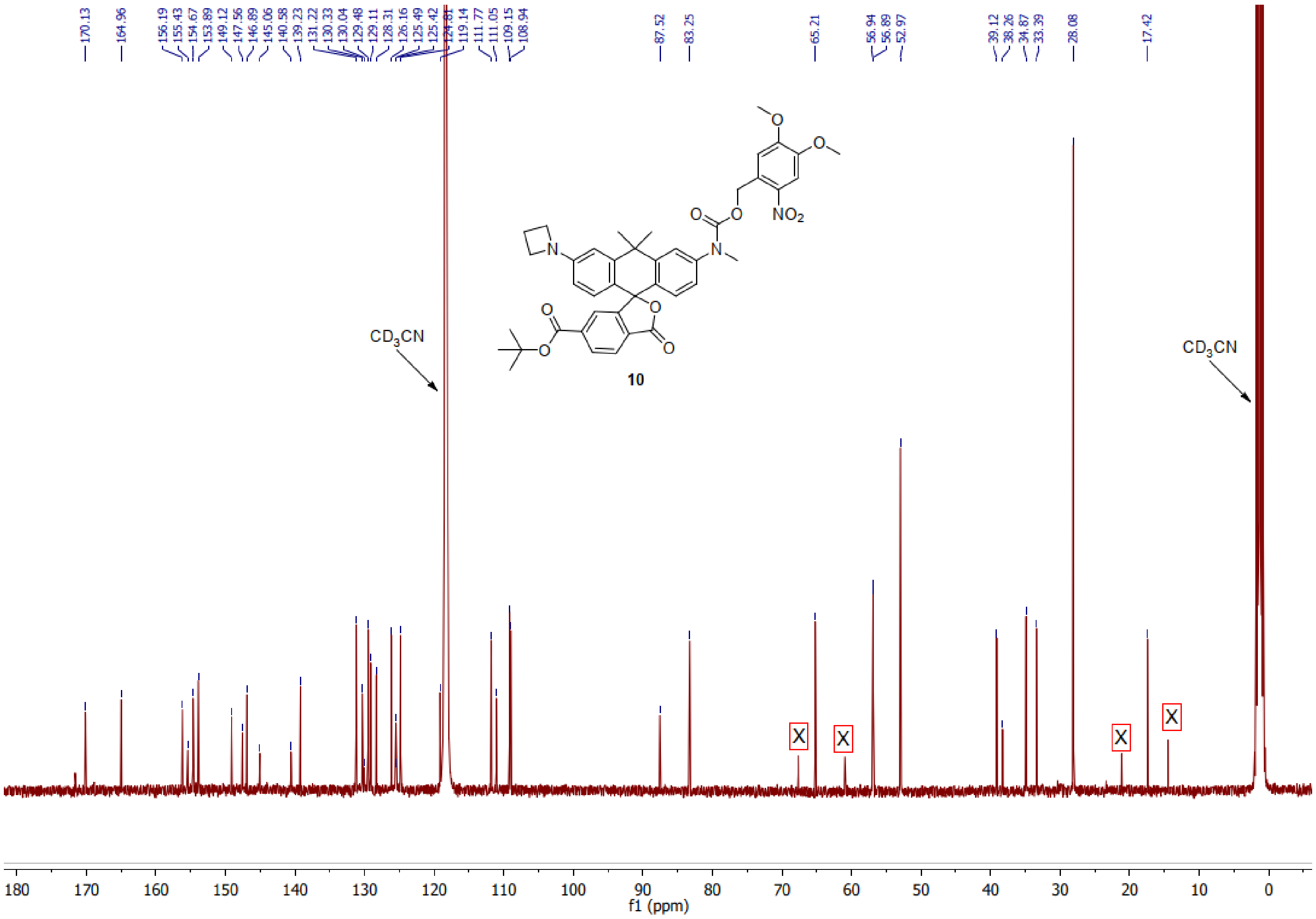

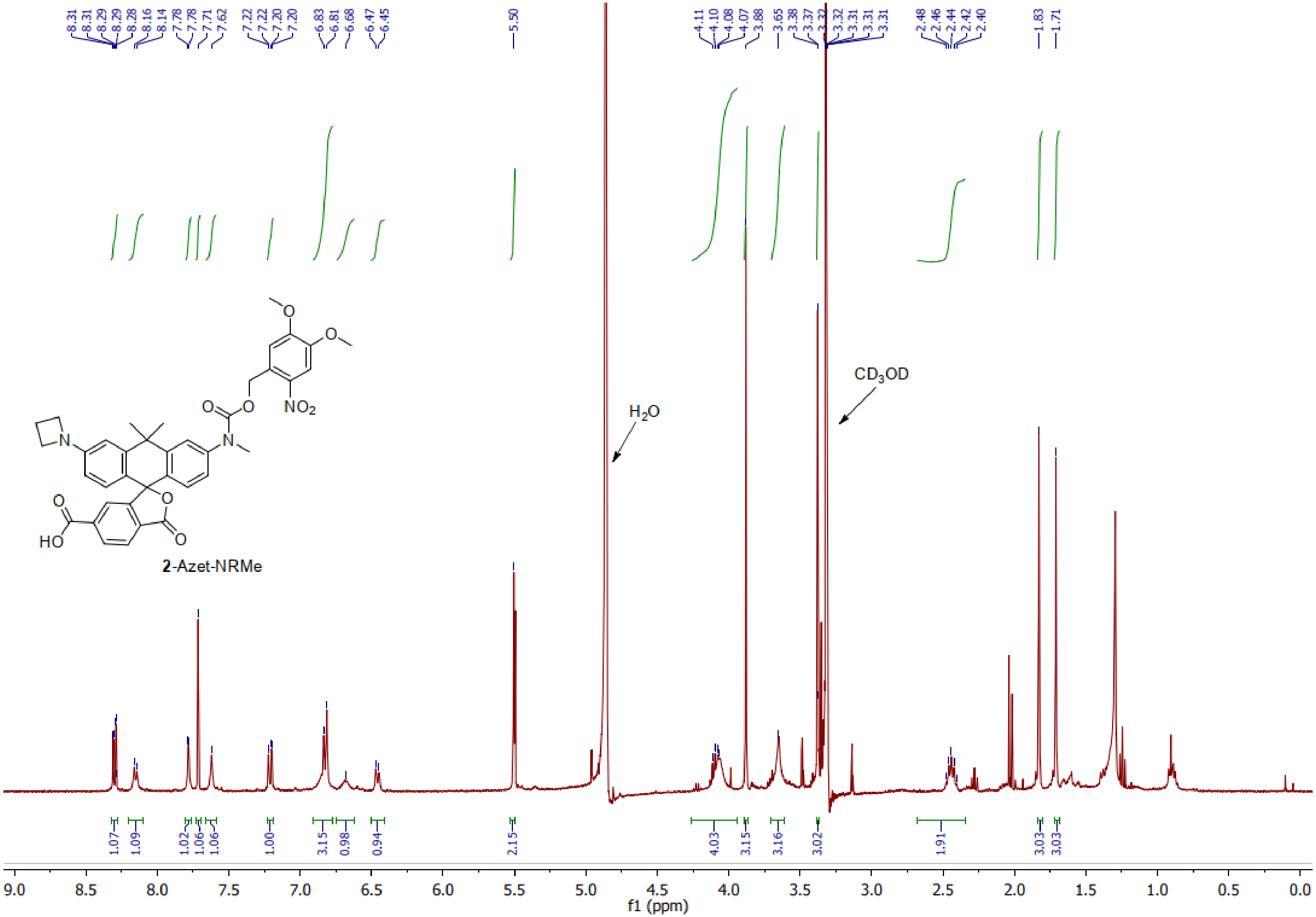

2-Azet-NRMe

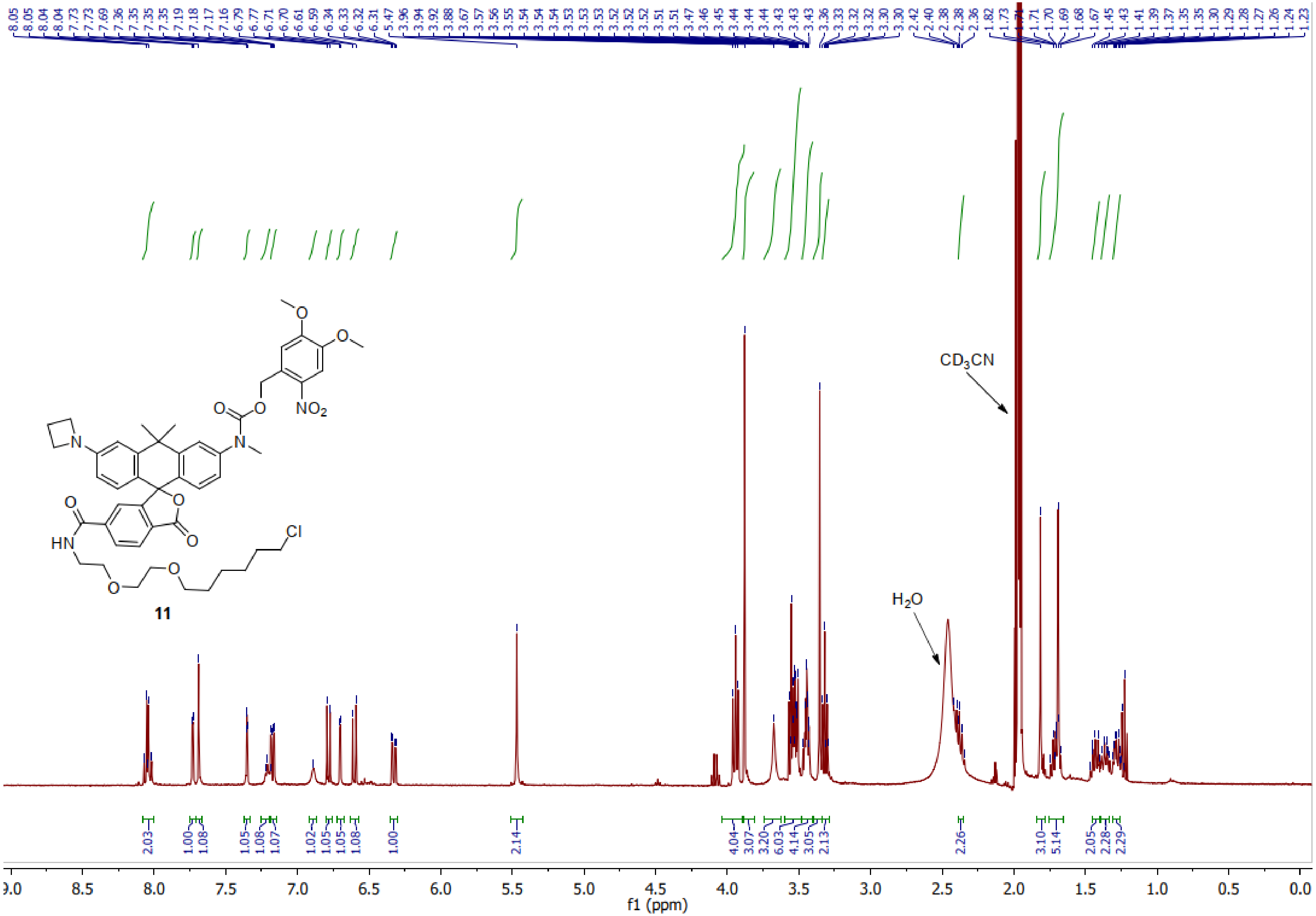
CD3CN
H2O
11
f1 (ppm)

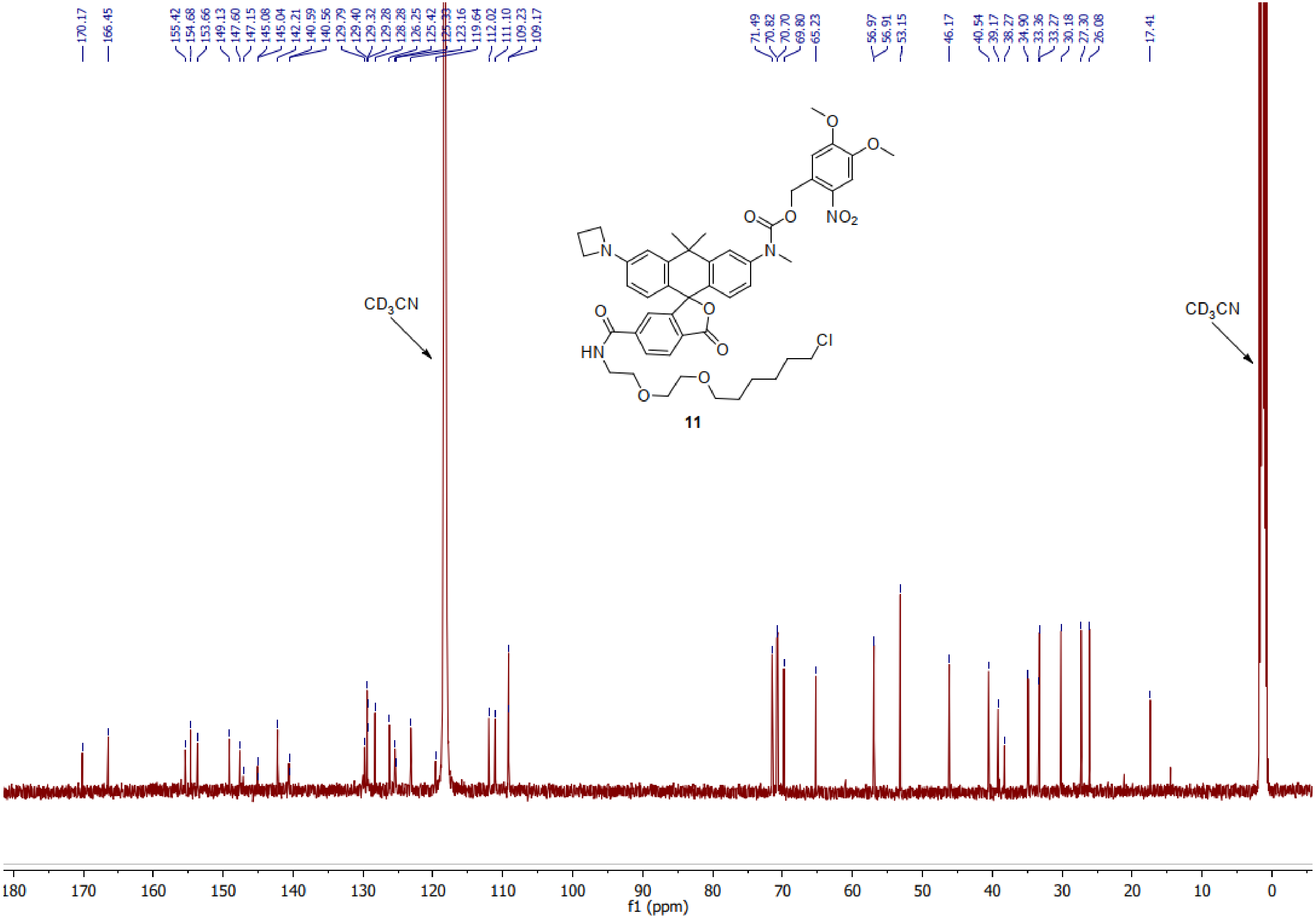
170.17
166.45
155.42
154.68
153.66
149.13
147.60
147.15
145.08
145.04
142.21
140.59
140.56
129.79
129.40
129.32
129.28
128.28
126.25
125.42
125.33
123.16
119.64
112.02
111.10
109.23
109.17
71.49
70.82
70.70
69.80
65.23
56.97
56.91
53.15
46.17
40.54
39.17
38.27
34.90
33.36
33.27
30.18
27.30
26.08
17.41
CD3CN
CD3CN
11
f1 (ppm)

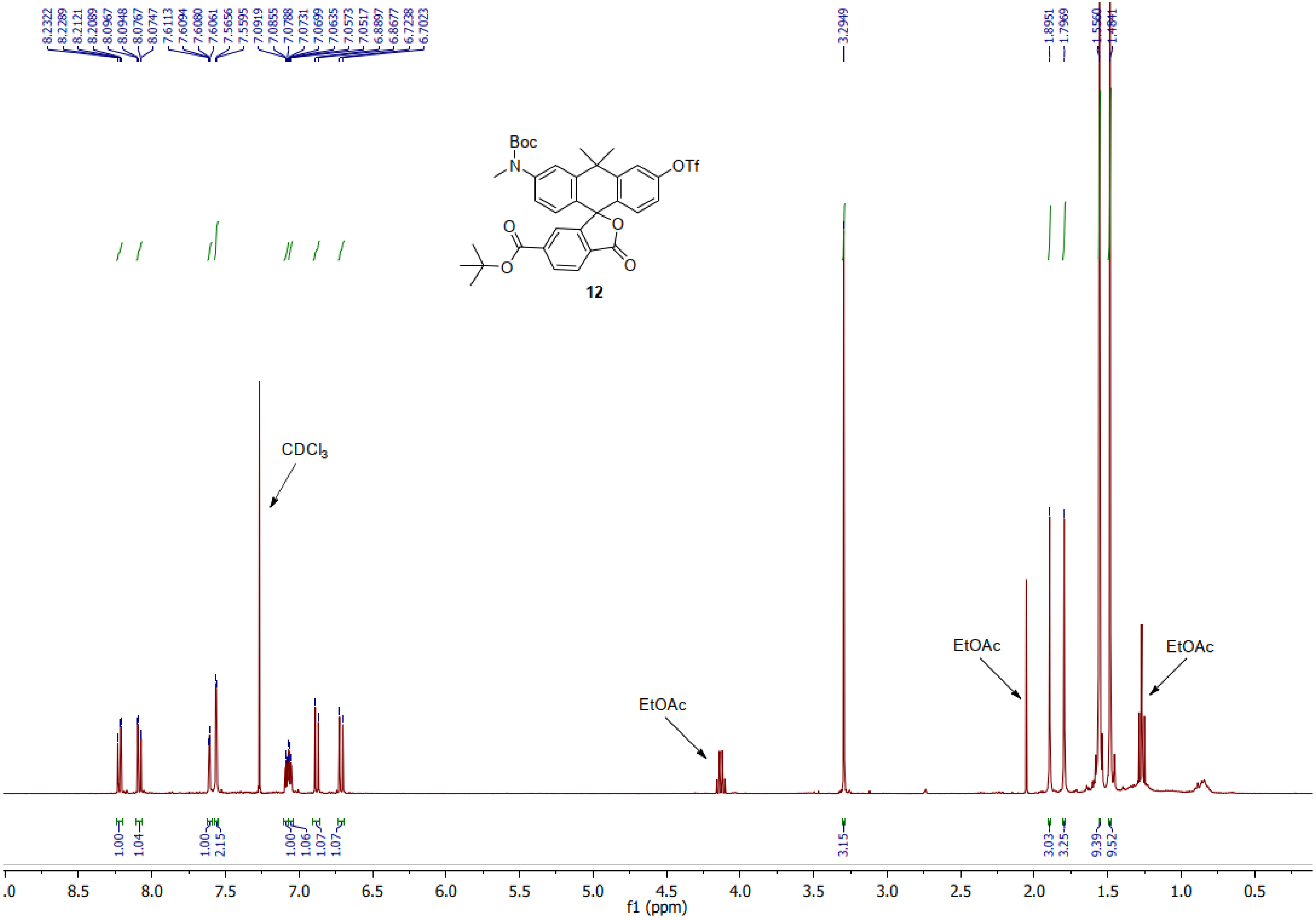

8.2322
8.2289
8.2121
8.2089
8.0967
8.0948
8.0767
8.0747
7.6113
7.6094
7.6080
7.6061
7.5656
7.5595
7.0919
7.0855
7.0788
7.0731
7.0699
7.0635
7.0573
7.0517
6.8897
6.8677
6.7238
6.7023
3.2949
1.8951
1.7969
1.5560
1.4841
Boc
N
OTf
O
O
O
O
12
CDCl3
EtOAc
EtOAc
EtOAc
1.00
1.04
1.00
2.15
1.00
1.06
1.07
1.07
3.15
3.03
3.25
9.39
9.52
.0
8.5
8.0
7.5
7.0
6.5
6.0
5.5
5.0
4.5
4.0
3.5
3.0
2.5
2.0
1.5
1.0
0.5
f1 (ppm)

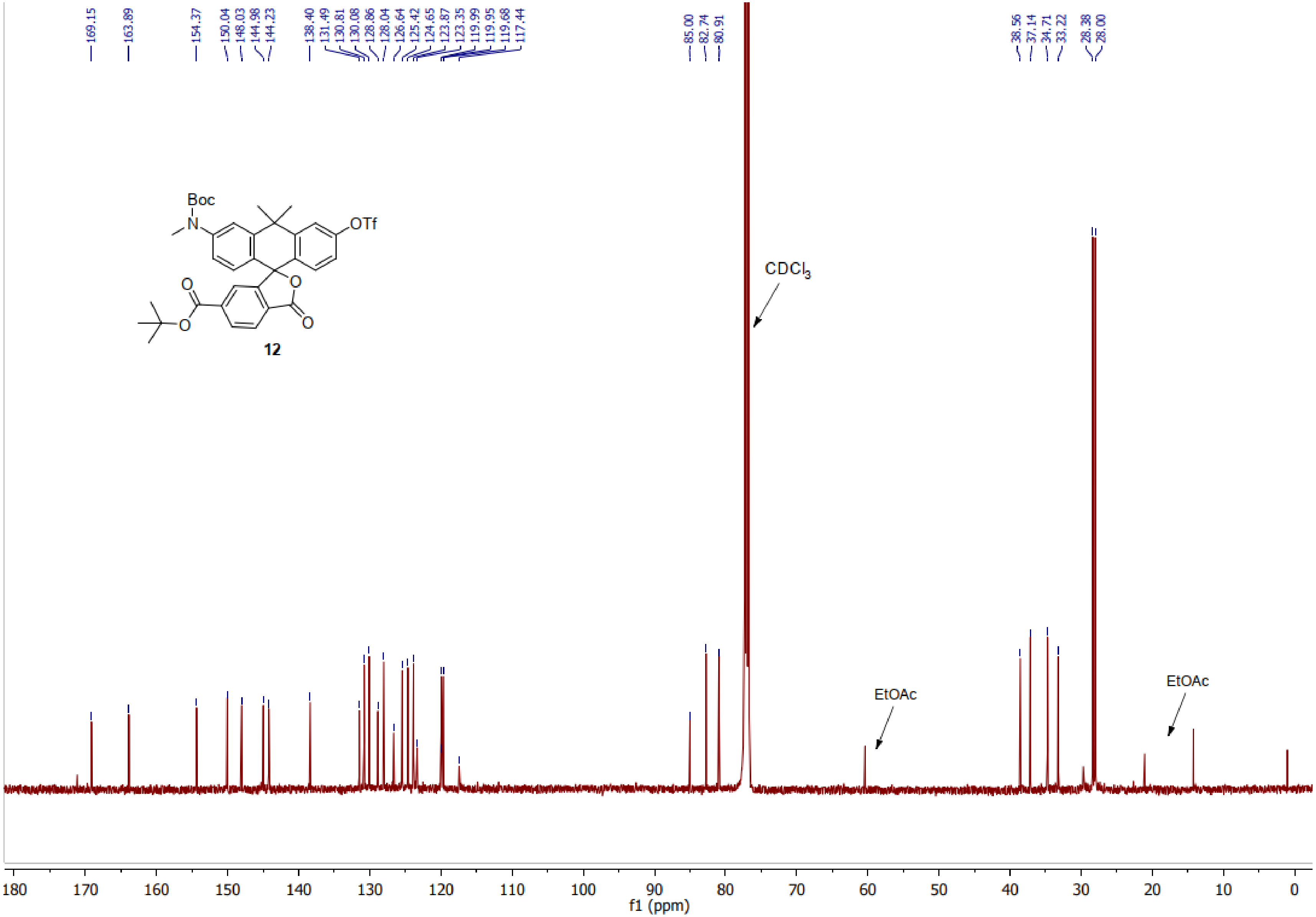

169.15
163.89
154.37
150.04
148.03
144.98
144.23
138.40
131.49
130.81
130.08
128.86
128.04
126.64
125.42
124.65
123.87
123.35
119.99
119.95
119.68
117.44
85.00
82.74
80.91
38.56
37.14
34.71
33.22
28.38
28.00
Boc
N
OTf
O
O
O
O
12
CDCl3
EtOAc
EtOAc
f1 (ppm)

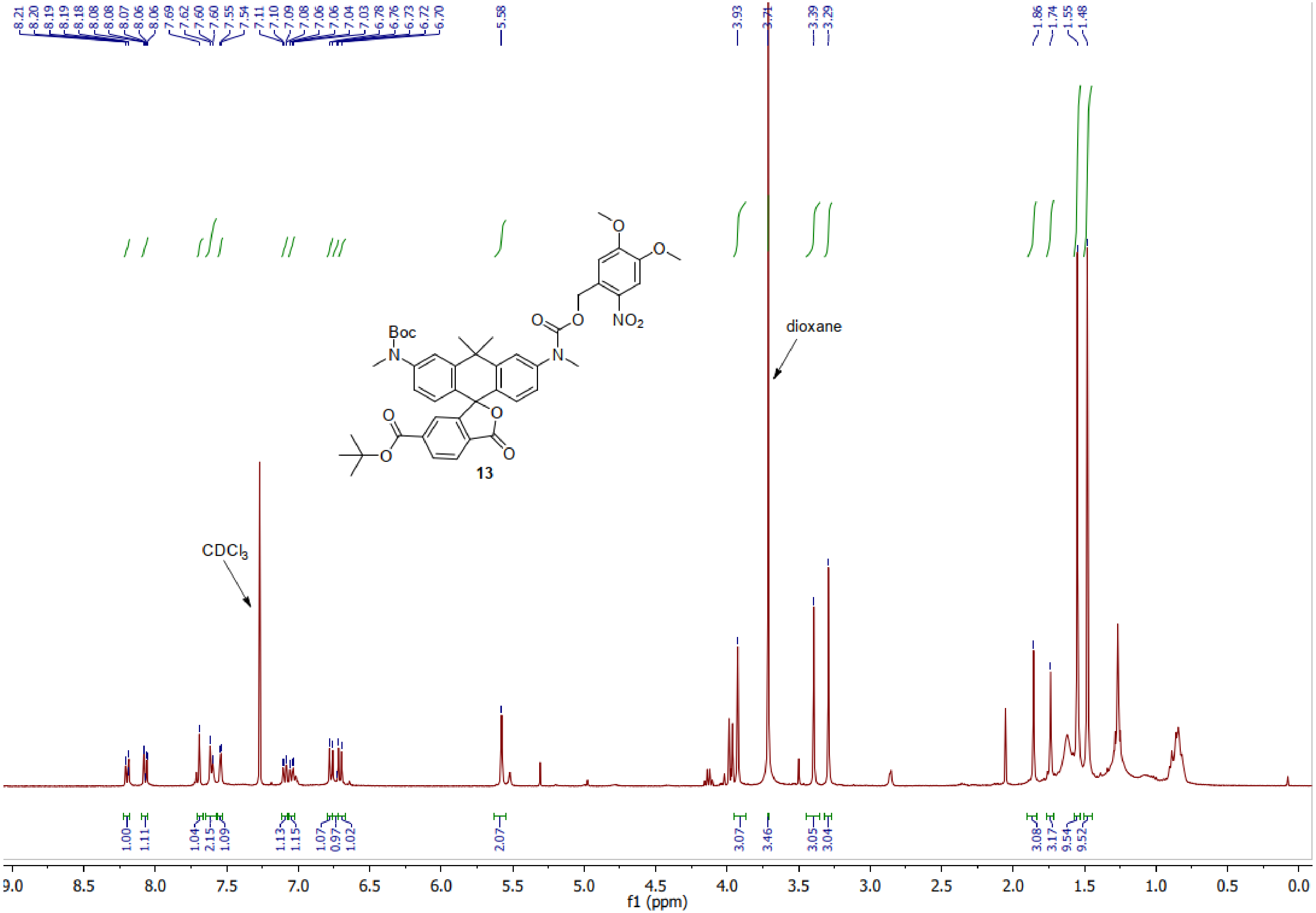
Boc
NO2
13
dioxane
CDCl3
f1 (ppm)

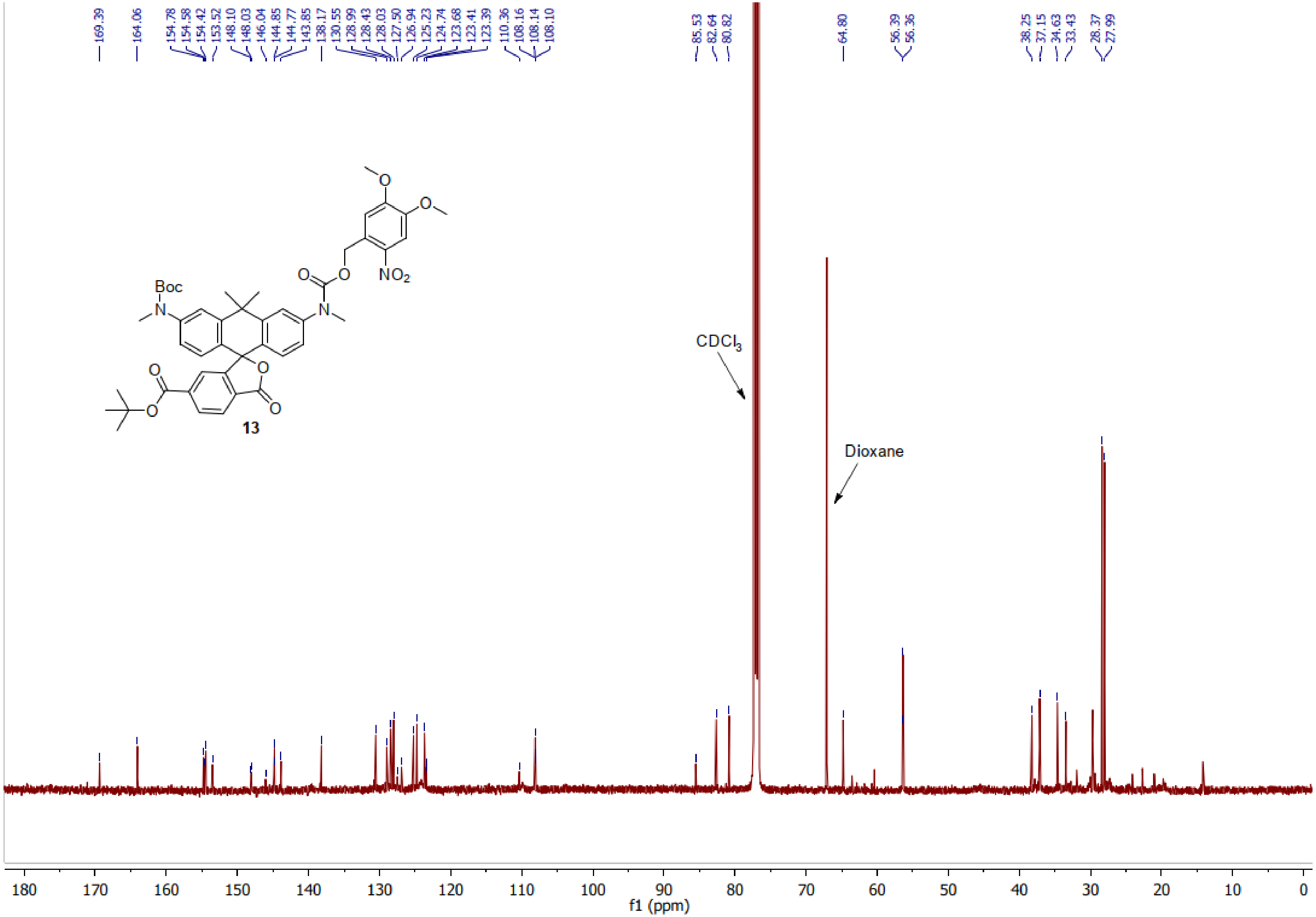
169.39
164.06
154.78
154.58
154.42
153.52
148.10
148.03
146.04
144.85
144.77
143.85
138.17
130.55
128.99
128.43
128.03
127.50
126.94
125.23
124.74
123.68
123.41
123.39
110.36
108.16
108.14
108.10
85.53
82.64
80.82
64.80
56.39
56.36
38.25
37.15
34.63
33.43
28.37
27.99
Boc
N
O
O
NO2
13
CDCl3
Dioxane
f1 (ppm)

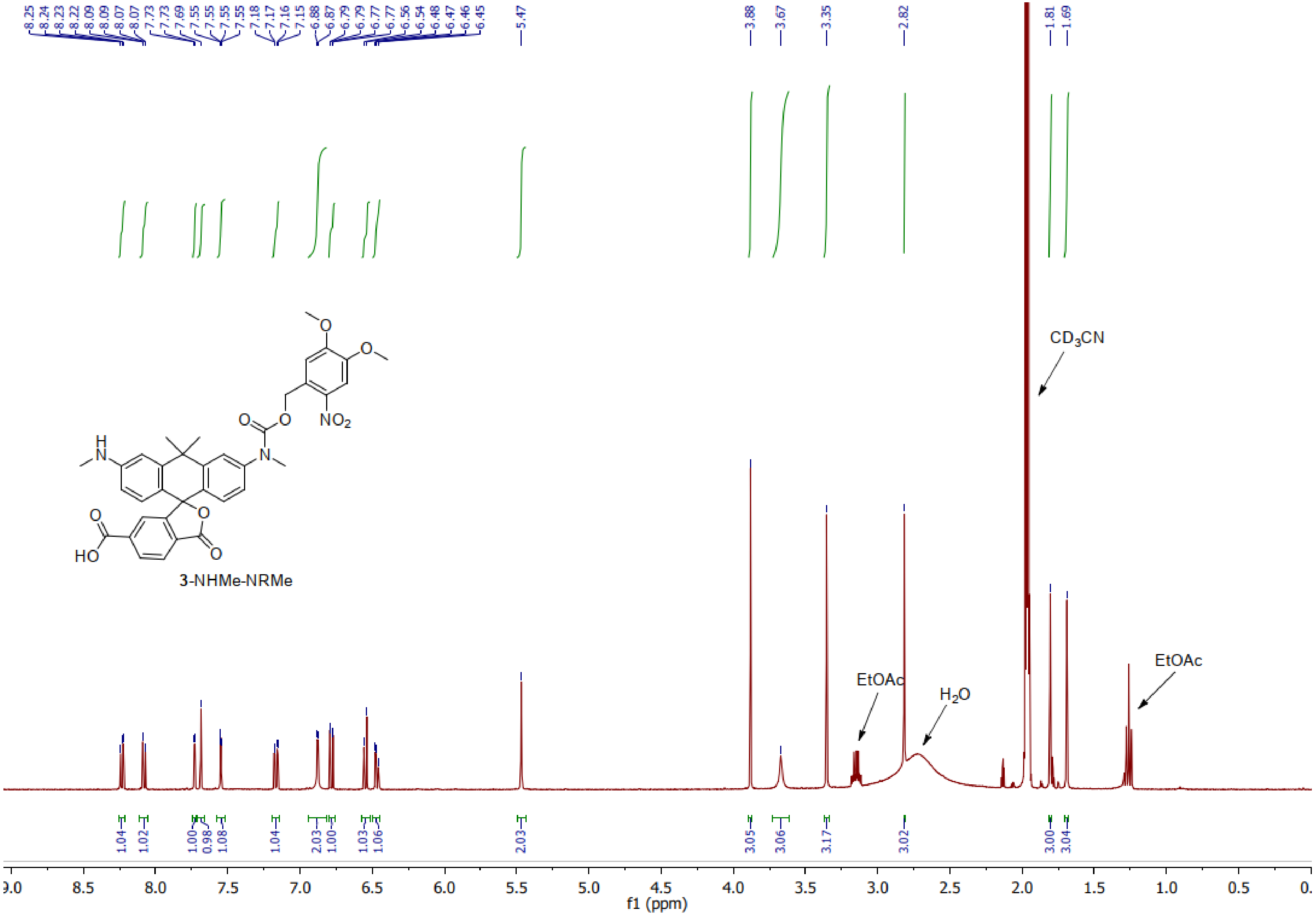
3-NHMe-NRMe
CD3CN
EtOAc
H2O
EtOAc
f1 (ppm)

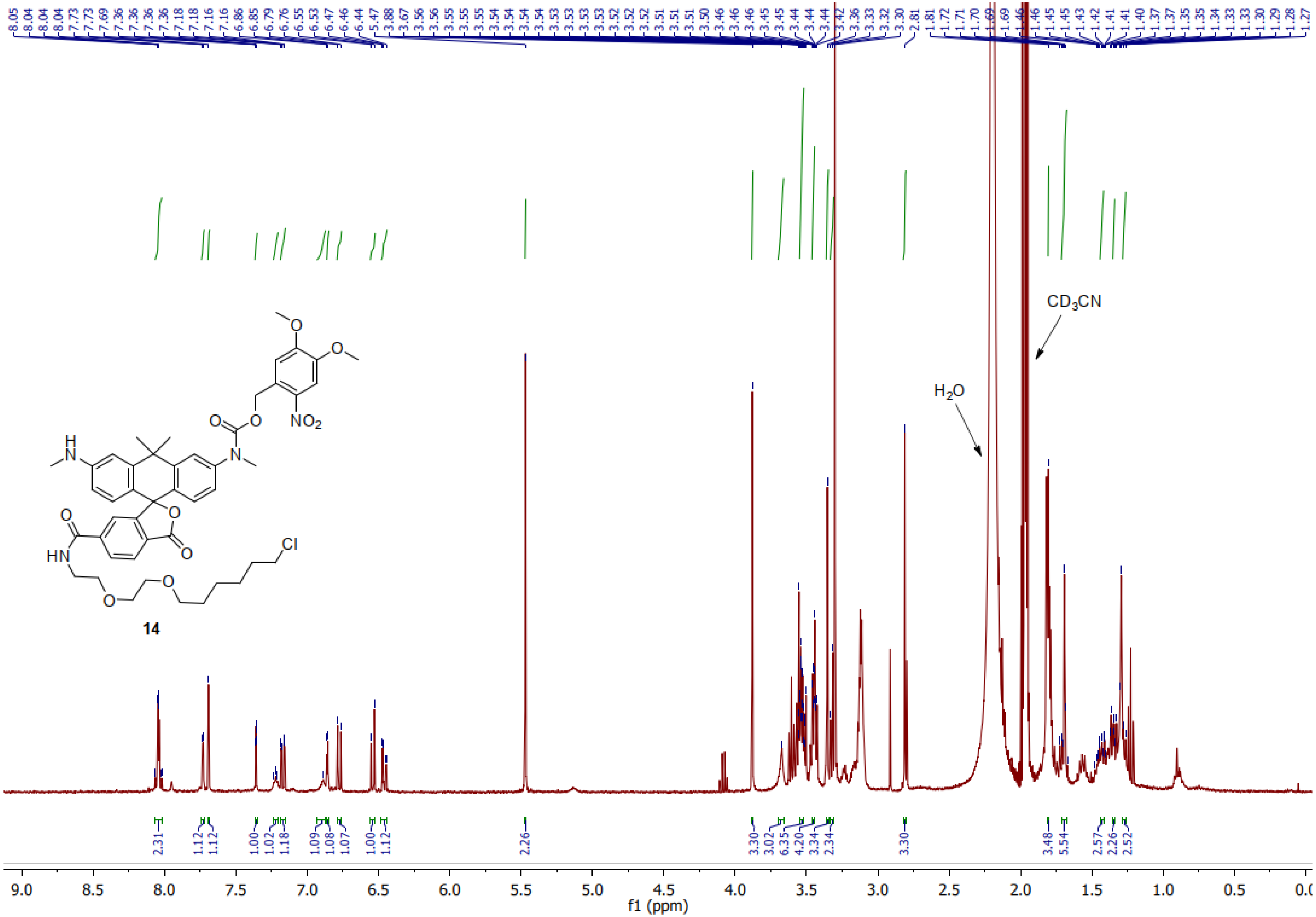
CD3CN
H2O
NO2
Cl
14
f1 (ppm)

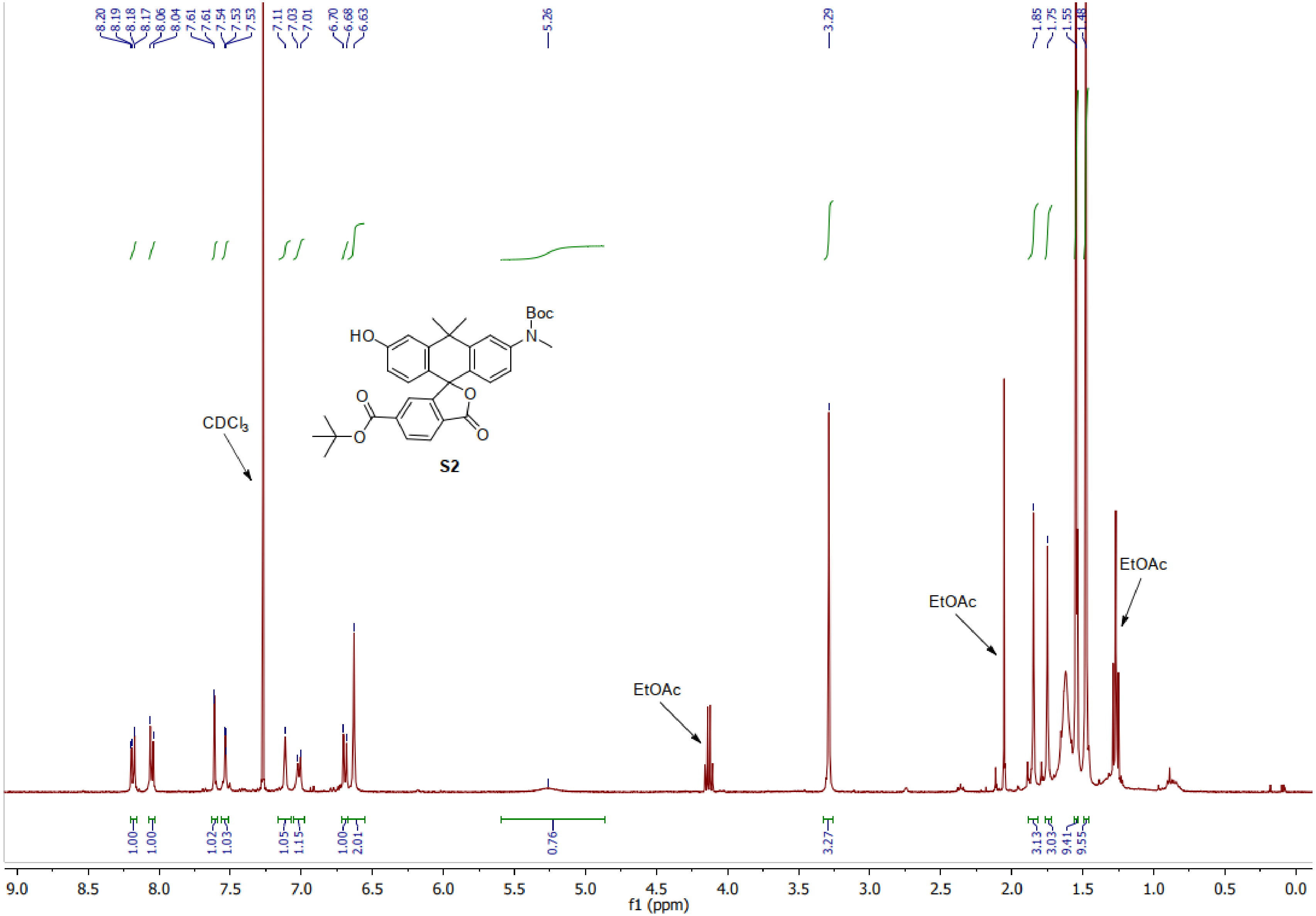
8.20 8.19 8.18 8.17 8.06 8.04 7.61 7.61 7.54 7.53 7.53 7.11 7.03 7.01 6.70 6.68 6.63 5.26 3.29 1.85 1.75 1.55 1.48
CDCl3
EtOAc
EtOAc
EtOAc
HO
Boc
N
O
O
O
O
S2
1.00 1.00 1.02 1.03 1.05 1.15 1.00 2.01 0.76 3.27 3.13 3.03 9.41 9.55
9.0 8.5 8.0 7.5 7.0 6.5 6.0 5.5 5.0 4.5 4.0 3.5 3.0 2.5 2.0 1.5 1.0 0.5 0.0
f1 (ppm)

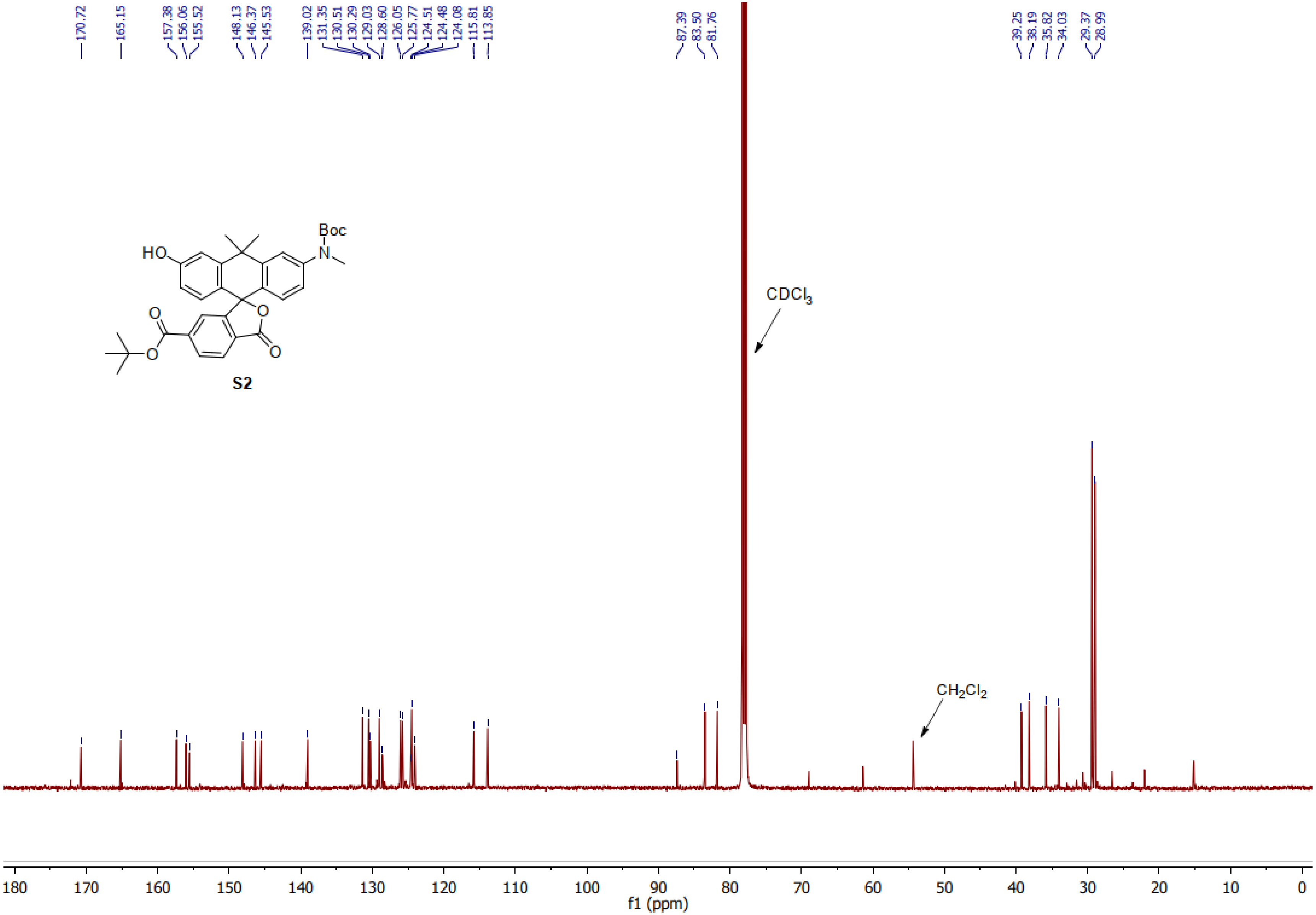
170.72
165.15
157.38
156.06
155.52
148.13
146.37
145.53
139.02
131.35
130.51
130.29
129.03
128.60
126.05
125.77
124.51
124.48
124.08
115.81
113.85
87.39
83.50
81.76
39.25
38.19
35.82
34.03
29.37
28.99
HO
Boc
N
O
S2
CDCl3
CH2Cl2
180 170 160 150 140 130 120 110 100 90 80 70 60 50 40 30 20 10 0
f1 (ppm)

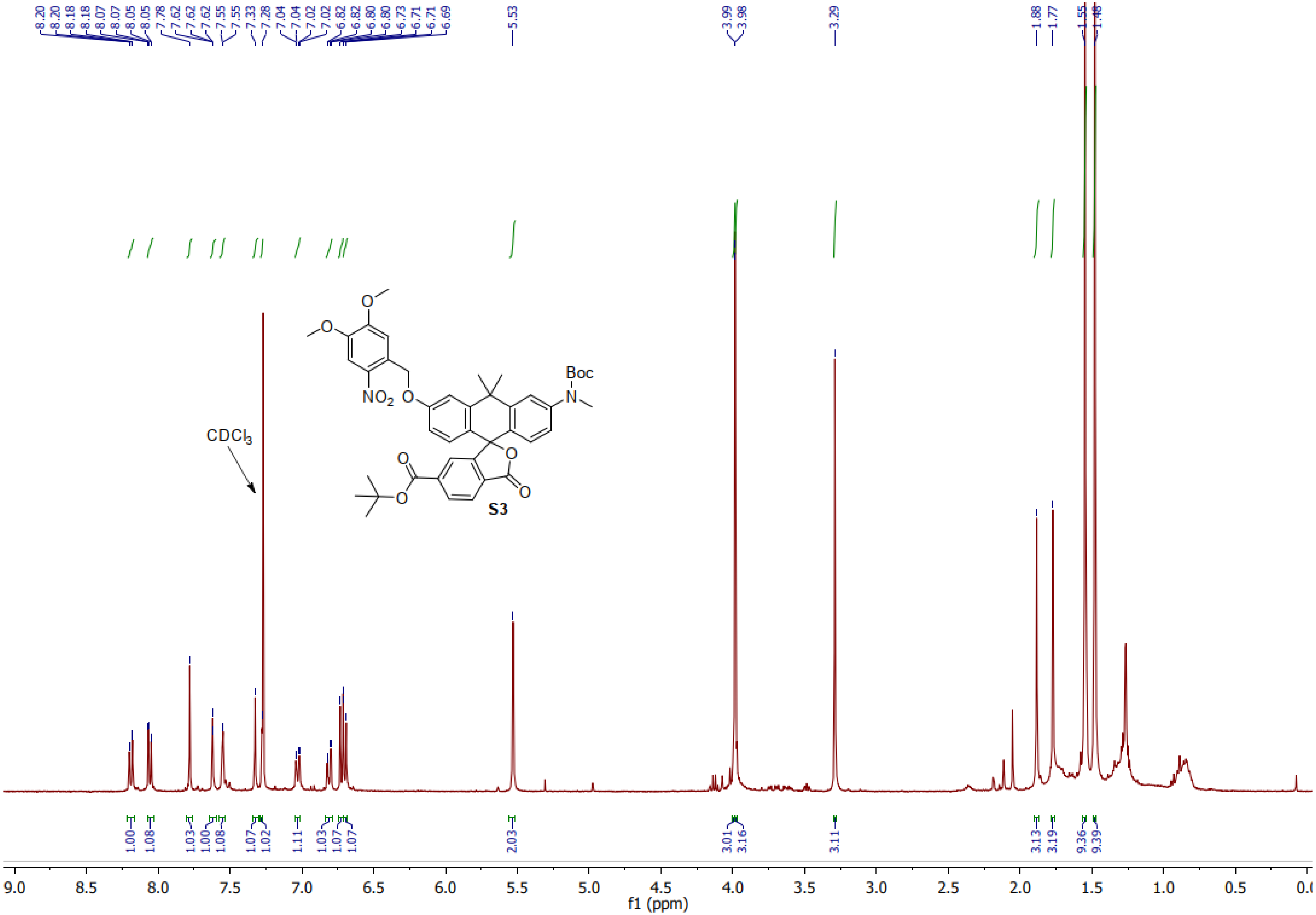
CDCl3
Boc
N
O
NO2
S3
f1 (ppm)

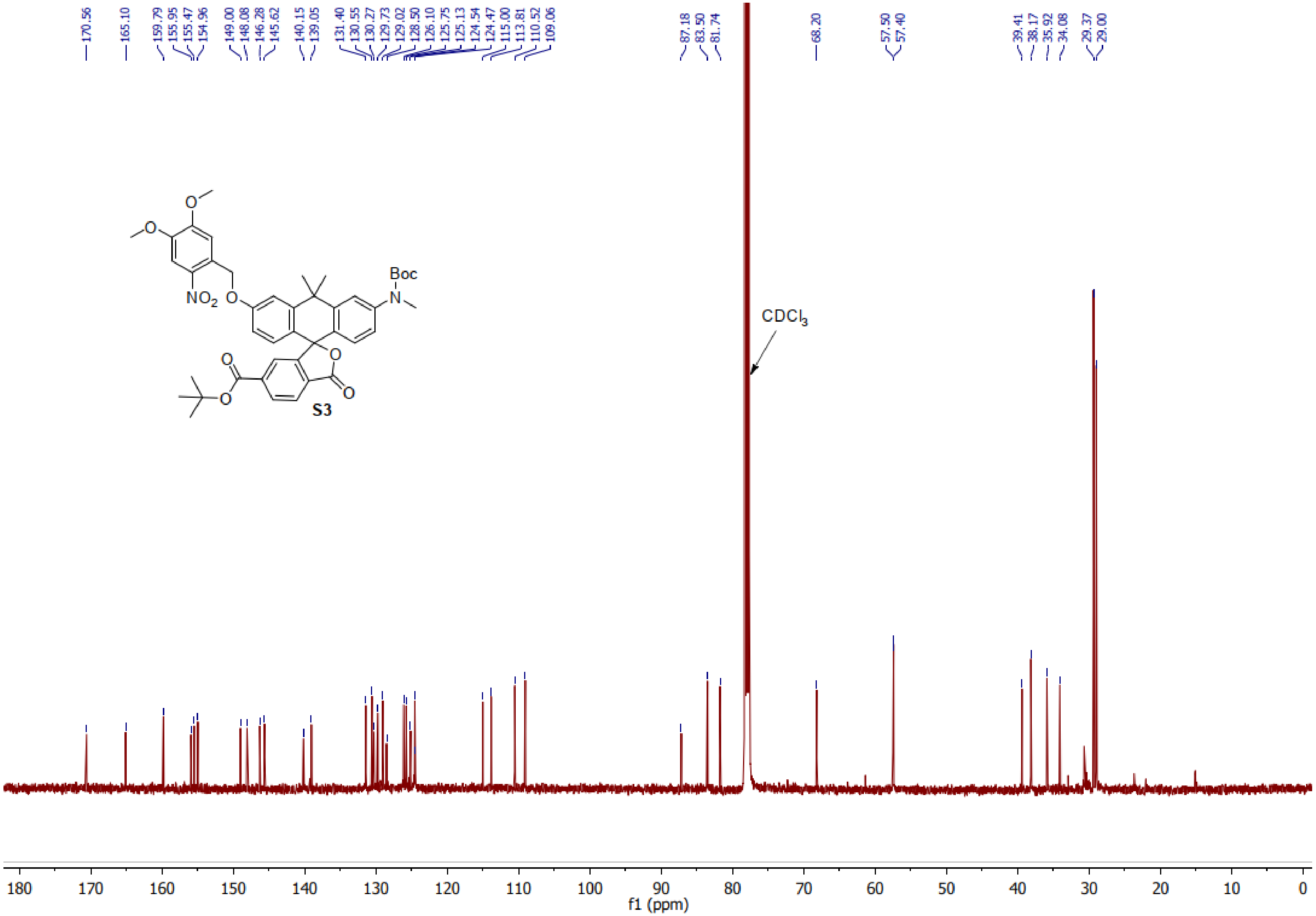
170.56
165.10
159.79
155.95
155.47
154.96
149.00
148.08
146.28
145.62
140.15
139.05
131.40
130.55
130.27
129.73
129.02
128.50
126.10
125.75
125.13
124.54
124.47
115.00
113.81
110.52
109.06
87.18
83.50
81.74
68.20
57.50
57.40
39.41
38.17
35.92
34.08
29.37
29.00
Boc
NO_2
S3
$CDCl_3$
180 170 160 150 140 130 120 110 100 90 80 70 60 50 40 30 20 10 0
f1 (ppm)

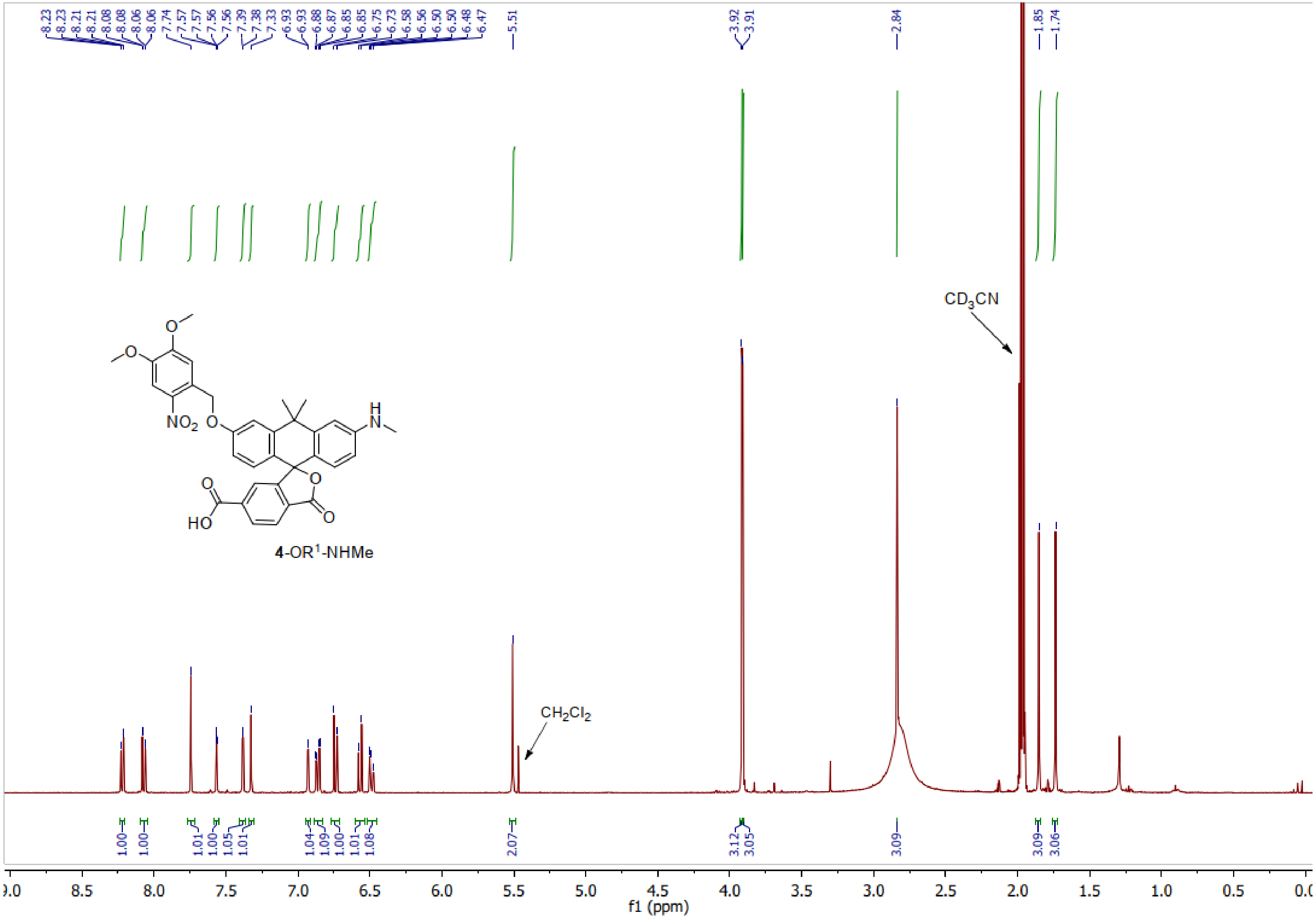
CD3CN
CH2Cl2
4-OR1-NHMe
f1 (ppm)

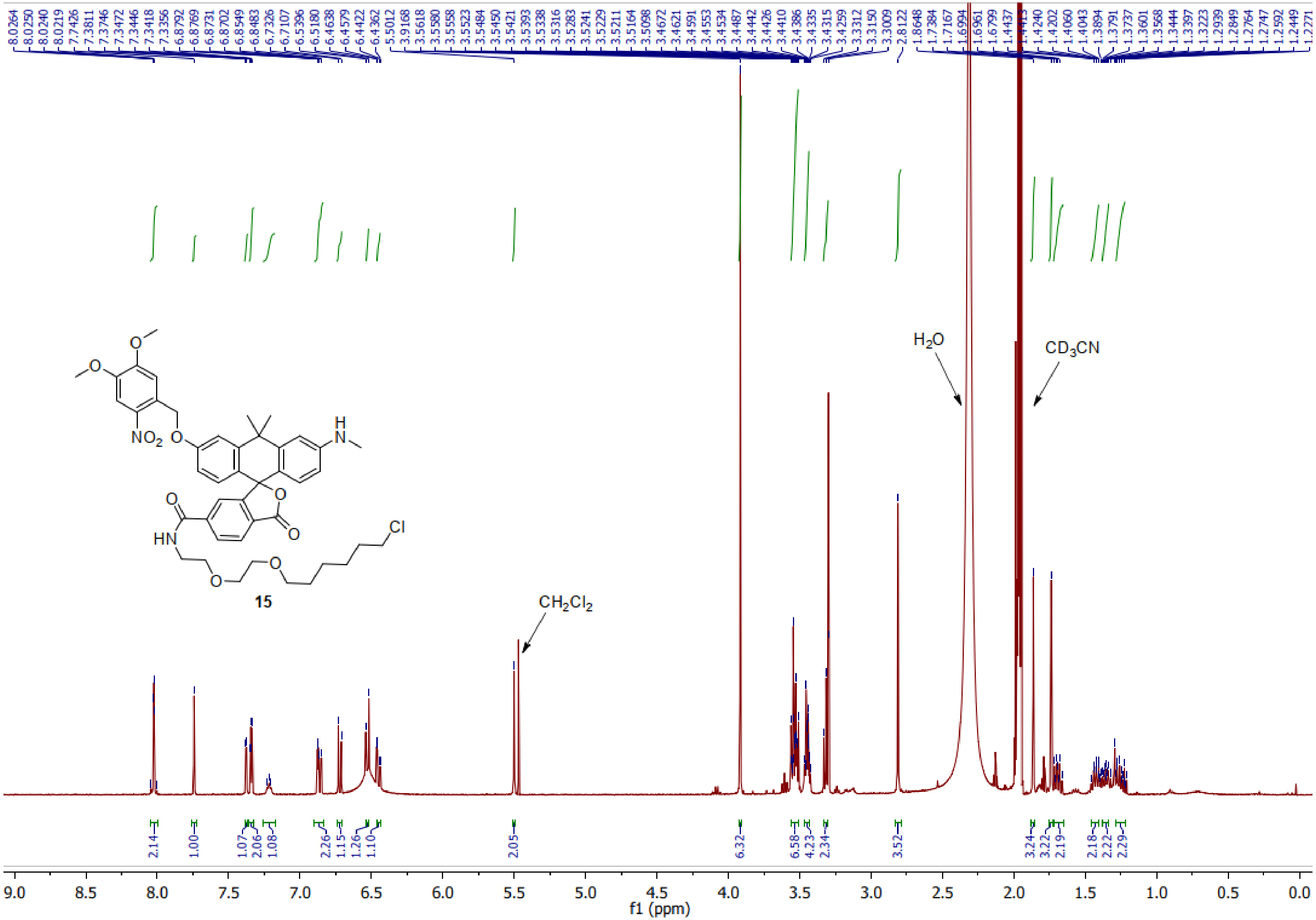

H2O
CD3CN
CH2Cl2
15
f1 (ppm)

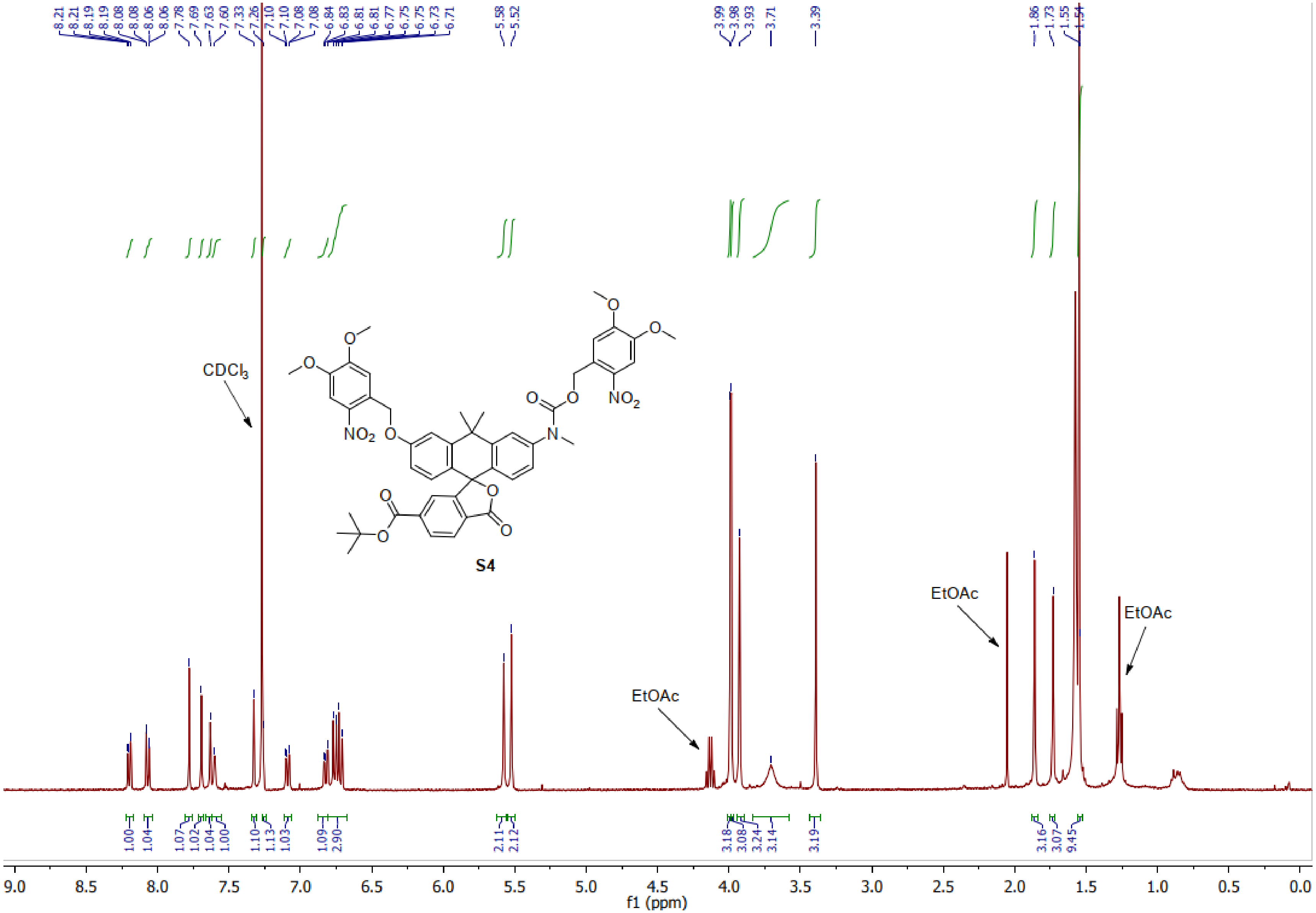

8.21
8.21
8.19
8.19
8.08
8.08
8.06
8.06
7.78
7.69
7.63
7.60
7.33
7.26
7.10
7.10
7.08
7.08
6.84
6.83
6.81
6.81
6.77
6.75
6.75
6.73
6.71
5.58
5.52
3.99
3.98
3.93
3.71
3.39
1.86
1.73
1.55
1.54
CDCl3
NO2
O
N
S4
EtOAc
EtOAc
EtOAc
1.00
1.04
1.07
1.02
1.04
1.00
1.10
1.13
1.03
1.09
2.90
2.11
2.12
3.18
3.08
3.24
3.14
3.19
3.16
3.07
9.45
9.0
8.5
8.0
7.5
7.0
6.5
6.0
5.5
5.0
4.5
4.0
3.5
3.0
2.5
2.0
1.5
1.0
0.5
0.0
f1 (ppm)

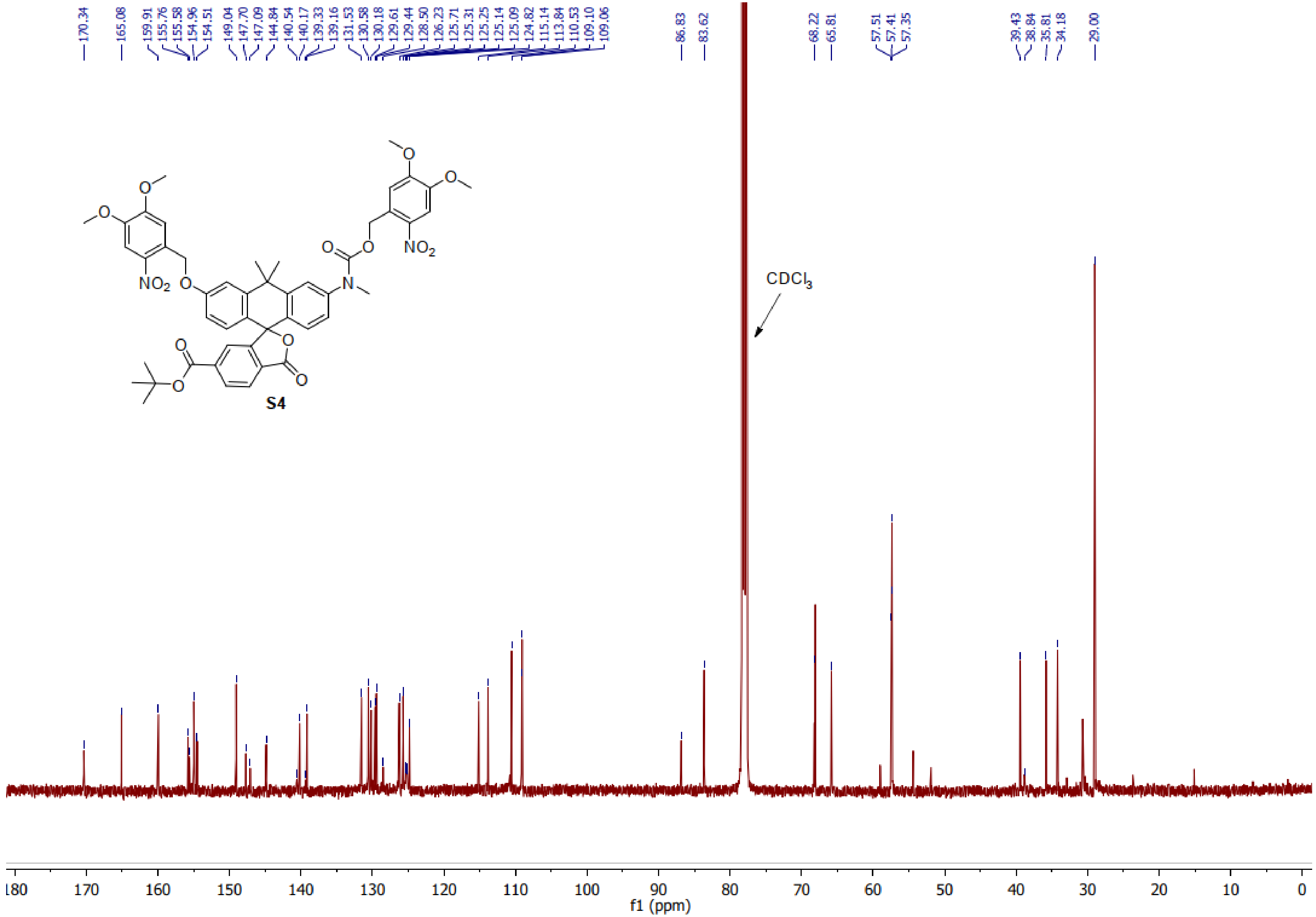

170.34
165.08
159.91
155.76
155.58
154.96
154.51
149.04
147.70
147.09
144.84
140.54
140.17
139.33
139.16
131.53
130.58
130.18
129.61
129.44
128.50
126.23
125.71
125.31
125.25
125.14
125.09
124.82
115.14
113.84
110.53
109.10
109.06
86.83
83.62
68.22
65.81
57.51
57.41
57.35
39.43
38.84
35.81
34.18
29.00
$CDCl_3$
S4
180 170 160 150 140 130 120 110 100 90 80 70 60 50 40 30 20 10 0
f1 (ppm)

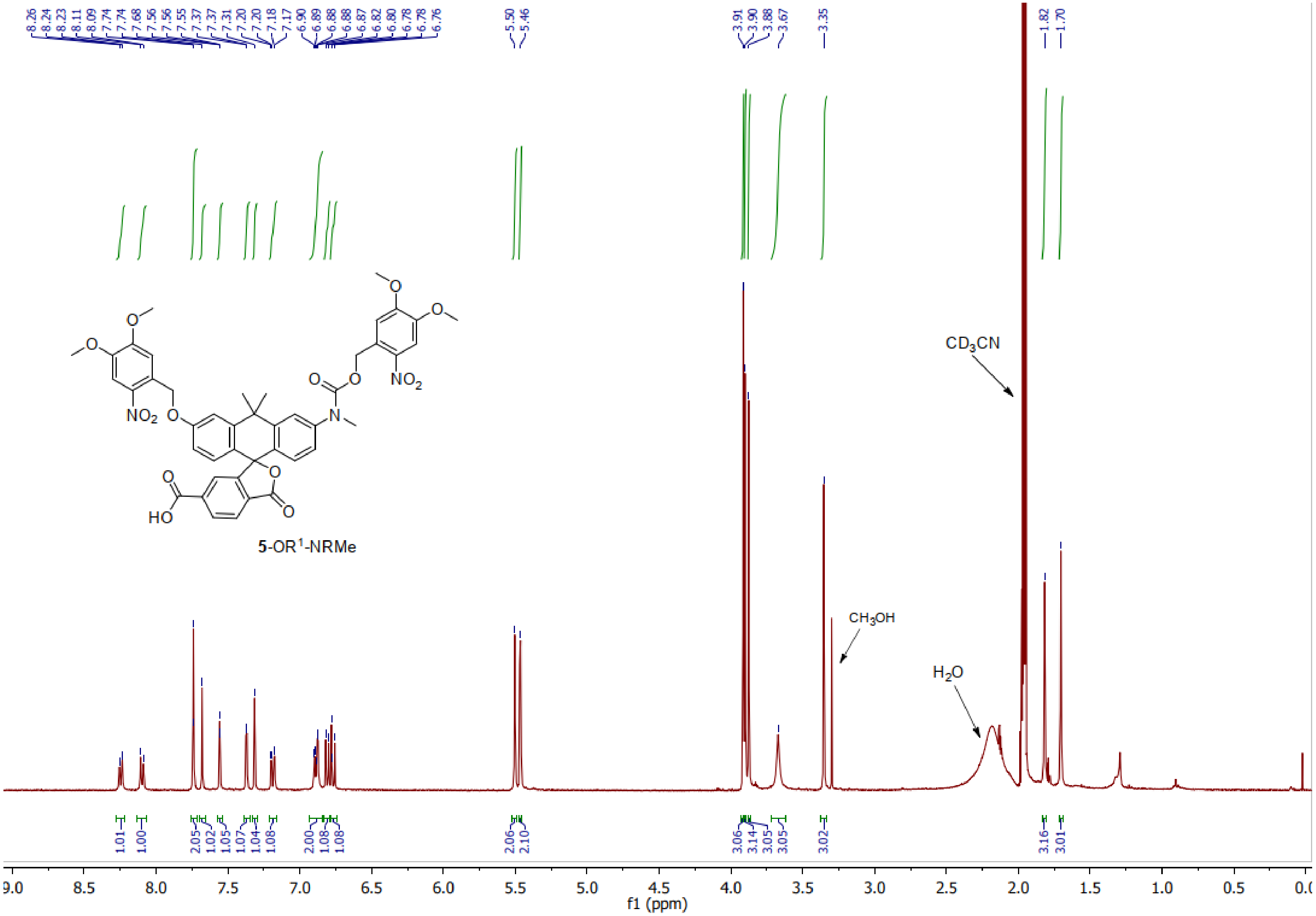
8.26
8.24
8.23
8.11
8.09
7.74
7.74
7.68
7.56
7.56
7.55
7.37
7.37
7.31
7.20
7.20
7.18
7.17
6.90
6.89
6.88
6.88
6.87
6.82
6.80
6.78
6.76
5.50
5.46
3.91
3.90
3.88
3.67
3.35
1.82
1.70
NO2
NO2
5-OR1-NRMe
CD3CN
CH3OH
H2O
1.01
1.00
2.05
1.02
1.05
1.07
1.04
1.08
2.00
1.08
1.08
2.06
2.10
3.06
3.14
3.05
3.05
3.02
3.16
3.01
9.0
8.5
8.0
7.5
7.0
6.5
6.0
5.5
5.0
4.5
4.0
3.5
3.0
2.5
2.0
1.5
1.0
0.5
f1 (ppm)

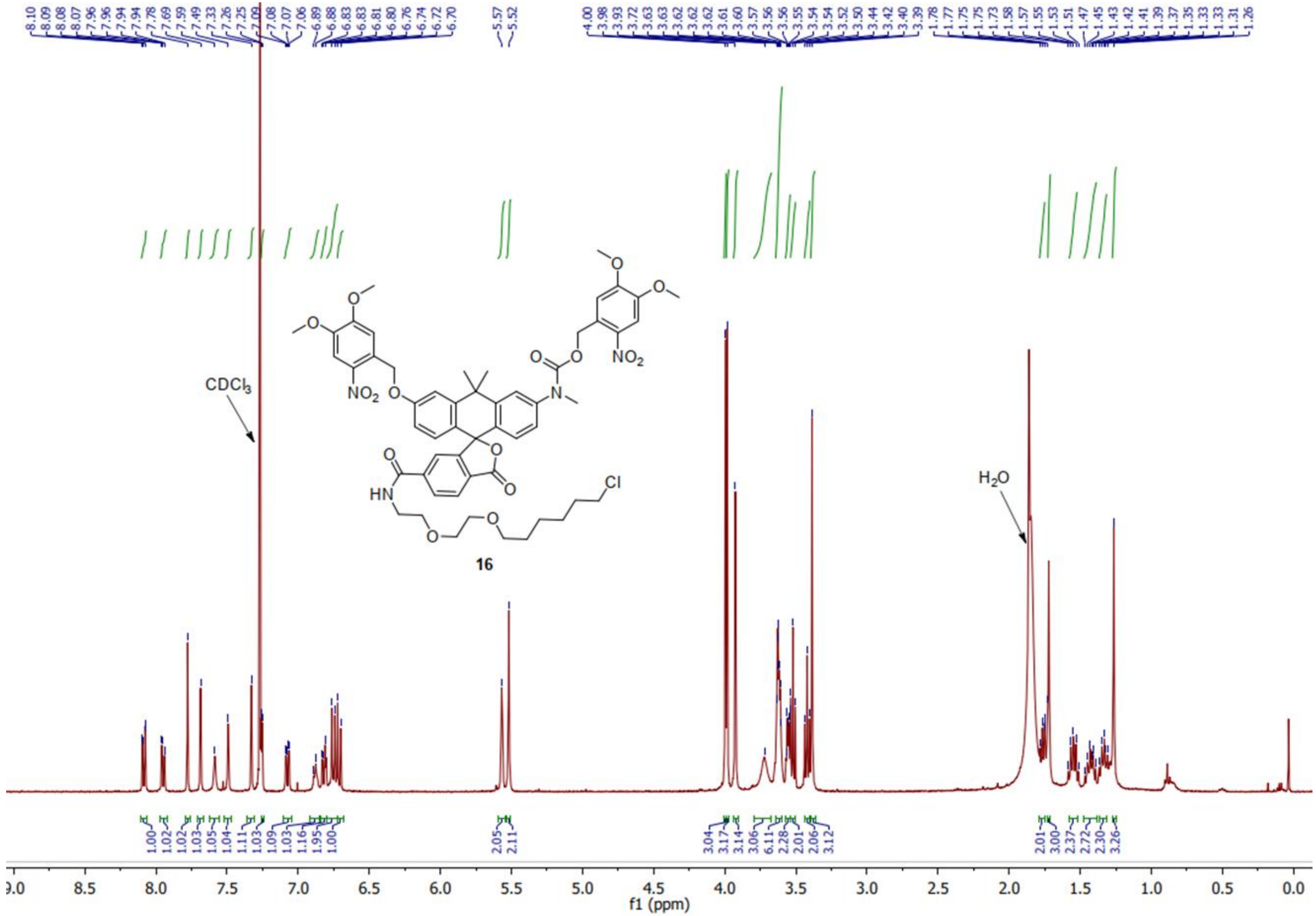
CDCl3
H2O
16
f1 (ppm)
